# A Ball‐Milling‐Enabled Reformatsky Reaction

**DOI:** 10.1002/cssc.201900886

**Published:** 2019-06-05

**Authors:** Qun Cao, Roderick T. Stark, Ian A. Fallis, Duncan L. Browne

**Affiliations:** ^1^ School of Chemistry Cardiff University Main Building, Park Place Cardiff CF10 3AT UK

**Keywords:** ball milling, mechanochemistry, organozinc formation, reformatsky reaction

## Abstract

An operationally simple one‐jar one‐step mechanochemical Reformatsky reaction using in situ generated organozinc intermediates under neat grinding conditions has been developed. Notable features of this reaction protocol are that it requires no solvent, no inert gases, and no pre‐activation of the bulk zinc source. The developed process is demonstrated to have good substrate scope (39–82 % yield) and is effective irrespective of the initial morphology of the zinc source.

Metal‐mediated C−C bond formation is an essential tool in modern organic synthesis. Numerous reactions consisting of metal‐mediated nucleophilic addition to electrophiles have been developed for the synthesis of complex organic molecules.^1^ However, the generally high basicity and/or nucleophilicity of some organometallic reagents restricts their use in late‐stage modification, where sensitive functional groups may already exist in the chemical structure. Conversely, organozinc species represent a class of “mild” organometallic compounds that demonstrate excellent functional group compatibility.^2^ Nevertheless, the preparation of organozinc species often requires initial access to more reactive organometallics, which are then transmetalated to give the desired organozinc reactant by metathesis with Zn^II^ salts. Alternatively, activated Zn^0^ can be used for the oxidative addition into carbon–halogen bonds (Scheme 1 A). In general the formation and manipulation of organometallic compounds is not particularly clean or green when considering that solvents often have to be distilled and dried prior to use; inert gases are commonly required, and, in the case of organozinc reagents, the form of the bulk metal can play an important role and chemical additives are typically required to generate the activated zinc species.^3^ Recently, we identified mechanochemistry and ball‐milling as a tool for the straightforward generation of organozinc species without the requirement for carefully prepared solvents or inert gases. Under these conditions the input of mechanical energy is enough to break down the resilient metal oxide surface and, in the presence of the alkyl/aryl halide, generate the corresponding organozinc species (Scheme 1 B).^4^ These organozinc species may then be intercepted by opening the grinding jar, adding both catalyst and coupling partner before then running a telescoped Negishi coupling reaction; such a process is applicable to both sp^3^−sp^3^ and sp^3^−sp^2^ coupling reactions. We have exploited this concept to carry out the one‐jar, one‐step preparation and use of organozinc species in a robust mechanochemical Reformatsky reaction (Scheme 1 C). The Reformatsky reaction offers excellent potential for the formation of C−C bonds through (1) predictable C−C formation, (2) neutral reaction conditions (in comparison to obtaining the same products through aldol condensation), (3) broad functional group tolerance, and (4) the ability to impart a high degree of stereocontrol.^5^

**Scheme 1:**
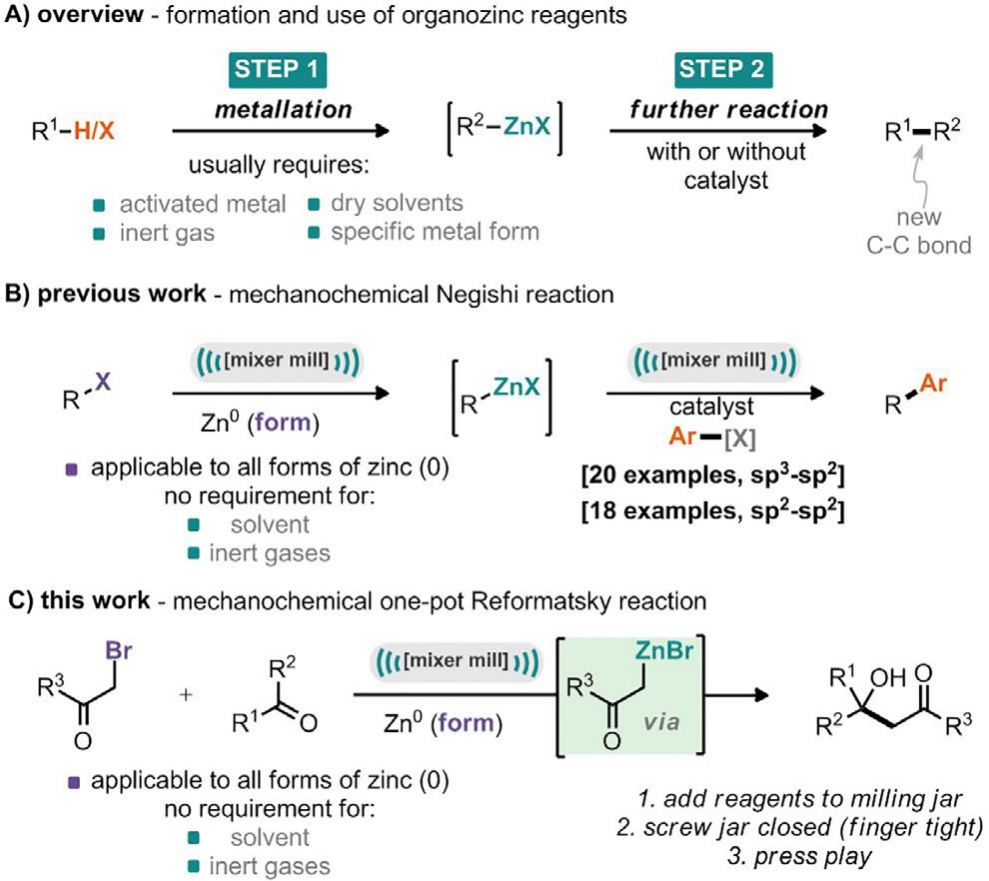
A) Formation and use of organzinc reagents. B) Previous work: one‐jar two‐step Negishi coupling.^4^ C) This work: one‐jar one‐step mechanochemical Reformatsky reaction.

The classical Reformatsky reaction, in which β‐hydroxy esters are formed by the reaction of aldehydes/ketones with α‐halo esters in the presence of metallic zinc, was reported in 1887.^6^ Since this seminal work, a variety of latent nucleophiles and electrophiles have been studied and applied in this reaction and it has been routinely used in the synthesis of complex natural products.^7^ However, to carry out the Reformatsky reaction, organozinc reagents must be prepared at the point of use; the majority of organozinc reagents are not commercially available.^8^ This process can be problematic, owing to the formation of a layer of passivating zinc oxide on the surface of zinc powder, which hampers the formation of organozinc species and requires removal through treatment with chemical additives. Such additives include aqueous acid,^9^ iodine,^10^ 1,2‐dibromoethane,^11^ or chlorotrimethylsilane.^12^ Highly reactive Rieke zinc is an alternative, but its preparation is nontrivial and requires the reduction of ZnCl_2_ with alkali metals (Li, Na, K) and naphthalene.^13^ Furthermore, the air‐sensitivity of this method renders the green credentials of the Rieke approach poor, and the success of the outcome is highly dependent on the physical form of zinc used. Multiple different forms of zinc are commercially available (see the Supporting Information; Figure S1). Therefore, the focus of this work is on the mechanical activation of zinc, which not only renders the process more operationally simple and cost effective, but also delivers significant improvements to some of the green metrics typically associated with this reaction.^14^

Mechanochemistry has been widely used among the crystal engineering and metal–organic framework communities.^15^ Recently, ball milling and other mechanochemical techniques have been explored as methods to complement the synthetic toolkit.^16^ Running reactions under mechanochemical conditions not only offers a more sustainable way to carry out solvent‐minimized/free reactions but can also lead to decreased reaction times, increased selectivity, or different reaction outcomes when compared to results obtained from solution‐based reactions.^17^ Herein we describe a green method for the Reformatsky reaction by using the ball mill mechanical activation of elemental zinc in air.

Studies commenced by treating model substrates benzaldehyde (**1**, 1 mmol) and ethyl 2‐bromoacetate (**2**, 1.2 mmol) with 1.6 equivalents of zinc (20–30 mesh zinc granular) at 30 Hz in a 10 mL grinding jar with a single ball of mass 4 g (Table 1). After 2 hours of grinding, ^1^H NMR spectroscopy of the crude mixture (mesitylene as internal standard) confirmed that a 70 % yield of ethyl 3‐hydroxy‐3‐phenylpropanoate (**3**) was produced during the milling process (Table 1, entry 1).

**Table 1:** Optimization of one‐jar one‐step Reformatsky reaction using a ball mill. 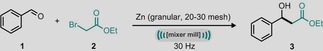

Entry	Zn [equiv]	**2** [equiv]	*t* [h]	Conv. [%]^[a]^	Yield [%]^[a]^
1	1.6	1.2	2	96	70
2	2	1.2	2	97	81 (72)
3	3	1.2	2	98	81
4	5	1.2	2	100	82
5	2	1.5	2	99	83
6	2	2.0	2	99	81
7	2	1.2	0.5	58	33
8	2	1.2	1	70	58
9	2	1.2	1.5	96	76
10	0	1.2	2	0	0

Reaction conditions: benzaldehyde (1 mmol), ethyl 2‐bromoacetate (as specified), 20–30 mesh zinc granular (as specified), mixer mill, 30 Hz. [a] Conversion and yield were determined by ^1^H NMR using mesitylene as internal standard; value in parentheses refers to yield of isolated product.

Increasing the amount of zinc to 2 equivalents resulted in an 81 % yield of the desired hydroxy ester product **3** (Table 1, entry 2). Further increasing the amount of zinc led to no significant increase in yield (Table 1, entries 3 and 4). Rather than increasing the equivalents of zinc, increasing the amount of ethyl 2‐bromoacetate (**2**) from 1.2 to 2.0 equivalents also led to no real difference in the observed yield (Table 1, entries 5 and 6). With the optimized ratio of reagents in hand, a reaction time study assessed four individual reaction times of 0.5, 1, 1.5, and 2 h (Table 1, entries 2 and 7–9), which indicated that the reaction needs 2 h to afford complete conversion. A control experiment, whereby zinc was omitted from the reaction, returned none of the desired product.

We then applied the optimized conditions to a further 11 commercially available forms of zinc (Figure S1). Although, the zinc forms had various particle sizes, which may lead to differences in the ratio between zinc oxide layer and zinc metal, a fixed mass (2 mmol, 0.130 g) of each sample was employed. Pleasingly, we found that in all cases the mechanochemical Reformatsky reaction was successful irrespective of form of zinc that was used (Table 2). Notably, there appears to be a general trend that the forms with a higher surface area/volume ratio performed better for the Reformatsky reaction under neat ball‐milling conditions, this is perhaps contrary to prediction as these metal forms should also contain a higher proportion of zinc oxide.

**Table 2:** Mechanochemical Reformatsky reaction using different zinc forms. 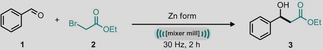

Entry	Zn form	Supplier	Conv. [%]^[a]^	Yield [%]^[a]^
1	zinc granular, 20–30 mesh	Sigma–Aldrich	97	81
2	zinc granular, 20 mesh	Sigma–Aldrich	99	72
3	zinc foil, 0.25 mm thick, 99.9 %	Sigma–Aldrich	99	77
4	zinc dust, <10 mm	Sigma–Aldrich	98	80
5	zinc puriss, ACS reagent, >99.9 %	Sigma–Aldrich	91	66
6	zinc shot, 10 mm dia., 2 mm thick, 99.99 %	Sigma–Aldrich	87	53
7	zinc flake, ca. 325 mesh, 99.9 %	Alfa Aesar	99	87
8	zinc wire, 0.04 in dia., 99.95 %	Alfa Aesar	95	75
9	zinc powder, 6–9 μm, 97.5 %	Alfa Aesar	99	83
10	zinc metal powder	Fisher Scientific	98	68
11	zinc, ≥99%, mossy	Acros	96	73
12	zinc foil, 0.38 mm	BDH chemicals	99	73

Reaction conditions: benzaldehyde (1 mmol), ethyl 2‐bromoacetate (1.2 mmol), zinc form as specified (2 equiv), mixer mill, 30 Hz, 2 h. [a] Conversion and yield were determined by ^1^H NMR spectroscopy with mesitylene as an internal standard.

With the optimized conditions established, application to a small range of 11 carbonyl compounds was investigated for reactivity with ethyl 2‐bromoacetate and zinc flakes (ca. 325 mesh) under mechanical grinding (Scheme 2). We found that the mechanochemical Reformatsky reaction demonstrates good functional group tolerance. The highly sterically hindered substrate 2,4,6‐trimethylbenzaldehyde afforded the corresponding hydroxy ester, **5**, in good yield (66 %). Halogen‐containing aromatic aldehydes were also effective substrates, leading to good yields of the isolated halogen‐containing products (Scheme 2, **6**–**8**, 77–82 %). Hydrodehalogenation was not observed under these one‐pot reaction conditions. Acetophenone was also a competent electrophile under these conditions, affording the isolated tertiary benzylic alcohol product **10** in 69 % yield. Aliphatic aldehydes offered mixed results. Cyclohexylcarboxaldehyde gave the product in 39 % yield, 2‐phenylethyl aldehyde afforded 73 % yield of the corresponding product, and cinnamaldehyde resulted in 61 % yield (Scheme 2).

**Scheme 2:**
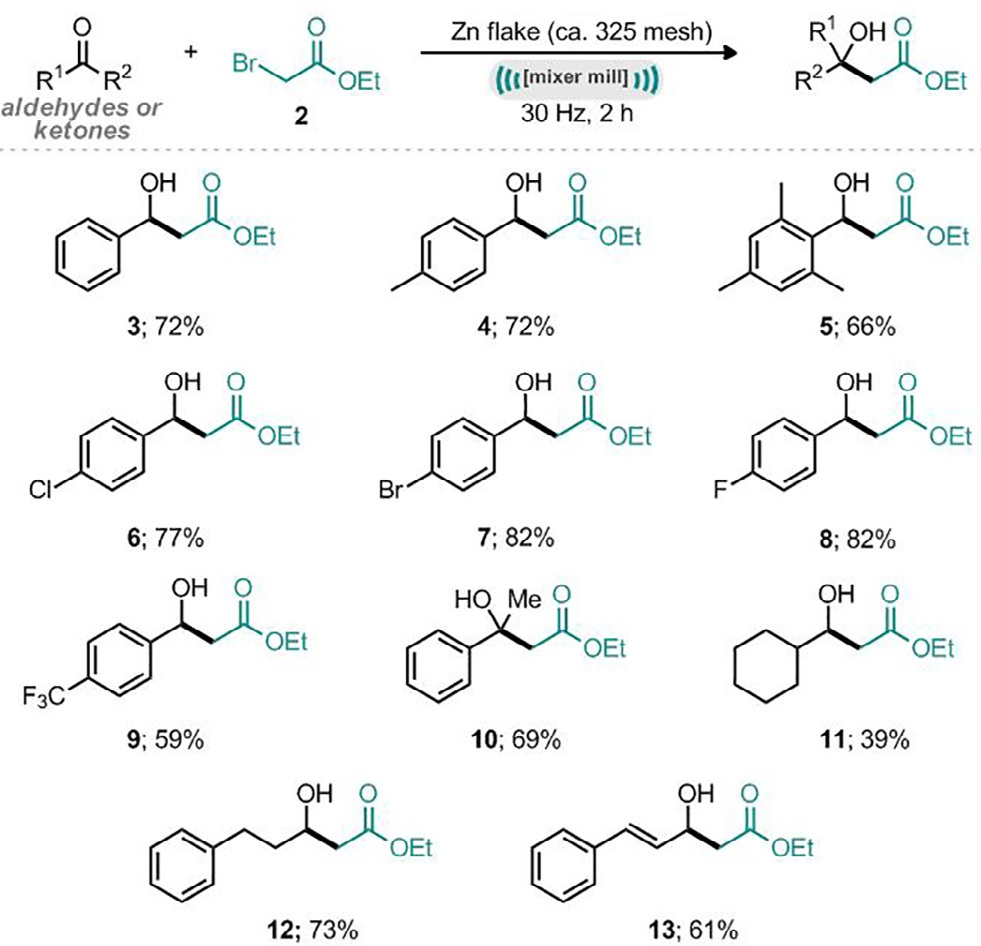
Scope of carbonyl electrophiles in the mechanochemical Reformatsky reaction. Reaction conditions: aldehyde/ketone (1 mmol), ethyl 2‐bromoacetate (1.2 mmol), zinc flake (ca. 325 mesh, 2 equiv), mixer mill, 30 Hz, 2 h. Yields refer to isolated products.

Eight different latent nucleophiles were also examined in this process with benzaldehyde as the model electrophile. Pleasingly, the corresponding organozinc intermediates could be generated under mechanochemical conditions (Scheme 3) and both methyl and *tert*‐butyl α‐halo esters could be used to effectively form the β‐hydroxy esters **14** and **15** in good to excellent yields. Reaction with ethyl 2‐bromoproprionate provided 67 % yield of **16** as a mixture of diastereoisomers (*syn*/*anti*=58:42). Reaction with more stereocongested ethyl α‐bromoisobutyrate maintained a good yield of 68 % (**17**, Scheme 3) and α,α‐difluoro‐β‐hydroxyester **18** could also be prepared by this method. The ball‐milling‐enabled Reformatsky reaction with ethyl 4‐bromobut‐2‐enoate resulted in 52 % yield of the α‐substituted product **19** with moderate diastereoselectivity (*syn*/anti=62:38). 2‐Bromoacetonitrile also participated in the in situ generation of an organozinc reagent and formed the corresponding Reformatsky product 3‐hydroxy‐3‐phenylpropanenitrile (**20**). Notably, in all cases explored, no reductive aldehyde coupling (pinacol reaction) was observed.

**Scheme 3:**
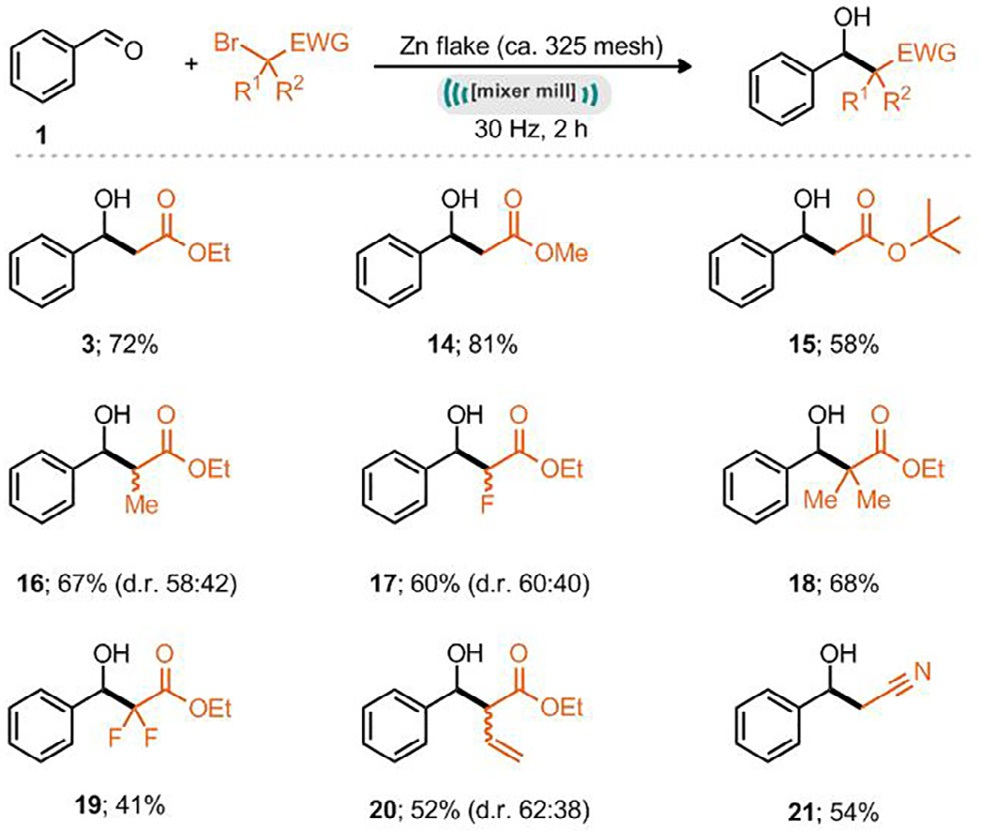
Scope of latent nucleophiles in the mechanochemical Reformatsky reaction. Reaction conditions: aldehyde/ketone (1 mmol), ethyl 2‐bromoacetate (1.2 mmol), zinc flake (ca. 325 mesh, 2 equiv), mixer mill, 30 Hz, 2 h. Yields refer to isolated products.

The applicability of imines as electrophiles was also briefly explored under the developed ball‐milling conditions. Besides aldehydes and ketones, nonclassical Reformatsky electrophiles such as azomethines, nitriles, lactones, anhydrides, *Y*‐thioalactams, and amides can also be used for the Reformatsky reaction.^18^ Although not fully optimized, *N*‐benzylideneaniline (**22**) underwent a mechanochemical Reformatsky reaction (Scheme 4 A) to afford the β‐amino ester **23** in 48 % yield, alongside a small amount of the corresponding β‐lactam **24** (7 % yield).

**Scheme 4:**
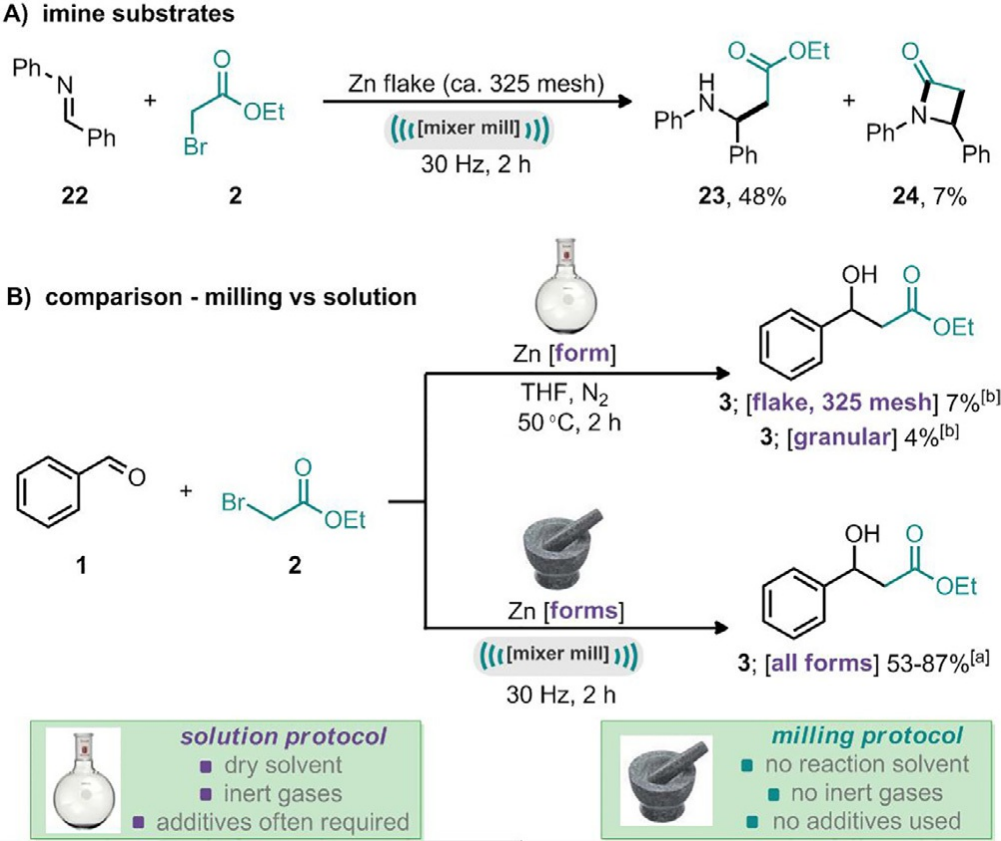
A) Example of mechanochemical Reformatsky‐type reaction with imine electrophile. B) Reformatsky reaction with unactivated zinc source: ball‐milling enabled reaction vs. solution‐based reaction. [a] Yield determined by ^1^H NMR spectroscopy with mesitylene as internal standard. [b] Yield determined by GC with mesitylene as internal standard.

The convenience of the method is well demonstrated by comparing a solution‐based reaction with that under ball‐milling conditions using identical reagents (Scheme 4 B). The solution‐based reaction in dry THF and under nitrogen atmosphere at 50 °C with either zinc flakes or granular zinc (no additive used) resulted in low yields of 7 and 4 %, respectively (Scheme 4 B), whereas under ball‐milling conditions without any solvent, inert gas, or additive (Table 2), the reaction proceeded smoothly in 2 h and all forms of zinc explored were effective for this transformation.

In conclusion, a reliable, operationally simple one‐jar one‐step mechanochemical Reformatsky reaction has been developed. By utilizing the organozinc generated in situ upon milling, this method avoids the requirement for dry solvents, inert gases, and chemical additives and thus furnishes a process where the green metrics are improved.

## Conflict of interest

*The authors declare no conflict of interest*.

## Supporting information

As a service to our authors and readers, this journal provides supporting information supplied by the authors. Such materials are peer reviewed and may be re‐organized for online delivery, but are not copy‐edited or typeset. Technical support issues arising from supporting information (other than missing files) should be addressed to the authors.

Supplementary
