# Supplementary material for: A Ball‐Milling‐Enabled Reformatsky Reaction
Source: ChemSusChem. 2019 Jun 5;12(12):2554–7. doi: 10.1002/cssc.201900886 (PMC6619031; doi:10.1002/cssc.201900886)
Supplement: Supplementary file 1 — Supplementary [file CSSC-12-2554-s001.pdf]

CHEMISTRY & SUSTAINABILITY
CHEMSUSCHEM
ENERGY & MATERIALS

Supporting Information

A Ball-Milling-Enabled Reformatsky Reaction

Qun Cao, Roderick T. Stark, Ian A. Fallis, and Duncan L. Browne*[a]

cssc_201900886_sm_miscellaneous_information.pdf

Supporting Information

A Ball-Milling Enabled Reformatsky Reaction

Qun Cao,[†] Roderick T. Stark,[†] Ian A. Fallis[†] and Duncan L. Browne[†,*]

[†] School of Chemistry, Cardiff University, Main Building, Park Place, Cardiff, CF10 3AT, UK.

E-mail: dlbrowne@cardiff.ac.uk

Table of Contents

1. General Information S2
2. Different Commercial Available Zinc Forms…………..…………………………………..S4
3. Experimental Procedures S4
4. Product Characterization Data………………………………………………………S5
5. NMR Data..S13
6. References...S40

1 General Information

Unless otherwise stated, all reagents were purchased from commercial sources and used without further purification. Different zinc forms were purchased from the following: (1) Zinc granular (20-30 mesh, ACS reagent, ≥98.8%; Sigma-Aldrich). (2) Zinc granular (20 mesh, Sigma-Aldrich). (3) Zinc foil (thickness 0.25 mm, 99.9% trace metals basis; Sigma-Aldrich). (4) Zinc dust (< 10 μm, ≥98%; Sigma-Aldrich). (5) Zinc puriss.p.a. (ACS reagent, ≥99.9%, granular; Sigma-Aldrich). (6) Zinc shot, (10 mm (0.4 in) dia x 2 mm (0.08 in) thick, 99.99%, Alfa Aesar) (7) Zinc flake (-325 mesh, 99,9% (metal basis); Alfa Aesar). (8) Zinc wire (1.0 mm (0.04 in) dia, 99.95%, Alfa Aesar). (9) Zinc powder (average 6-9 micro, 97.5%, Alfa Aesar). (10) Zinc Powder (Certified AR for Analysis, metal, Fisher Chemical). (11) Zinc mossy (+99%, ACROS Organics). (12) Zinc foil (about 0.38 mm, Fisher Scientific).

Thin layer chromatography (TLC) was carried out using Merck TLC silica gel 60 sheet, and visualized with ultraviolet light or potassium permanganate stain. Flash column chromatography (FCC) was performed with Sigma Aldrich silica gel 40-60 Å as the stationary phase and solvents employed were analytical grade. ^{1}H NMR spectra were recorded on a Bruker AVX500 (500 MHz) spectrometer at ambient temperature. ^{13}C NMR spectra were recorded on a Bruker AVX500 (125 MHz) spectrometer at ambient temperature. ^{19}F NMR spectra were recorded on a Bruker AVX500 (471 MHz) spectrometer at ambient temperature. Melting points were measured on a Gallenkamp melting point apparatus and are reported corrected by linear calibration to benzophenone (47 - 49 °C) and benzoic acid (121 - 123 °C).

High resolution mass spectroscopy (HRMS) data was obtained on a Thermo Scientific LTQ Orbitrap XL by the EPSRC UK National Mass Spectrometry Facility at Swansea University or on a Waters MALDI-TOF mx in Cardiff University. Spectra were obtained using electron impact ionization (EI), chemical ionization (CI), positive electrospray (ESI+), pneumatically-assisted electrospray (pNSI) or atmospheric solids analysis probe (ASAP+). Infrared spectra were recorded on a Shimadzu IR-Affinity-1S FTIR spectrometer.

Gas chromatography analysis was carried out using a Bruker Scion 456 gas chromatograph. An Agilent 19091J-413HP-5 column (30.0 m × 320 μm × 0.25 μm nominal) was employed for all of the separations using the following conditions: initial column temperature, 40 °C; initial hold time, 2 min; next temperature, 100 °C; hold time, 5 min; rate of temperature ramp 1, 4 °C/min, final temperature 300 °C; hold time, 5 min; rate of temperature ramp 2, 15 °C/min; injection temperature, 250 °C; injection volume 1 μL; detection temperature, 300 °C, split mode. The effluent was combusted in an H_2/air flame and detected using FID (flame ionization detector).

The ball mill used was a Retsch MM 400 mixer mill. Unless otherwise stated, mechanochemical reactions were performed in 10 mL stainless steel jars from Retsch with one stainless steel ball of mass 4 g. The longest time that this mill can be programmed to run for is 99 minutes. In order to run longer reaction times the mill was started, and then additional time added to the timer in order to ensure that the mill was running continuously for the desired reaction time.

The GC yield of products and conversion of substrates were determined by using the internal standard method. The response factor (RF) of analytes was determined by analyzing known quantities of internal standard (mesitylene) against known quantities of substrate and product:

$$RF = \frac{Area_{internal\ standard} \times Moles_{analyte}}{Area_{analyte} \times Moles_{internal\ standard}}$$

The quantity of an analyte was then calculated according to the following equation:

$$Moles_{analyte} = \frac{RF \times Moles_{internal\ standard} \times Area_{analyte}}{Area_{internal\ standard}}$$

2 Different Commercial Available Zinc Forms

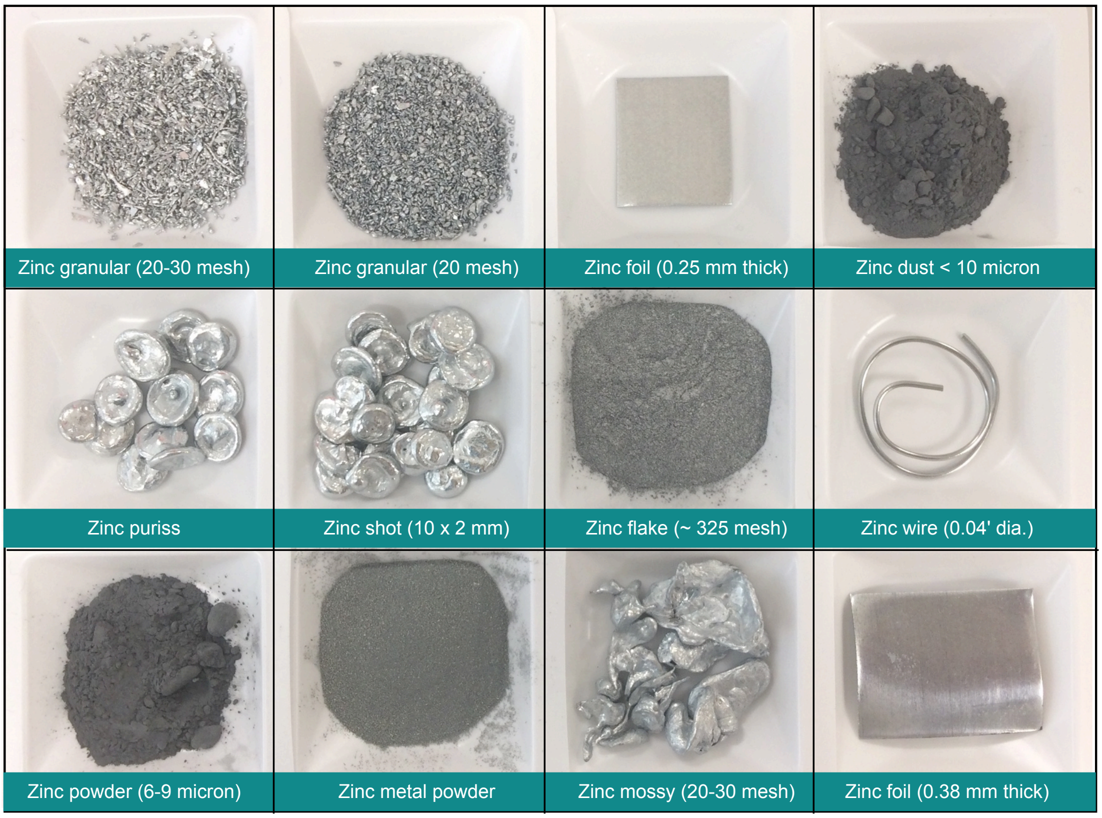

Figure S1: Examples of different commercial available zinc forms

3 Experimental Procedures

General Procedure for Mechanochemical Reformatsky Reaction

To a 10 mL Retsch stainless steel jar was added zinc (2 mmol, 0.131 g), aldehyde (1 mmol), α-bromo ester (1.2 mmol) under air atmosphere. A stainless steel ball of mass 4.0 g was added and the mixture was milled at 30 Hz for 2 hours. The resulting black/grey paste mixture was transferred into a flask and the jar was rinsed with ethyl acetate (2 x 10 mL), before quenching with 2 M HCl solution (10 mL). The quenched solution was then washed and extracted with EtOAc, The combined organic layers were dried over $MgSO_4$ and concentrated *in vacuo*.

(1) To obtain the ^1H NMR yield and conversion, mesitylene (0.5 mmol, 70 μL) and $CDCl_3$ (5 mL) were added to the concentrated mixture. Then approximately 1 mL sample of this mixture was taken for ^1H NMR analysis. The yield was calculated relative to mesitylene (internal standard).

(2) To obtain the isolated yield, the concentrated reaction mixture was purified by silica gel flash chromatography using the noted solvent systems.

General Procedure for Reformatsky Reaction using Conventional Solution Method using Unactivated Zinc

To an oven dried flask containing 20 - 30 mesh zinc granular or 325 mesh zinc flake (2 mmol, 0.131 g) in dry THF (10 mL) was added benzaldehyde (1 mmol), ethyl 2-bromoacetate (1.2 mmol) and mesitylene (0.5 mmol, 70 µL) under nitrogen. The reaction was then heated to 50°C for 2 hours, before quenching with 2 M HCl solution (10 mL) and further stirring. The quenched mixture was washed and extracted with EtOAc, dried over $MgSO_4$ and concentrated *in vacuo*. A sample was taken and submitted for GC analysis.

4 Product Characterization Data

ethyl 3-hydroxy-3-phenylpropanoate (3)

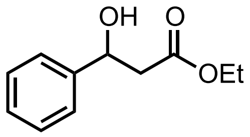

Purified by column chromatography (Ethyl Acetate/Petroleum ether = 1:4) to give the title compound (0.140 g, 72%) as a light yellow oil. **^{1}H NMR** (500 MHz, $CDCl_3$) δ 7.43 - 7.35 (m, 4H), 7.34 - 7.27 (m, 1H), 5.16 (dd, *J* = 8.9, 3.9 Hz, 1H), 4.21 (q, *J* = 7.1 Hz, 2H), 2.85 - 2.65 (m, 2 H), 1.29 (t, *J* = 7.1 Hz, 3H). **^{13}C NMR** (126 MHz, $CDCl_3$) δ 172.6, 142.6, 128.7, 128.0, 125.8, 70.5, 61.0, 43.5, 14.3. NMR data is consistent with literature values.[1] **HRMS** (EI) calcd for [M] = $C_{11}H_{14}O_3$: 194.0943, found: 194.0944.

ethyl 3-hydroxy-3-(p-tolyl)propanoate (4)

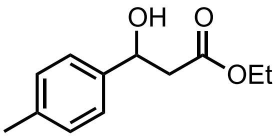

Purified by column chromatography (Ethyl Acetate/Petroleum ether = 1:4) to give the title compound (0.150 g, 72%) as a light yellow oil. **^{1}H NMR** (500 MHz, $CDCl_3$) δ 7.34 - 7.25 (m, 2H), 7.22 - 7.14 (m, 2H), 5.13 (dd, *J* = 9.2, 3.7 Hz, 1H), 4.21 (q, *J* = 7.2 Hz, 2H), 2.83 - 2.66 (m, 2 H), 2.37 (s, 3H), 1.29 (t, *J* = 7.1 Hz, 3H). **^{13}C NMR** (126 MHz, $CDCl_3$) δ 172.6, 139.7, 137.6, 129.4, 125.8, 70.3, 61.0, 43.5, 21.3, 14.3. NMR data is consistent with literature values.[1] **HRMS** (EI) calcd for [M] = $C_{12}H_{16}O_3$: 208.1099, found: 208.1090.

ethyl 3-hydroxy-3-mesitylpropanoate (5)

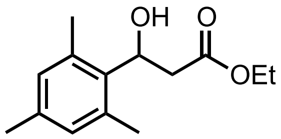

Purified by column chromatography (Ethyl Acetate/Petroleum ether = 1:10) to give the title compound (0.155 g, 82%) as a light yellow oil. **^{1}H NMR** (500 MHz, $CDCl_3$) δ 6.85 - 6.77 (m, 2H), 5.60 (dd, *J* = 10.7, 3.1 Hz, 1H), 4.21 (qd, *J* = 7.2, 0.6 Hz, 2H), 3.05 (dd, *J* = 16.6, 10.7 Hz, 1H), 2.76 (br s, 1H), 2.54 (dd, *J* = 16.6, 3.1 Hz, 1H), 2.42 (s, 6H), 2.25 (s, 3H), 1.29 (t, *J* = 7.2 Hz, 3H). **^{13}C NMR** (126 MHz, $CDCl_3$) δ 172.8, 137.1, 136.2, 134.7, 130.3, 67.7, 61.0, 40.2, 20.9, 20.8, 14.3. NMR data is consistent with literature values.[2] **HRMS** (EI) calcd for [M] = $C_{14}H_{20}O_3$: 236.1412, found: 236.1415.

ethyl 3-(4-chlorophenyl)-3-hydroxypropanoate (6)

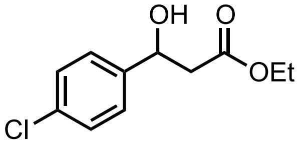

Purified by column chromatography (Ethyl Acetate/Petroleum ether = 1:4) to give the title compound (0.176 g, 77%) as a light yellow oil. **^{1}H NMR** (500 MHz, $CDCl_3$) δ 7.35 - 7.28 (m, 4H), 5.14 - 5.06 (m, 1H), 4.21 (qd, *J* = 7.2, 2.0 Hz, 2H), 2.72 - 2.66 (m, 2H), 1.26 (td, *J* = 7.1, 1.5 Hz, 3H). **^{13}C NMR** (126 MHz, $CDCl_3$) δ 172.4, 141.1, 133.6, 128.8, 127.2, 69.8, 61.2, 43.3, 14.3. NMR data is consistent with literature values.[1] **HRMS** (EI) calcd for [M] = $C_{11}H_{13}O_3Cl$: 228.0553, found: 228.0559.

ethyl 3-(4-bromophenyl)-3-hydroxypropanoate (7)

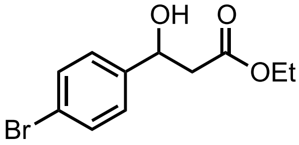

Purified by column chromatography (Ethyl Acetate/Petroleum ether = 1:4) to give the title compound (0.224 g, 82%) as a light yellow oil. **^{1}H NMR** (500 MHz, $CDCl_3$) δ 7.59 - 7.46 (m, 2H), 7.32 - 7.22 (m, 2H), 5.11 (dd, *J* = 7.9, 4.9 Hz, 1H), 4.21 (q, *J* = 7.1 Hz, 2H), 3.41 (br s, 1H), 2.77 - 2.64 (m, 2 H), 1.29 (t, *J* = 7.2 Hz, 3H). **^{13}C NMR** (126 MHz, $CDCl_3$) δ 172.4, 141.6, 131.8, 127.6, 121.7, 69.8, 61.2, 43.3, 14.3. NMR data is consistent with literature values.[1] **HRMS** (ESI+) calcd for $[M-H_2O+H]^+$ = $C_{11}H_{12}O_2Br$: 255.0021, found: 255.0029.

ethyl 3-(4-fluorophenyl)-3-hydroxypropanoate (8)

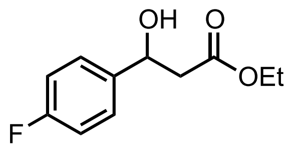

Purified by column chromatography (Ethyl Acetate/Petroleum ether = 1:4) to give the title compound (0.190 g, 82%) as a light yellow oil. **^{1}H NMR** (500 MHz, $CDCl_3$) δ 7.43 - 7.32 (m, 2H), 7.12 - 6.99 (m, 2H), 5.19 - 5.08 (m, 1H), 4.21 (q, *J* = 7.1 Hz, 2H), 3.34 (d, *J* = 3.4 Hz, 1H), 2.80 - 2.66 (m, 2 H), 1.29 (t, *J* = 7.1 Hz, 3H). **^{13}C NMR** (126 MHz, $CDCl_3$) δ 172.5, 162.4 (d, *J* = 246 Hz), 138.4 (d, *J* = 3 Hz), 127.5 (d, *J* = 8Hz), 115.5 (d, *J* = 21 Hz), 69.8, 61.1, 43.4, 14.3. **^{19}F NMR** (470 MHz, $CDCl_3$) δ - 114.8 NMR data is consistent with literature values.[1] **HRMS** (EI) calcd for [M] = $C_{11}H_{13}O_3F$: 212.0849, found: 212.0842.

ethyl 3-hydroxy-3-(4-(trifluoromethyl)phenyl)propanoate (9)

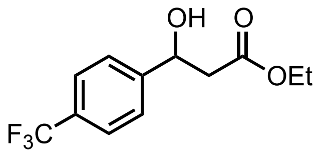

Purified by column chromatography (Ethyl Acetate/Petroleum ether = 1:4) to give the title compound (0.155 g, 59%) as a light yellow oil. **^{1}H NMR** (500 MHz, $CDCl_3$) δ 7.66 - 7.42 (m, 4H), 5.19 (td, 6.6, 2.9 Hz, 1H), 4.19 (q, *J* = 7.1 Hz, 2H), 3.52 (d, *J* = 3.5 Hz, 1H), 2.77 - 2.66 (m, 2H), 1.27 (t, *J* = 7.2 Hz, 3H). **^{13}C NMR** (126 MHz, $CDCl_3$) δ 172.3, 146.5, 130.4 (q, *J* = 33 Hz), 126.1, 125.6 (q, *J* = 4 Hz), 124.2 (q, 272 Hz), 69.8, 61.3, 43.2, 14.3. **^{19}F NMR** (470 MHz, $CDCl_3$) δ - 62.6. NMR data is consistent with literature values.[3] **HRMS** (EI) calcd for [M] = $C_{12}H_{13}O_3F_3$: 262.0817, found: 262.0818.

ethyl 3-hydroxy-3-phenylbutanoate (10)

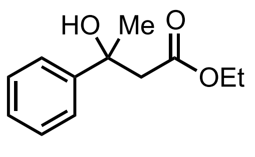

Purified by column chromatography (Ethyl Acetate/Petroleum ether = 1:5) to give the title compound (0.144 g, 69%) as a light yellow oil. **^{1}H NMR** (500 MHz, $CDCl_3$) δ 7.49 - 7.39 (m, 2H), 7.39 - 7.30 (m, 1H), 7.29 - 7.20 (m, 1H), 4.38 (br s, 1H), 4.06 (q, *J* = 7.1 Hz, 2H), 2.98 (d, *J* = 15.9 Hz, 1H), 2.79 (d, *J* = 15.9 Hz, 1H), 1.55 (s, 1H), 1.13 (t, *J* = 7.1 Hz, 1H). **^{13}C NMR** (126 MHz, $CDCl_3$) δ 172.9, 147.0, 128.4, 127.0, 124.6, 72.9, 60.9, 46.6, 30.8, 14.1. NMR data is consistent with literature values.[4] **HRMS** (EI) calcd for [M-H_2O] = $C_{12}H_{14}O_2$: 190.0994, found: 190.0993.

ethyl 3-cyclohexyl-3-hydroxypropanoate (11)

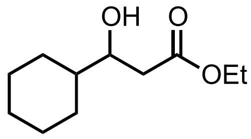

Purified by column chromatography (Ethyl Acetate/Petroleum ether = 1:3) to give the title compound (0.071 g, 39%) as a light yellow oil. **^{1}H NMR** (500 MHz, $CDCl_3$) δ 4.10 (q, *J* = 7.1 Hz, 2H), 3.70 (dt, *J* = 9.3, 6.0 Hz, 1H), 2.84 (br s, 1H), 2.53 - 2.27 (m, 1H), 1.86 - 1.52 (m, 5H), 1.37 - 0.87 (m, 9H). **^{13}C NMR** (126 MHz, $CDCl_3$) δ 173.6, 72.2, 60.7, 43.2, 38.7, 28.9, 28.3, 26.5, 26.3, 26.2, 14.3. NMR data is consistent with literature values.[5] **HRMS** (EI) calcd for [M-H_2O] = $C_{11}H_{18}O_2$: 182.1307, found: 182.1305.

ethyl 3-hydroxy-5-phenylpentanoate (12)

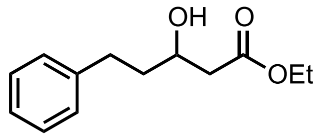

Purified by column chromatography (Ethyl Acetate/Petroleum ether = 1:4) to give the title compound (0.161 g, 73%) as a light yellow oil. **^{1}H NMR** (500 MHz, $CDCl_3$) δ 7.35 - 7.26 (m, 2H), 7.25 - 7.17 (m, 2H), 4.19 (q, *J* = 7.1 Hz, 2H), 4.04 (m, 1H), 3.08 (br s, 1H), 2.94 - 2.79 (m, 1H), 2.78 - 2.66 (m, 1H), 2.58 - 2.42 (m, 2H), 1.93 - 1.82 (m, 1H), 1.80 - 1.71 (m, 1H), 1.30 (t, *J* = 7.1 Hz, 3H). **^{13}C NMR** (126 MHz, $CDCl_3$) δ 173.2, 141.9, 128.6, 128.5, 126.0, 67.4, 60.9, 41.4, 38.3, 31.9, 14.3. NMR data is consistent with literature values.[1] **HRMS** (EI) calcd for [M] = $C_{13}H_{18}O_3$: 222.1256, found: 222.1265.

(E)-ethyl 3-hydroxy-5-phenylpent-4-enoate (13)

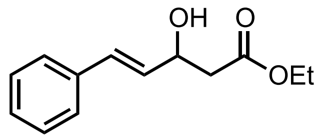

Purified by column chromatography (Ethyl Acetate/Petroleum ether = 1:4) to give the title compound (0.134 g, 61%) as a light yellow oil. **^{1}H NMR** (500 MHz, $CDCl_3$) δ 7.40 - 7.28 (m, 4H), 7.25 - 7.21 (m, 1H), 6.65 (d, *J* = 15.9 Hz, 1H), 6.22 (dd, *J* = 15.9, 6.1 Hz, 1H), 4.76 - 4.66 (m, 1H), 4.19 (q, *J* = 7.1 Hz, 2H), 3.04 (d, *J* = 4.0 Hz, 1H), 2.74 - 2.53 (m, 2H), 2.17 (t, *J* = 7.1 Hz, 3H). **^{13}C NMR** (126 MHz, $CDCl_3$) δ 172.4, 136.6, 130.9, 130.1, 128.7, 128.0, 126.7, 69.0, 61.0, 41.6, 14.3. NMR data is consistent with literature values.[1] **HRMS** (EI) calcd for [M] = $C_{13}H_{16}O_3$: 220.1099, found: 220.1096.

methyl 3-hydroxy-3-phenylpropanoate (14)

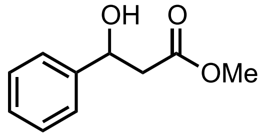

Purified by column chromatography (Ethyl Acetate/Petroleum ether = 1:5) to give the title compound (0.132 g, 81%) as a colourless oil. **^{1}H NMR** (500 MHz, $CDCl_3$) δ 7.24 - 7.07 (m, 5H), 5.00 - 4.95 (m, 1H), 3.56 (s, 3H), 3.02 (s, 1H), 2.77 - 2.44 (m, 2H). **^{13}C NMR** (126 MHz, $CDCl_3$) δ 172.8, 142.5, 128.6, 127.9, 125.7, 70.3, 51.9, 43.2. NMR data is consistent with literature values.[6] **HRMS** (EI) calcd for [M] = $C_{10}H_{12}O_3$: 180.0786, found: 180.0783.

tert-butyl 3-hydroxy-3-phenylpropanoate (15)

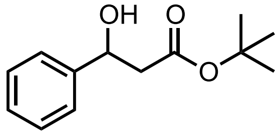

Purified by column chromatography (Ethyl Acetate/Petroleum ether = 1:5) to give the title compound (0.120 g, 58%) as a light yellow oil**. ^{1}H NMR** (500 MHz, $CDCl_3$) δ 7.35 - 7.09 (m, 5H), 4.97 (dd, *J* = 8.6, 4.1 Hz, 1H), 3.46 (br s, 1H), 2.70 - 2.38 (m, 2H), 1.35 (s, 9H). **^{13}C NMR** (126 MHz, $CDCl_3$) δ 171.8, 142.8, 128.5, 127.7, 125.8, 81.5, 70.5, 44.4, 28.1. NMR data is consistent with literature values.[7] **HRMS** (ESI+) calcd for $[M + Na]^+$ = $C_{13}H_{18}O_3Na$: 245.1154, found: 245.1153.

ethyl 3-hydroxy-2-methyl-3-phenylpropanoate (16)

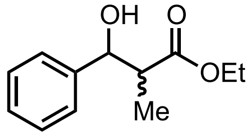

Purified by column chromatography (Ethyl Acetate/Petroleum ether = 1:10) to give two diastereomers as a light yellow oil with combined yield of 67% (0.140 g) with dr = 58:42**.** For Major diastereomers (syn): **^{1}H NMR** (500 MHz, $CDCl_3$) δ 7.29 - 7.24 (m, 5H), 5.00 (d, *J* = 4.3 Hz, 1H), 4.04 (q, *J* = 7.1 Hz, 2H), 2.95 (bs, 1H), 2.69 (qd, *J* = 7.1, 4.3 Hz, 1H), 1.12 (t, *J* = 7.1 Hz, 3H), 1.05 (d, *J* = 7.2 Hz, 3H) **^{13}C NMR** (126 MHz, $CDCl_3$) δ 176.0, 141.5, 128.3, 127.6, 126.1, 73.8, 60.9, 46.5, 14.2, 11.0. For minor diastereomers (anti): **^{1}H NMR** (500 MHz, $CDCl_3$) δ 7.50 - 7.15 (m, 5H), 4.75 (d, *J* = 8.3 Hz, 1H), 4.18 (q, *J* = 7.1 Hz, 2H), 3.06 (s, 1H), 2.80 (dq, *J* = 14.6, 7.2 Hz, 1H), 1.26 (t, *J* = 7.1 Hz, 3H), 1.01 (d, *J* = 7.2 Hz, 3H). **^{13}C NMR** (126 MHz, $CDCl_3$) δ 176.0, 141.7, 128.6, 128.2, 126.8, 76.5, 60.9, 47.3, 14.7, 14.3. NMR data is consistent with literature values.[8] **HRMS** (EI) calcd for [M-H_2O] = $C_{12}H_{14}O_2$: 190.0994, found: 190.0997.

Ethyl 3-hydroxy-2-fluoro-3-phenylpropanoate (17)

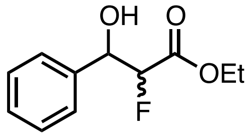

Purified by column chromatography (Ethyl Acetate/Petroleum ether = 1:4) to give the title compound (0.127 g, 60%) as a light yellow oil (mixture of diastereomers, *syn:anti* = 1:0.8, determined by ^{19}F NMR). **^{1}H NMR** (500 MHz, $CDCl_3$) δ 7.43 - 7.32 (m, 5H), 5.18 - 4.95 (m, 2H), 4.27 - 4.17 (m, 2H), 2.68 (bs, 1H), 1.21 (app. dt, *J* = 14.1, 7.1 Hz, 3H). **^{13}C NMR** (126 MHz, $CDCl_3$) δ 167.86 (d, *J* = 24.0 Hz), 167.85 (d, *J* = 23.1 Hz), 138.00 (d, *J* = 2.7 Hz), 137.60 (d, *J* = 2.9 Hz), 128.79, 128.76, 128.62, 126.90 (d, *J* = 1.0 Hz), 126.63 (d, *J* = 0.5 Hz), 91.59 (d, *J* = 192.0 Hz), 91.06 (d, *J* = 190.9 Hz), 74.06 (d, *J* = 20.1 Hz), 73.77 (d, *J* = 22.1 Hz), 62.07, 61.96, 14.11, 14.09. **^{19}F NMR** (471 MHz, $CDCl_3$): δ -197.7 (dd, *J* = 48.2, 16.2 Hz) [*erythro*], -202.8 (dd, *J* = 48.2, 22.3 Hz) [*threo*]. NMR data is consistent with literature values.[9] **HRMS** (ESI+) $[C_{11}H_{13}O_3F+Na]^+$ calc. 235.0746 found 235.0748.

ethyl 3-hydroxy-2,2-dimethyl-3-phenylpropanoate (18)

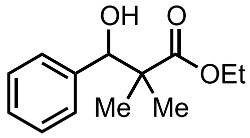

Purified by column chromatography (Ethyl Acetate/Petroleum ether = 1:10) to give the title compound (0.151 g, 68%) as a light yellow oil. **^{1}H NMR** (500 MHz, $CDCl_3$) δ 7.35 - 7.26 (m, 5H), 4.89 (s, 1H), 4.19 (q, *J* = 7.1 Hz, 2H), 1.27 (t, *J* = 7.1 Hz, 3H), 1.14 (s, 3H), 1.11 (s, 3H). **^{13}C NMR** (126 MHz, $CDCl_3$) δ 178.0, 140.0, 127.9, 127.8, 78.8, 61.1, 47.6, 23.3, 19.1, 14.3. NMR data is consistent with literature values.[10] **HRMS** (EI) calcd for [M] = $C_{13}H_{18}O_3$: 222.1256, found: 222.1255.

ethyl 2,2-difluoro-3-hydroxy-3-phenylpropanoate (19)

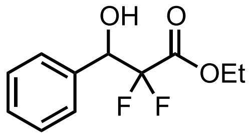

Purified by column chromatography (Ethyl Acetate/Petroleum ether = 1:5) to give the title compound (0.095 g, 41%) as a light yellow oil**.** **^{1}H NMR** (500 MHz, $CDCl_3$) δ 7.55 - 7.32 (m, 5H), 5.17 (dd, *J* = 15.3, 7.9 Hz, 1H), 4.31 (q, *J* = 7.1 Hz, 2H), 2.66 (br s, 1H), 1.29 (t, *J* = 7.2 Hz, 3H). **^{13}C NMR** (126 MHz, $CDCl_3$) δ 163.6 (t, *J* = 32 Hz), 134.6, 129.4, 128.6, 127.8, 113.9 (t, *J* = 256 Hz), 74.0 (t, *J* = 26 Hz), 63.2, 14.0. **^{19}F NMR** (470 MHz, $CDCl_3$) δ -113.9 (dd, 1F, J = 263.5, 7.9 Hz), -120.3 (dd, 1F, J = 263.5, 7.9 Hz) NMR data is consistent with literature values.[11] **HRMS** (EI) calcd for [M] = $C_{11}H_{12}O_3F_2$: 230.0755, found: 230.0751.

ethyl 2-(hydroxy(phenyl)methyl)but-3-enoate (20)

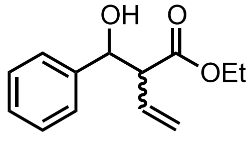

Purified by column chromatography (Ethyl Acetate/Petroleum ether = 1:20) to give two diastereomers as a light yellow oil with combined yield of 52% (0.115 g) with dr = 62:38. For Major diastereomers (*syn*): **^{1}H NMR** (500 MHz, $CDCl_3$) δ 7.29 - 7.24 (m, 4H), 7.22 - 7.19 (m, 1H), 5.90 (ddd, *J* = 17.2, 10.2, 8.9 Hz, 1H), 5.20 (dd, *J* = 10.2, 0.8 Hz, 1H), 5.11 (dt, 1H), 4.94 (d, *J* = 6.1 Hz, 1H), 3.99 (dq, *J* = 7.1, 0.8 Hz, 2H), 3.26 (dd, *J* = 8.9, 6.1 Hz, 1H), 2.46 (bs, 1H), 1.05 (t, *J* = 7.1 Hz, 3H) **^{13}C NMR** (126 MHz, $CDCl_3$) δ 172.6, 140.8, 132.1, 128.4, 128.0, 126.6, 120.7, 74.1, 61.1, 58.5, 14.1. For minor diastereomers (*anti*): **^{1}H NMR** (500 MHz, $CDCl_3$) δ 7.30 - 7.19 (m, 5H), 5.64 (ddd, *J* = 17.6, 10.0, 8.8 Hz, 1H), 5.00 (d, *J* = 9.7 Hz, 1H), 4.98 (d, *J* = 26.9 Hz, 1H), 4.86 (d, *J* = 8.1 Hz, 1H), 4.12 (q, *J* = 7.1 Hz, 2H), 3.37 (t, *J* = 8.3 Hz, 1H), 2.98 (s, 1H), 1.18 (t, *J* = 7.1 Hz, 3H) **^{13}C NMR** (126 MHz, $CDCl_3$) δ 173.1, 141.3, 132.4, 128.5, 128.1, 126.8, 119.5, 75.4, 61.2, 58.0, 14.2. NMR data is consistent with literature values.[12] **HRMS** (EI) calcd for [M + H] = $C_{13}H_{17}O_3$: 221.1172, found: 221.1172.

3-hydroxy-3-phenylpropanenitrile (21)

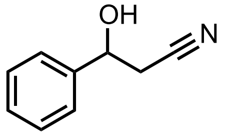

Purified by column chromatography (Ethyl Acetate/Petroleum ether = 1:3) to give the title compound (0.079 g, 54%) as a light yellow oil. **^{1}H NMR** (500 MHz, $CDCl_3$) δ 7.29 - 7.20 (m, 5H), 4.88 (t, *J* = 6.2 Hz, 1H), 2.81 (br s, 1H), 2.64 - 2.58 (m, 2H) **^{13}C NMR** (126 MHz, $CDCl_3$) δ 141.2, 129.0, 128.9, 125.6, 117.5, 70.1, 28.0. NMR data is consistent with literature values.[13] **HRMS** (EI) calcd for [M + NH_4] = $C_9H_{13}N_2O$: 165.1022, found: 165.1021.

ethyl 3-phenyl-3-(phenylamino)propanoate (23)

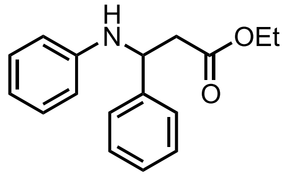

Purified by column chromatography (Ethyl Acetate/Petroleum ether = 1:10) to give the title compound (0.129 g, 48%) as an orange solid. **^{1}H NMR** (500 MHz, $CDCl_3$) δ 7.38 (d, *J* = 7.3 Hz, 2H), 7.32 (t, *J* = 7.6 Hz, 2H), 7.27 - 7.22 (m, 1H), 7.10 (dd, *J* = 8.3, 7.5 Hz, 2H), 6.67 (t, *J* = 7.3 Hz, 1H), 6.55 (d, *J* = 7.8 Hz, 2H), 4.82 (dd, *J* = 7.5, 6.0 Hz, 1H), 4.60 (bs, 1H), 4.16 - 4.04 (m, 2H), 2.85 - 2.73 (m, 2H), 1.19 (t, *J* = 7.1 Hz, 3H). **^{13}C NMR** (126 MHz, $CDCl_3$) δ 171.3, 146.9, 142.3, 129.3, 128.9, 127.6, 126.4, 117.9, 113.8, 61.0, 55.1, 43.1, 14.3. NMR data is consistent

with literature values.[14] **HRMS** (pNSI) calcd for $[M+H]^+$ = $C_{17}H_{20}O_2N$: 270.1489, found: 270.1491. Melting point = 77 - 79 °C.

1,4-diphenylazetidin-2-one (24)

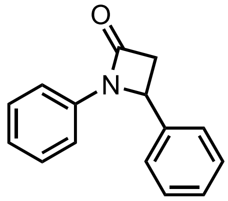

Purified by column chromatography (Ethyl Acetate/Petroleum ether = 1:10) to give the title compound (0. 016 g, 7%) as an orange solid**.** **^{1}H NMR** (500 MHz, $CDCl_3$) δ 7.39 - 7.32 (m, 5H), 7.30 - 7.27 (m, 2H), 7.26 - 7.22 (m, 2H), 7.04 (ddd, *J* = 8.6, 2.6, 1.3 Hz, 1H), 5.02 (dd, *J* = 5.7, 2.6 Hz, 1H), 3.56 (dd, *J* = 15.1, 5.7 Hz, 1H), 2.95 (dd, *J* = 15.1, 2.7 Hz, 1H) **^{13}C NMR** (126 MHz, $CDCl_3$) δ 164.8, 138.3, 137.9, 129.3, 129.2, 128.7, 126.0, 124.0, 116.9, 54.1, 47.2. NMR data is consistent with literature values.[15] **HRMS** (pNSI) calcd for $[M+H]^+$ = $C_{15}H_{14}ON$: 224.1070, found: 224.1070. Melting point = 138 - 140 °C.

5 NMR Spectra

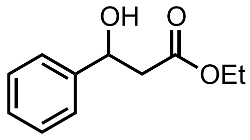

3

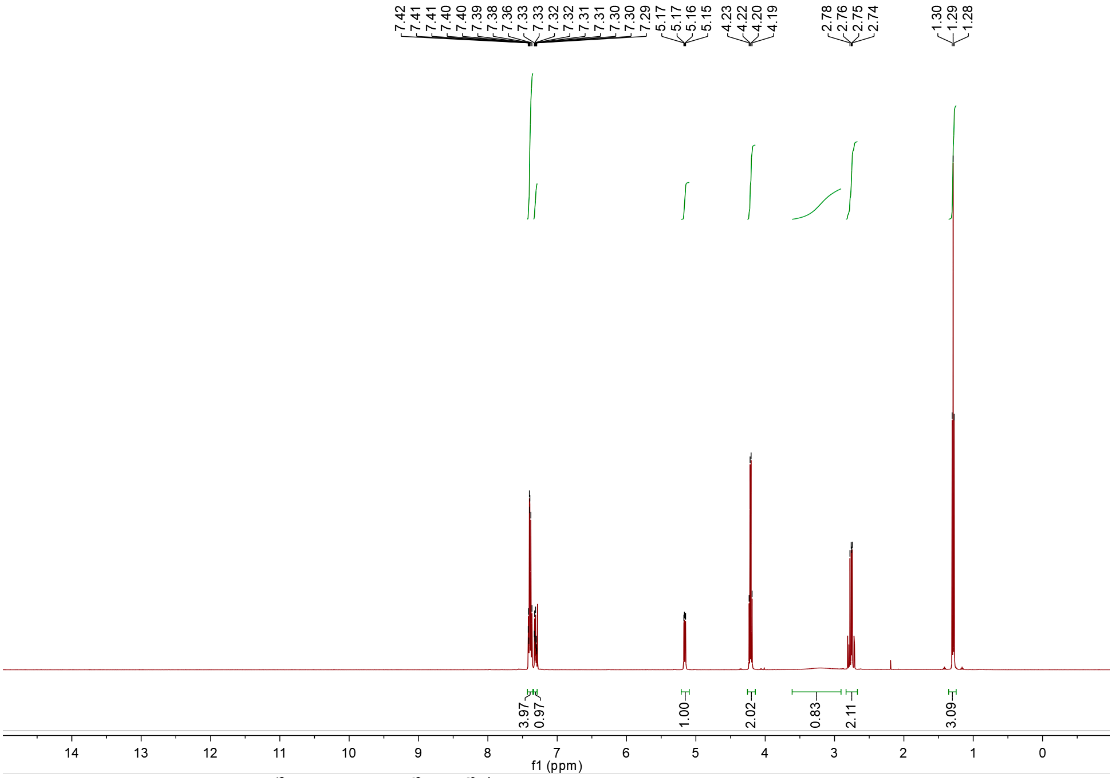

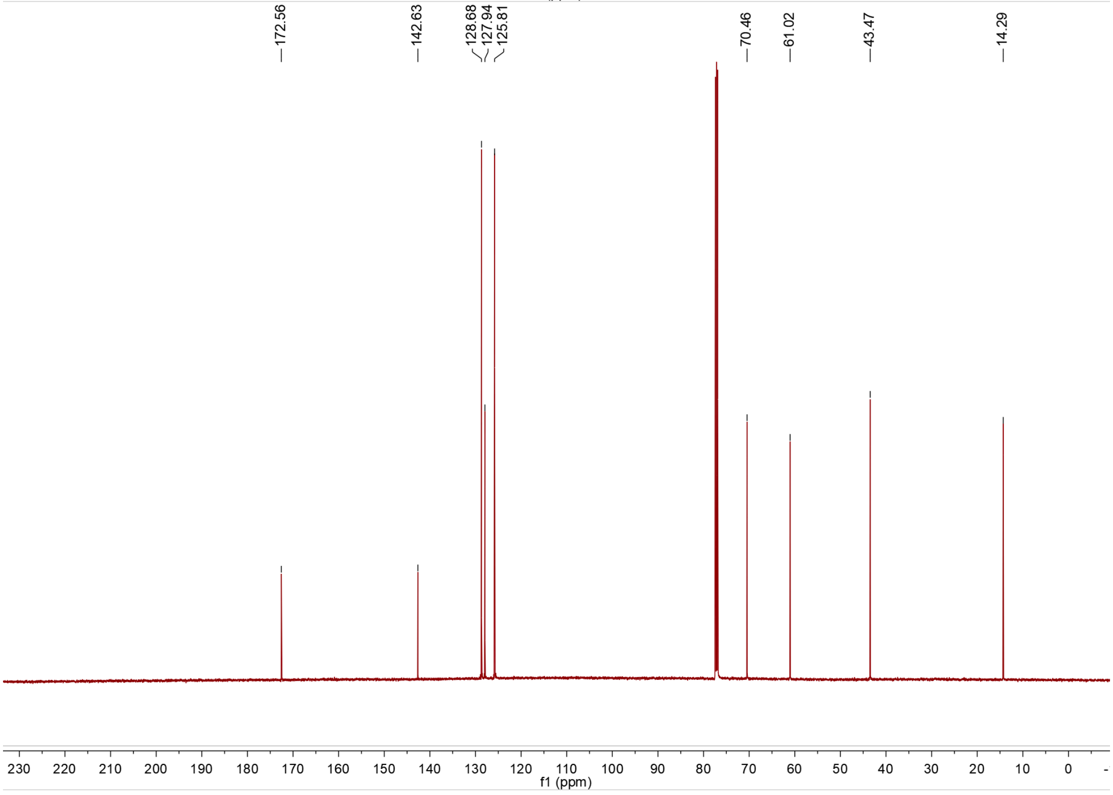

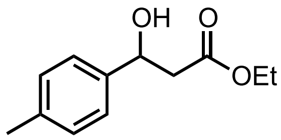
OH
O
OEt

4

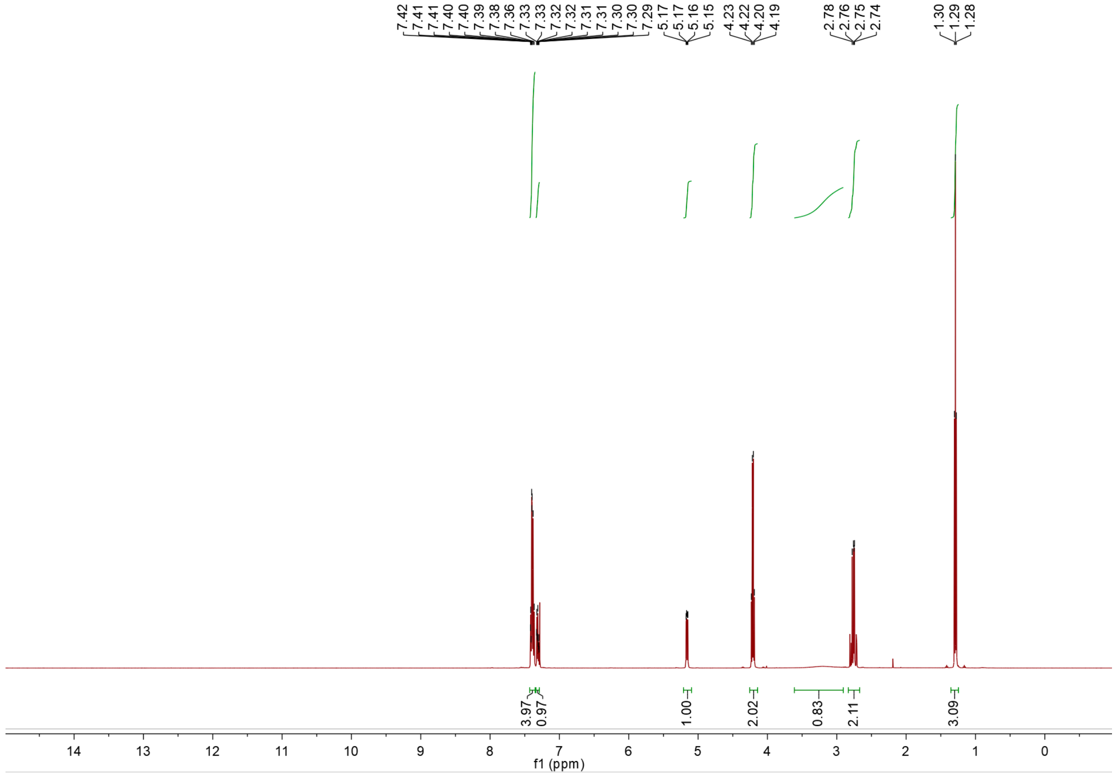
7.42 7.41 7.41 7.40 7.40 7.39 7.38 7.36 7.33 7.33 7.32 7.32 7.31 7.31 7.30 7.30 7.29
5.17 5.17 5.16 5.15
4.23 4.22 4.20 4.19
2.78 2.76 2.75 2.74
1.30 1.29 1.28
3.97 0.97 1.00 2.02 0.83 2.11 3.09
14 13 12 11 10 9 8 7 6 5 4 3 2 1 0
f1 (ppm)

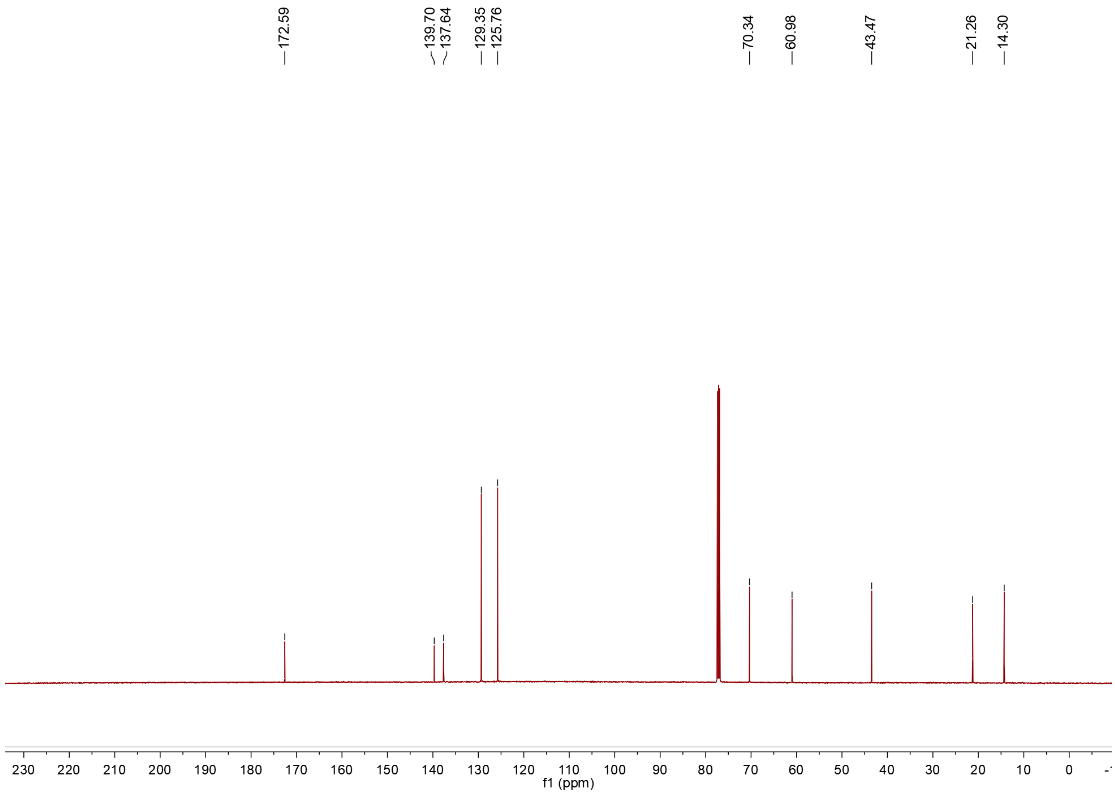
172.59
139.70 137.64
129.35 125.76
70.34
60.98
43.47
21.26 14.30
230 220 210 200 190 180 170 160 150 140 130 120 110 100 90 80 70 60 50 40 30 20 10 0
f1 (ppm)

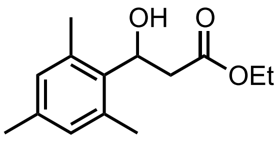
OH
O
OEt

5

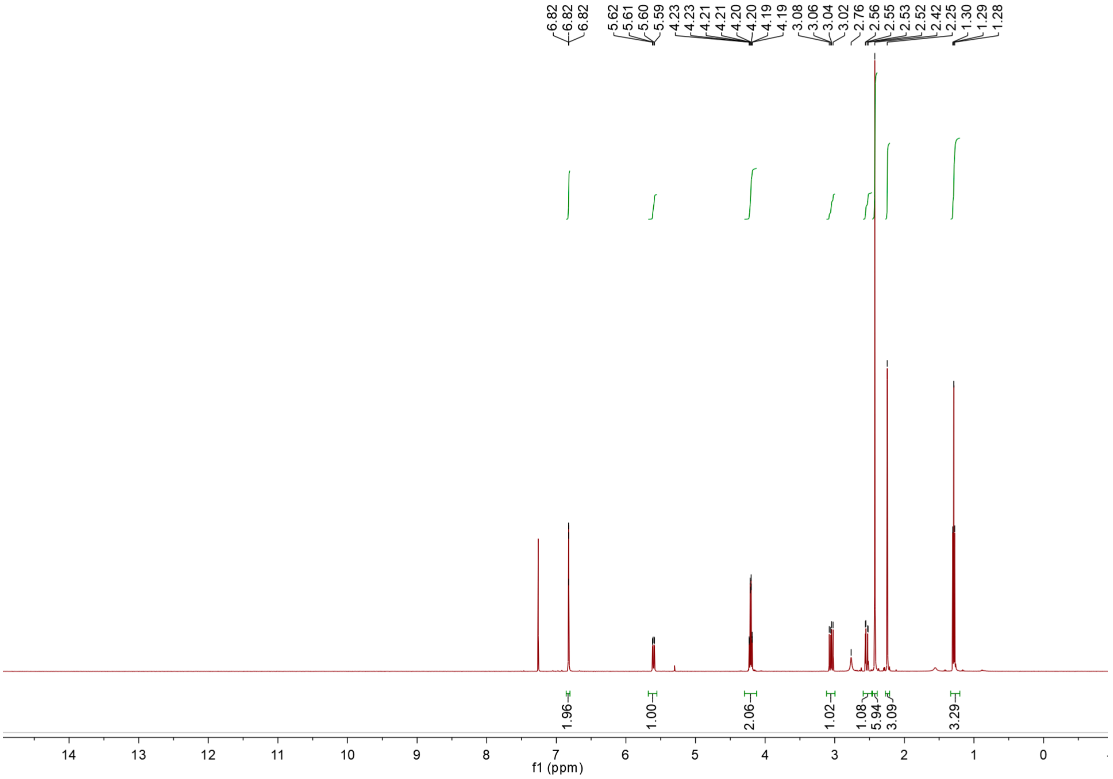
6.82
6.82
6.82
5.62
5.61
5.60
5.59
4.23
4.23
4.21
4.21
4.20
4.20
4.19
4.19
3.08
3.06
3.04
3.02
2.76
2.56
2.55
2.53
2.52
2.42
2.25
1.30
1.29
1.28
1.96
1.00
2.06
1.02
1.08
5.94
3.09
3.29
f1 (ppm)

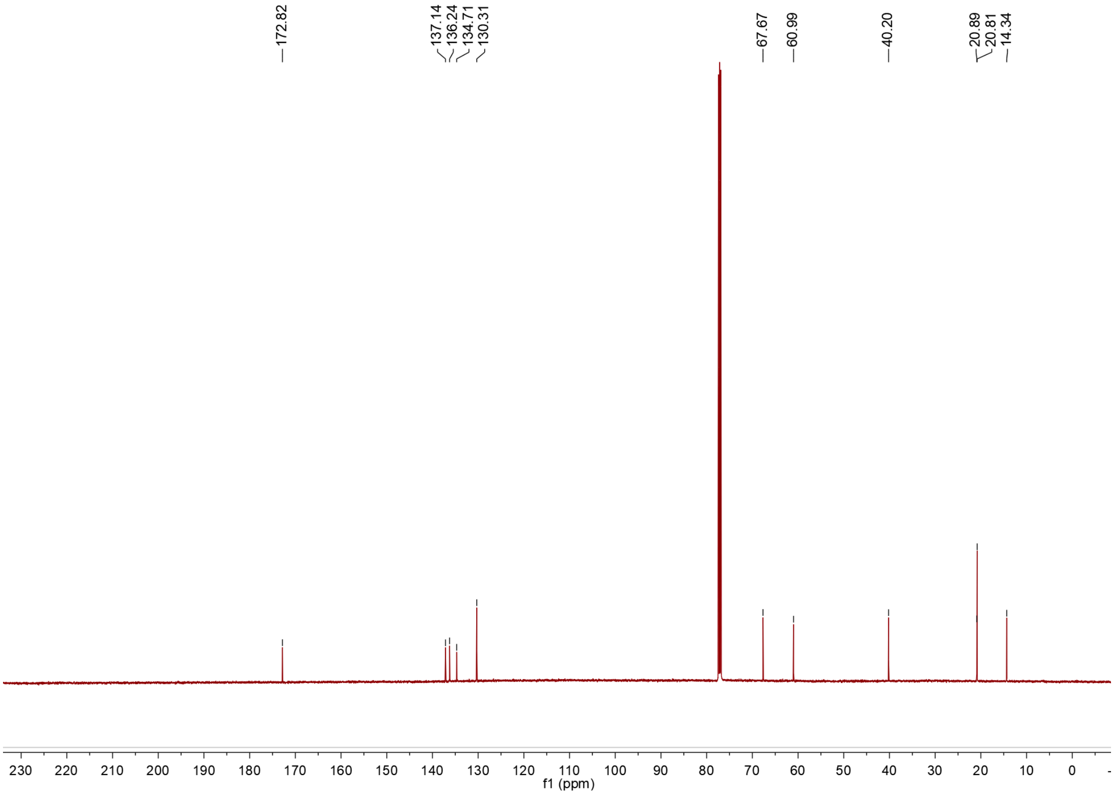
172.82
137.14
136.24
134.71
130.31
67.67
60.99
40.20
20.89
20.81
14.34
f1 (ppm)

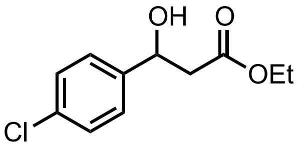
OH
O
OEt
Cl

6

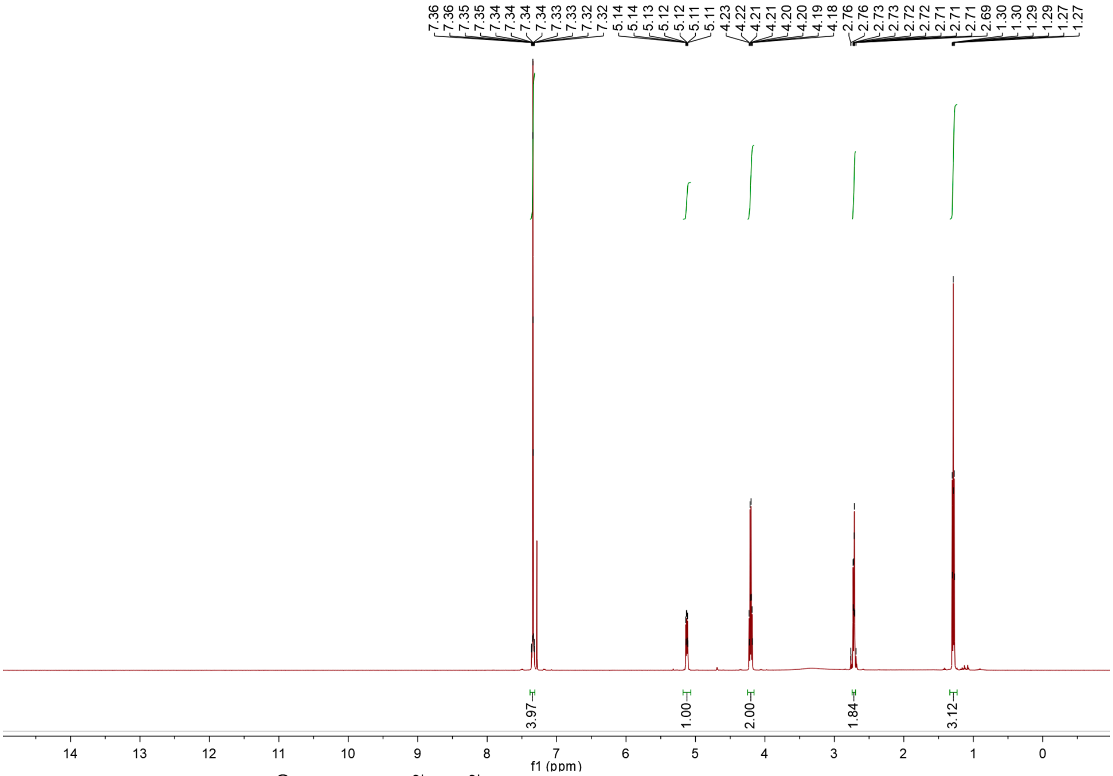
7.36
7.36
7.35
7.35
7.34
7.34
7.34
7.34
7.33
7.33
7.32
7.32
5.14
5.14
5.13
5.12
5.12
5.11
5.11
4.23
4.22
4.21
4.21
4.20
4.20
4.19
4.18
2.76
2.76
2.73
2.73
2.72
2.72
2.71
2.71
2.71
2.69
1.30
1.30
1.29
1.29
1.27
1.27
3.97
1.00
2.00
1.84
3.12
f1 (ppm)

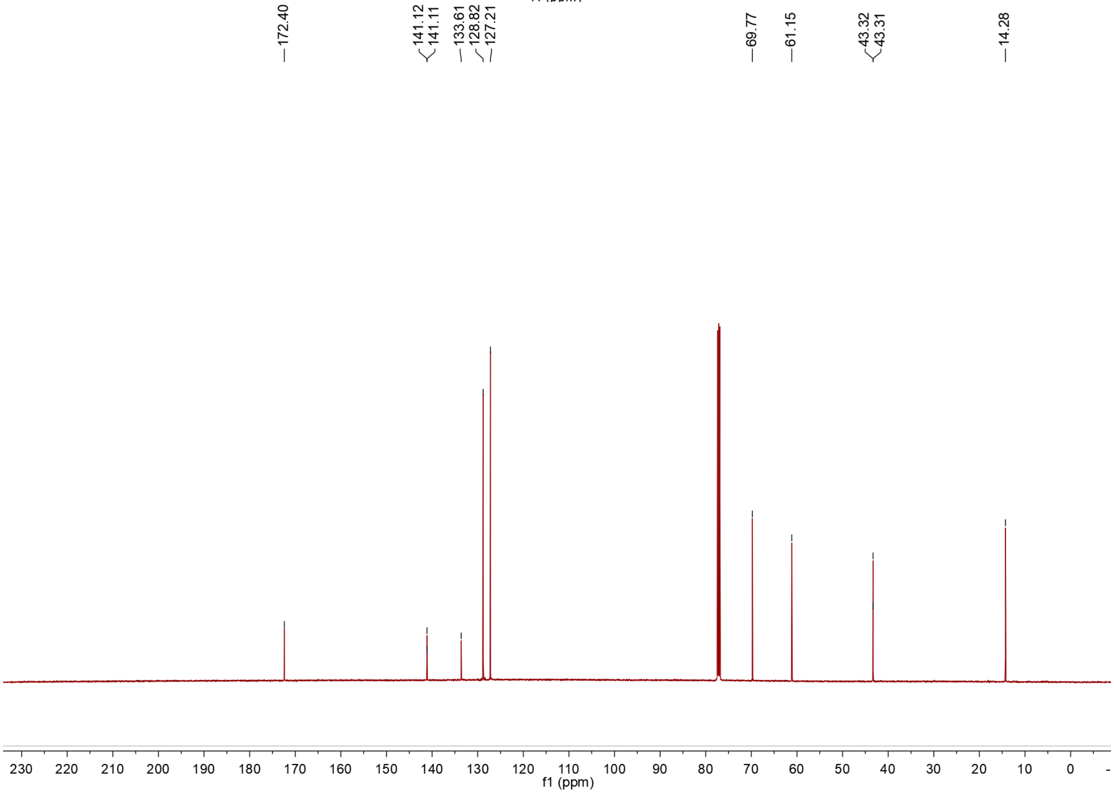
172.40
141.12
141.11
133.61
128.82
127.21
69.77
61.15
43.32
43.31
14.28
f1 (ppm)

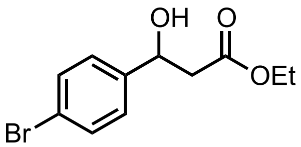

7

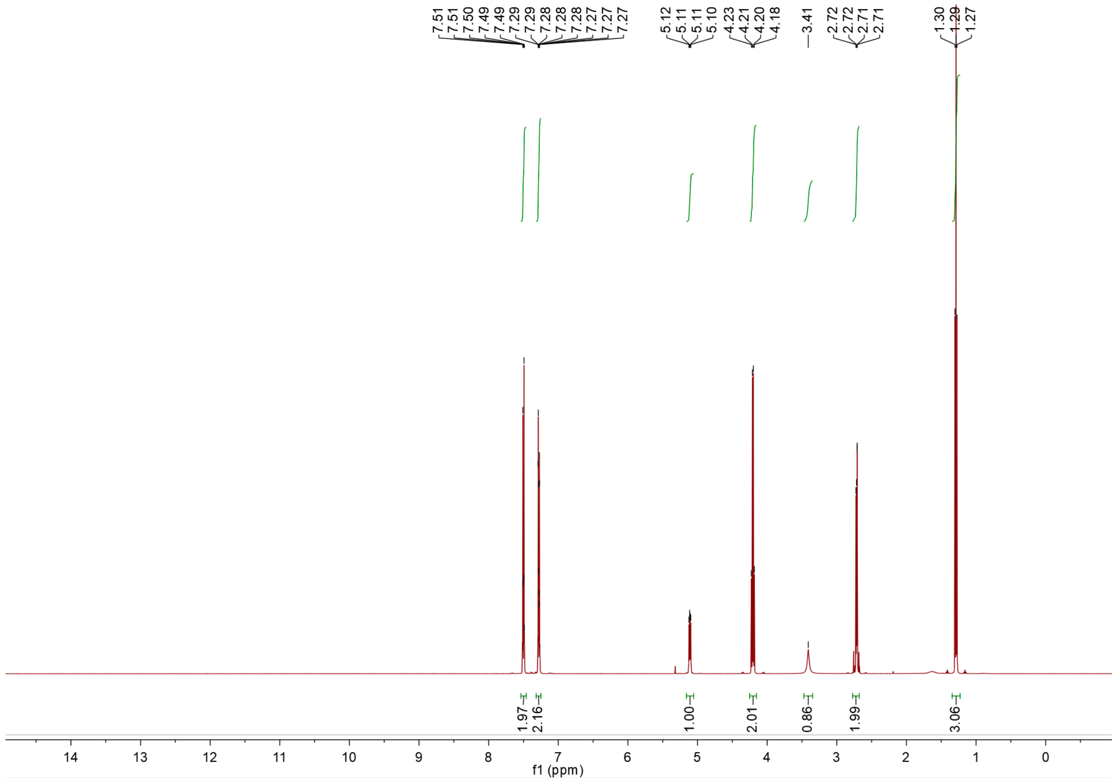

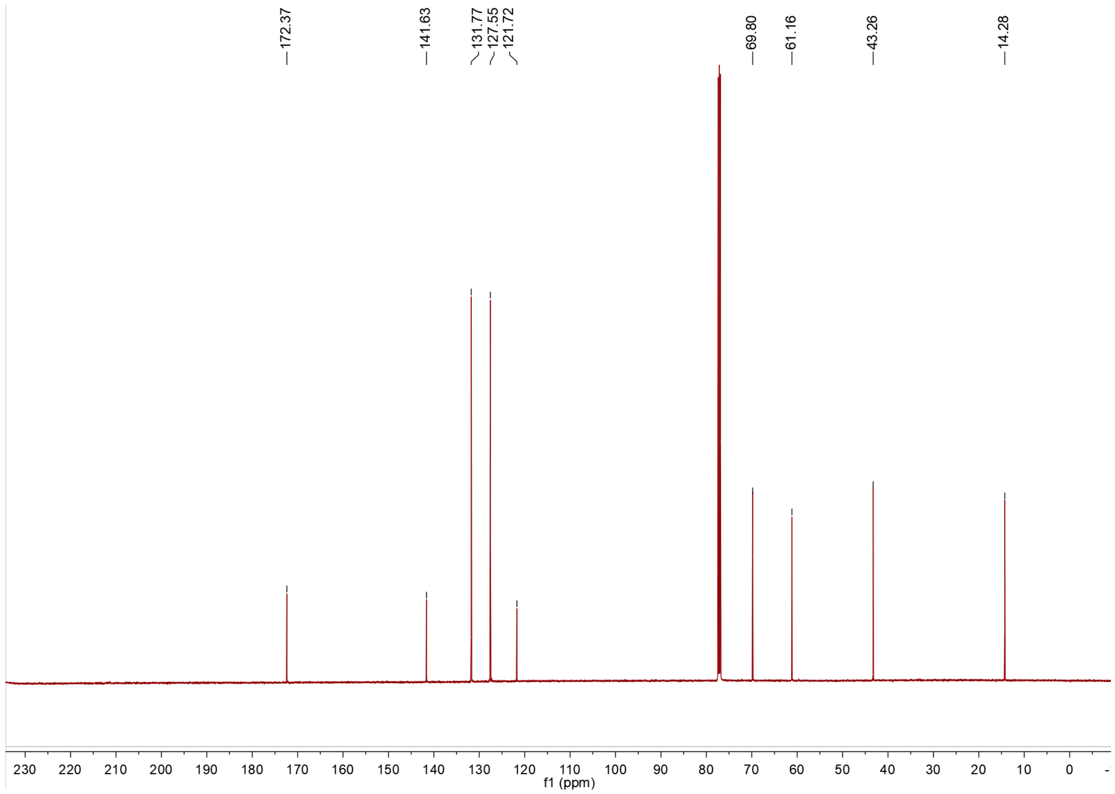

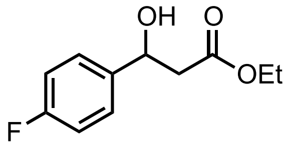

8

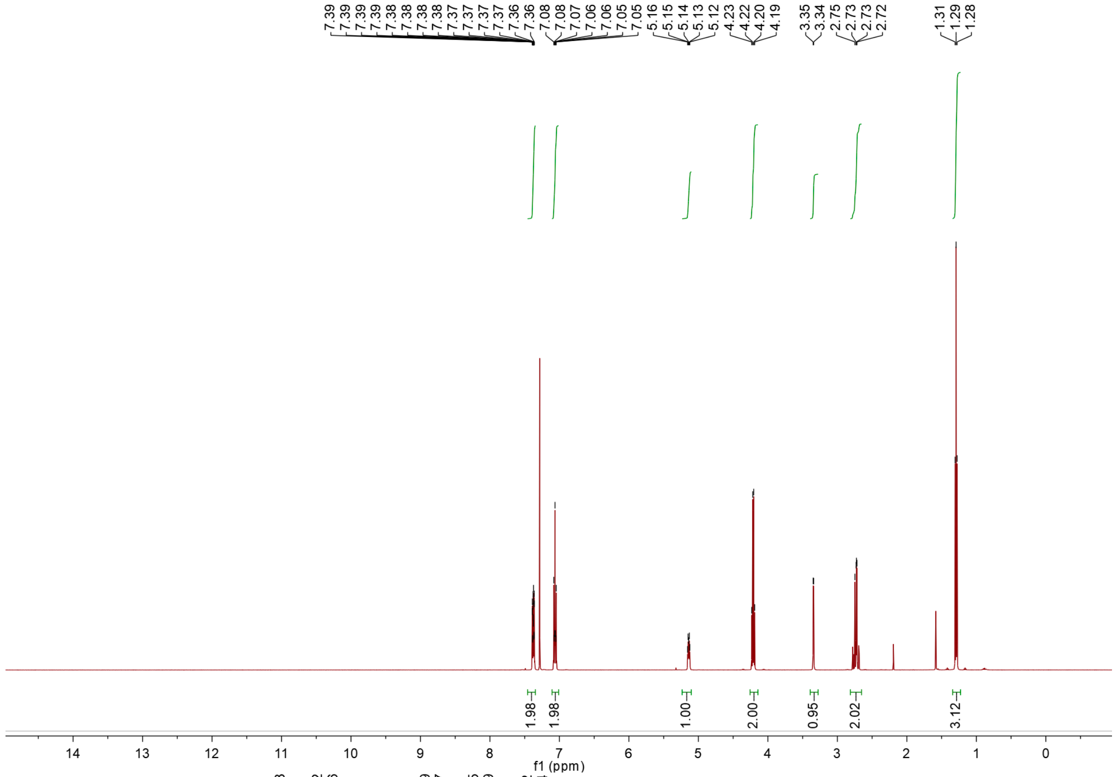

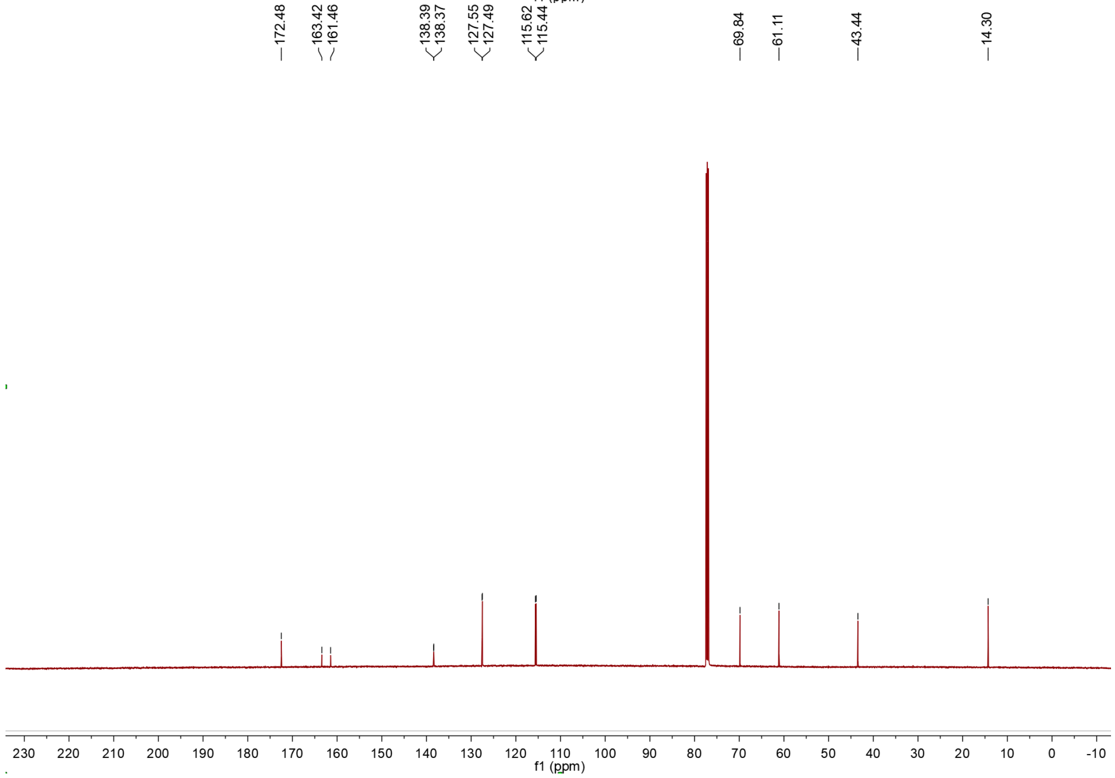

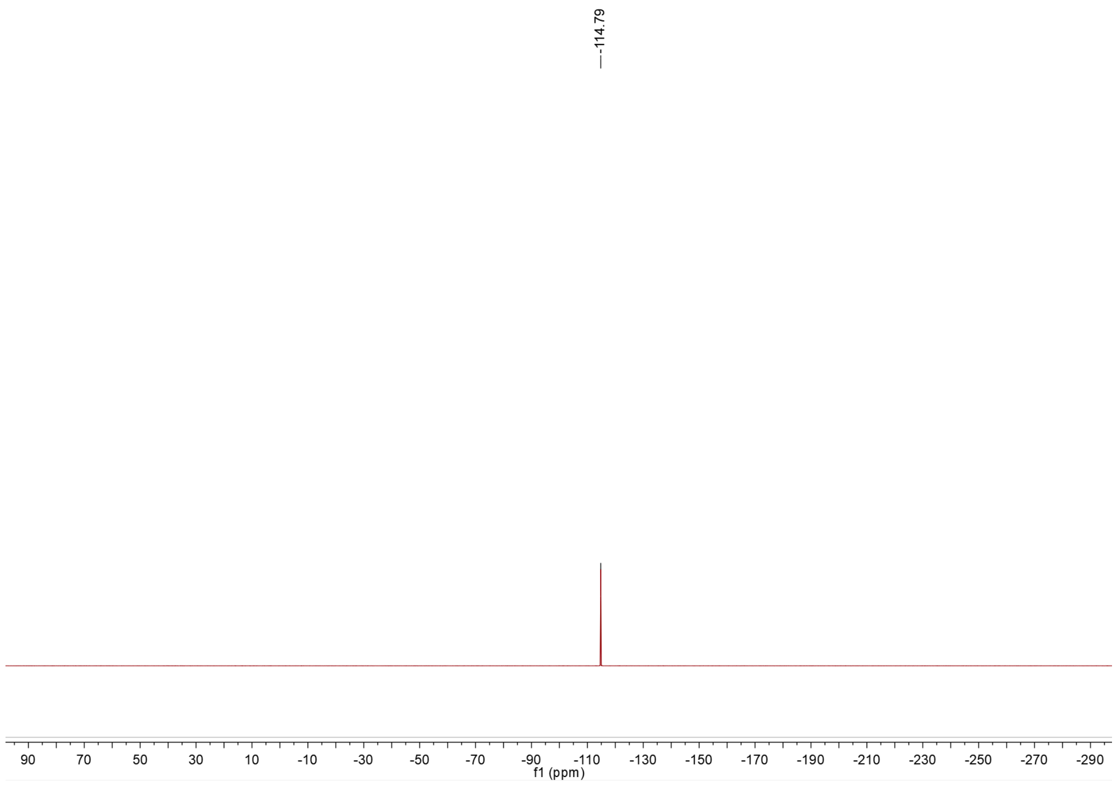
-114.79
90 70 50 30 10 -10 -30 -50 -70 -90 -110 -130 -150 -170 -190 -210 -230 -250 -270 -290
f1 (ppm)

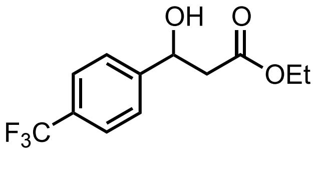
OH
O
OEt
F3C

9

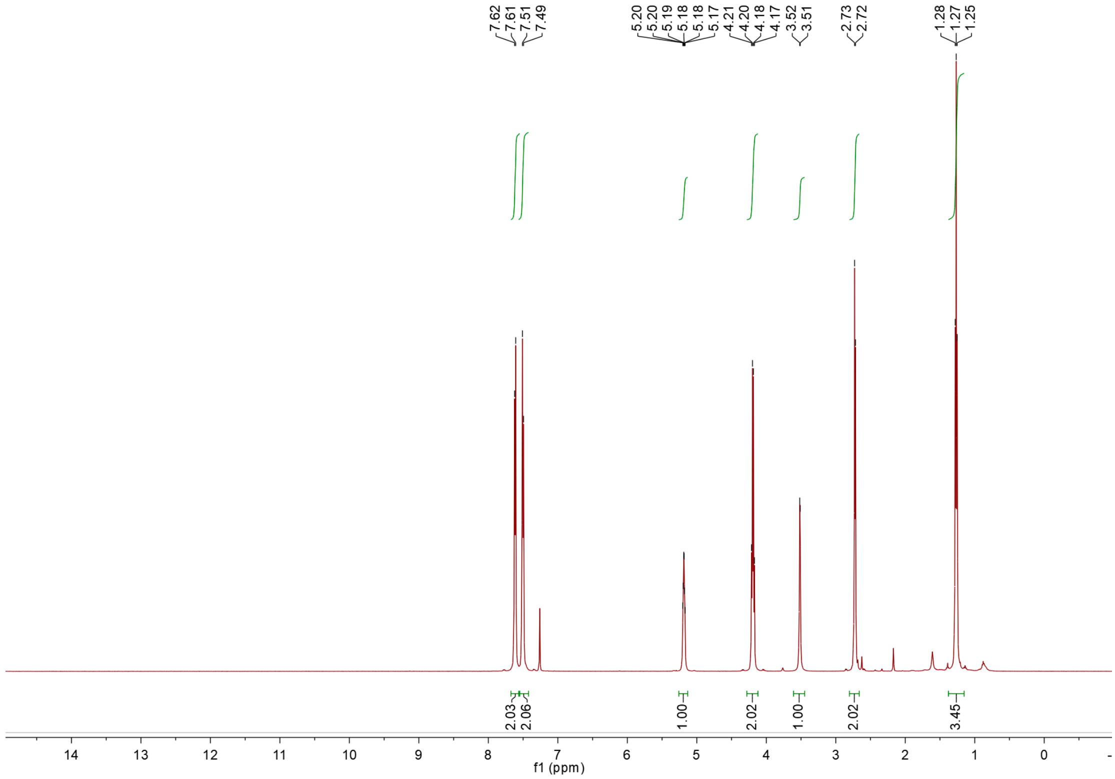
7.62
7.61
7.51
7.49
5.20
5.20
5.19
5.18
5.18
5.17
4.21
4.20
4.18
4.17
3.52
3.51
2.73
2.72
1.28
1.27
1.25
2.03
2.06
1.00
2.02
1.00
2.02
3.45
14
13
12
11
10
9
8
7
6
5
4
3
2
1
0
f1 (ppm)

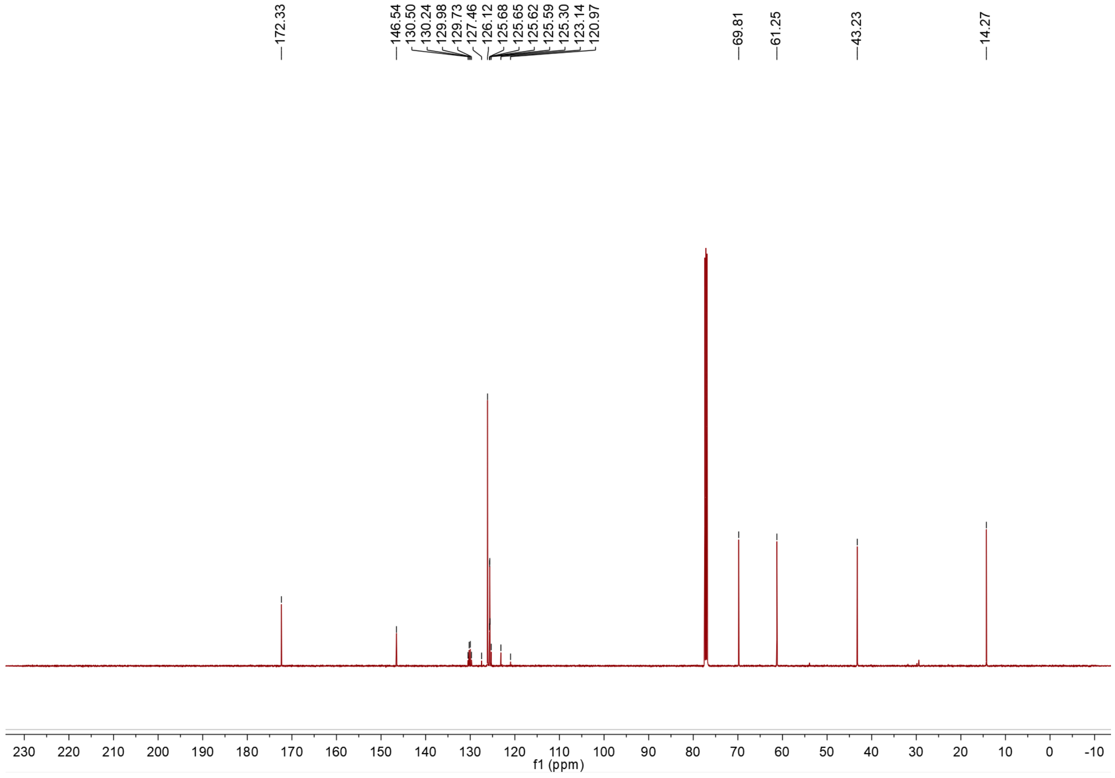
172.33
146.54
130.50
130.24
129.98
129.73
127.46
126.12
125.68
125.65
125.62
125.59
125.30
123.14
120.97
69.81
61.25
43.23
14.27
230
220
210
200
190
180
170
160
150
140
130
120
110
100
90
80
70
60
50
40
30
20
10
0
-10
f1 (ppm)

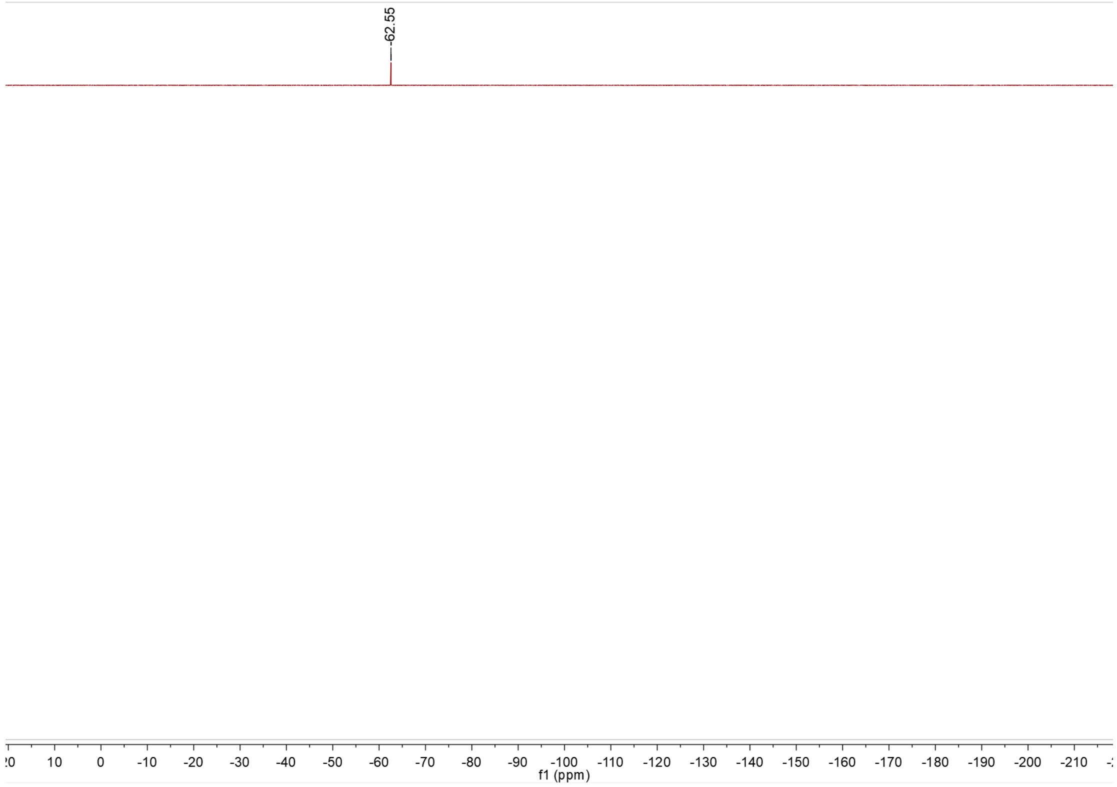
-62.55
20 10 0 -10 -20 -30 -40 -50 -60 -70 -80 -90 -100 -110 -120 -130 -140 -150 -160 -170 -180 -190 -200 -210
f1 (ppm)

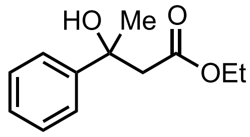
HO
Me
O
OEt

10

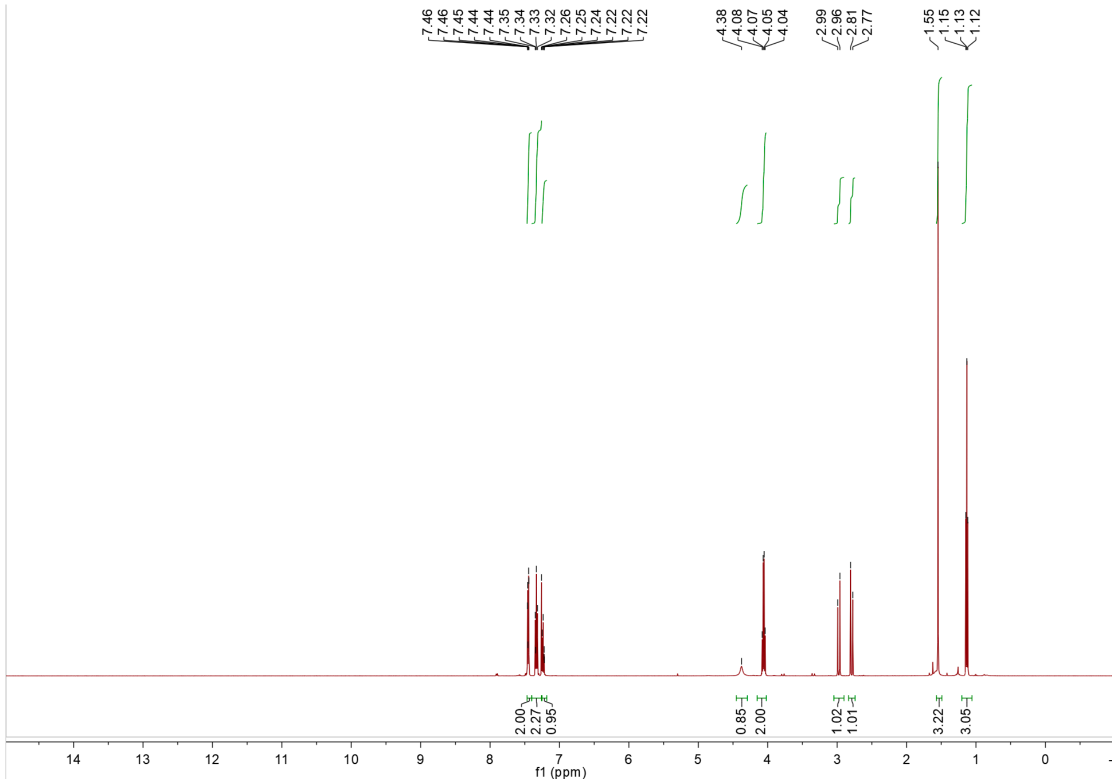
7.46
7.46
7.45
7.44
7.44
7.35
7.34
7.33
7.32
7.26
7.25
7.24
7.22
7.22
7.22
4.38
4.08
4.07
4.05
4.04
2.99
2.96
2.81
2.77
1.55
1.15
1.13
1.12
2.00
2.27
0.95
0.85
2.00
1.02
1.01
3.22
3.05
f1 (ppm)

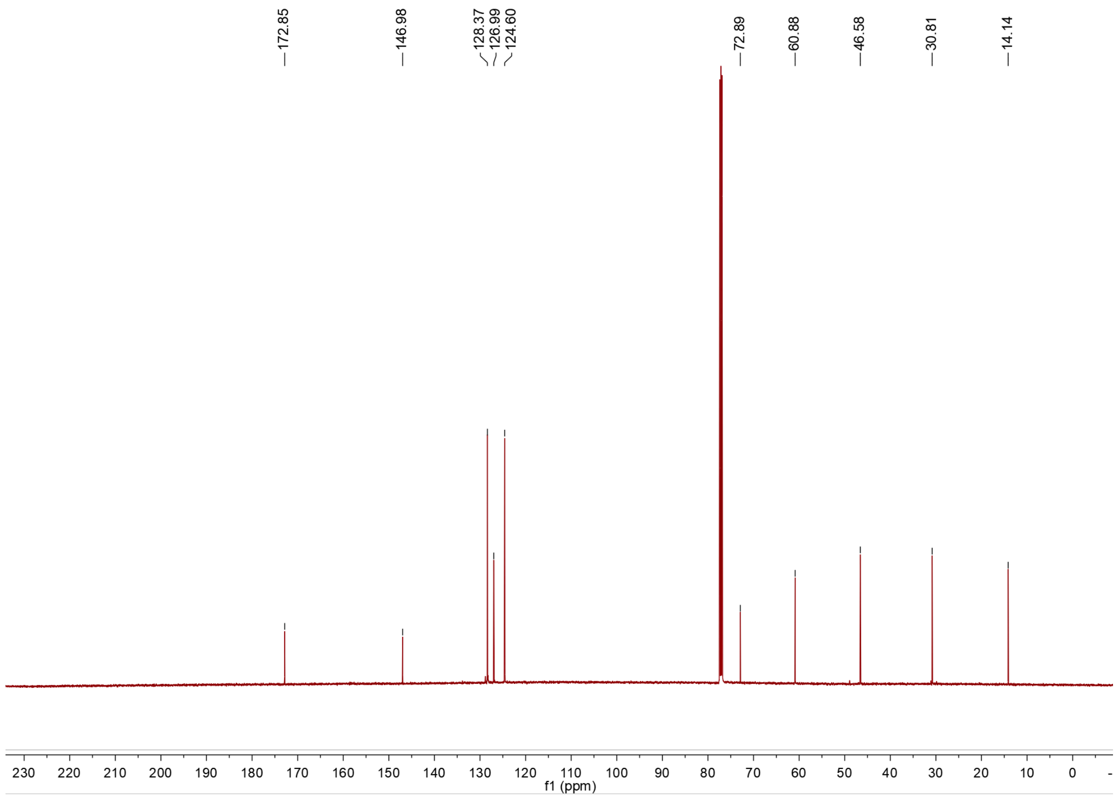
172.85
146.98
128.37
126.99
124.60
72.89
60.88
46.58
30.81
14.14
f1 (ppm)

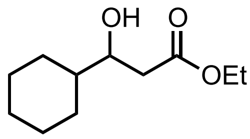

11

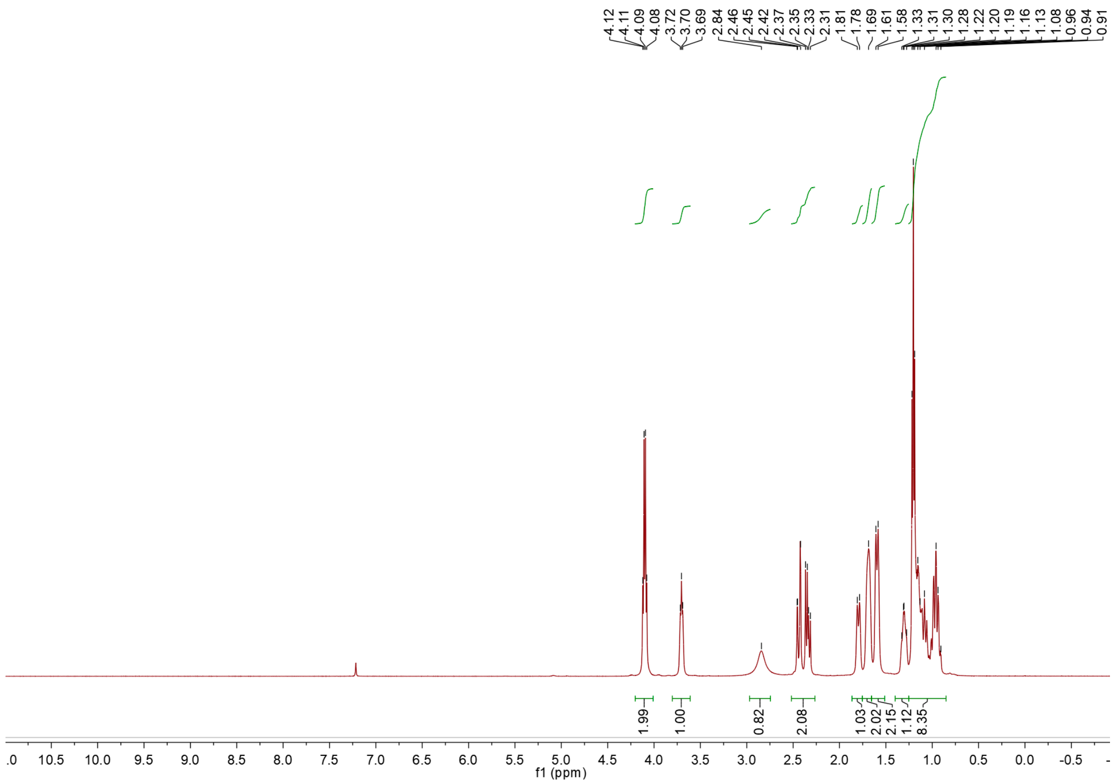

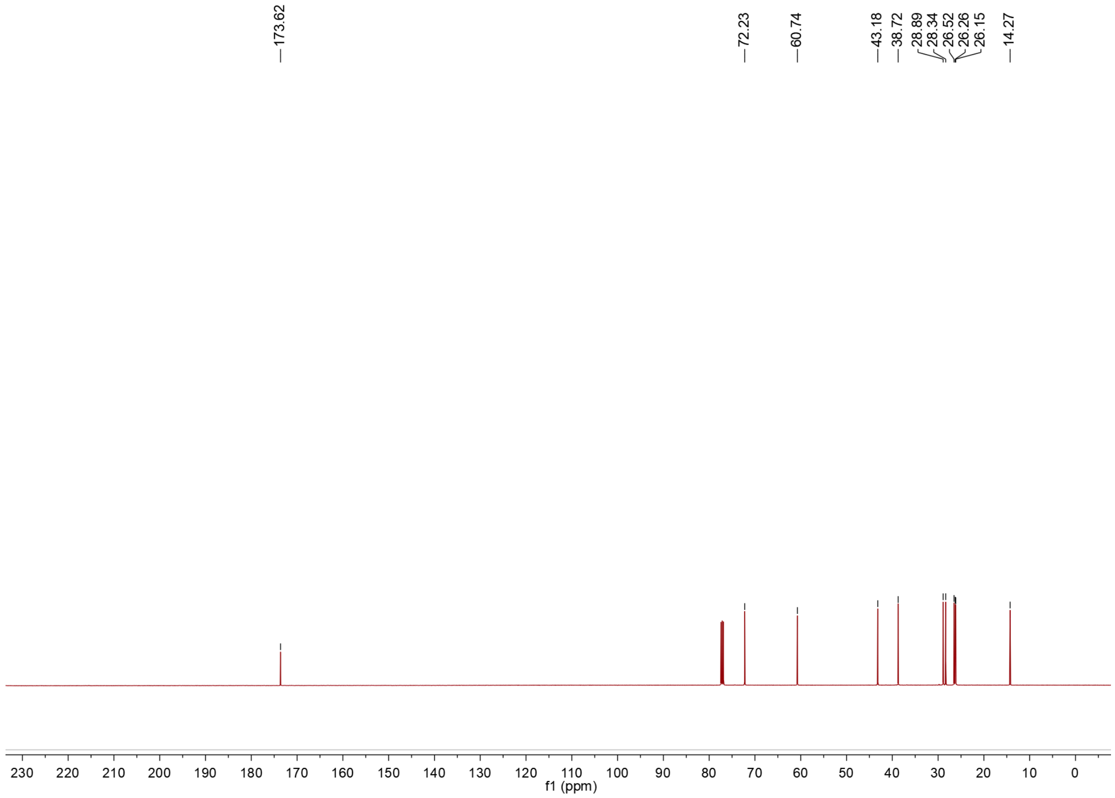

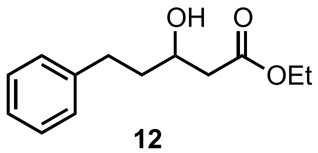
OH
O
OEt

12

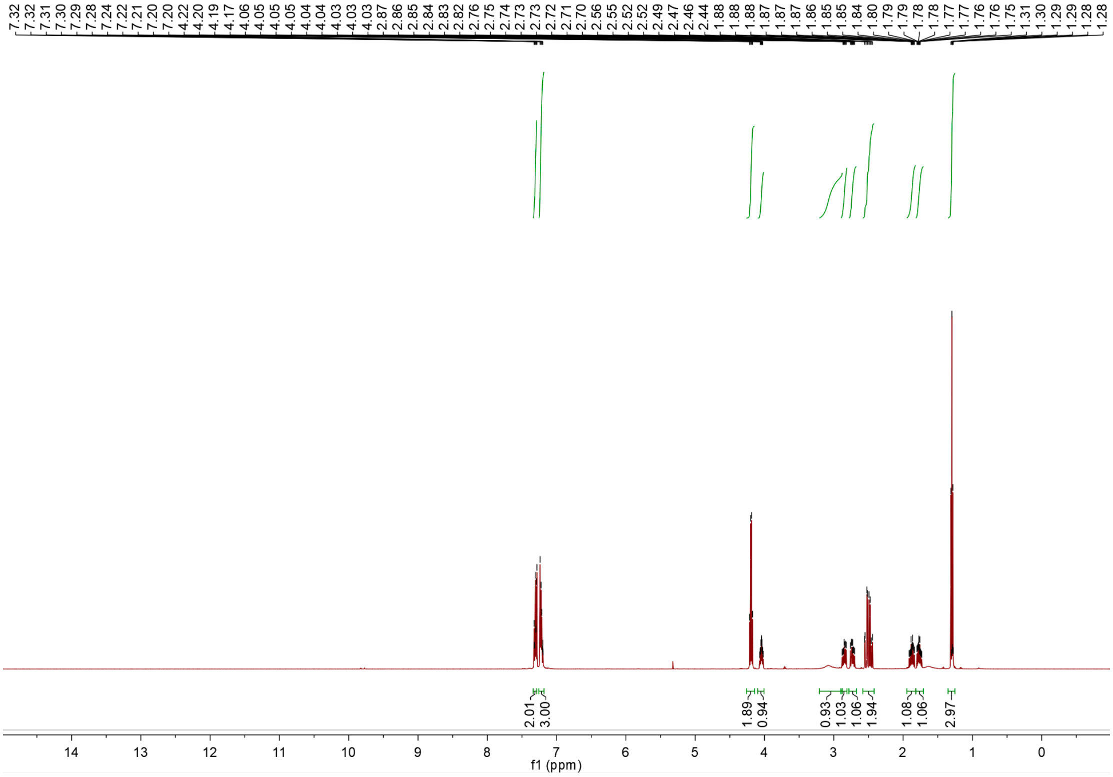
7.32 7.32 7.31 7.30 7.29 7.28 7.24 7.22 7.21 7.20 7.20 4.22 4.20 4.19 4.17 4.06 4.05 4.05 4.04 4.04 4.03 4.03 2.87 2.86 2.85 2.84 2.83 2.82 2.76 2.75 2.74 2.73 2.73 2.72 2.71 2.70 2.56 2.55 2.52 2.52 2.49 2.47 2.46 2.44 1.88 1.88 1.88 1.87 1.87 1.86 1.85 1.85 1.84 1.80 1.79 1.79 1.78 1.78 1.77 1.77 1.76 1.76 1.75 1.31 1.30 1.29 1.29 1.28 1.28
2.01 3.00 1.89 0.94 0.93 1.03 1.06 1.94 1.08 1.06 2.97
14 13 12 11 10 9 8 7 6 5 4 3 2 1 0
f1 (ppm)

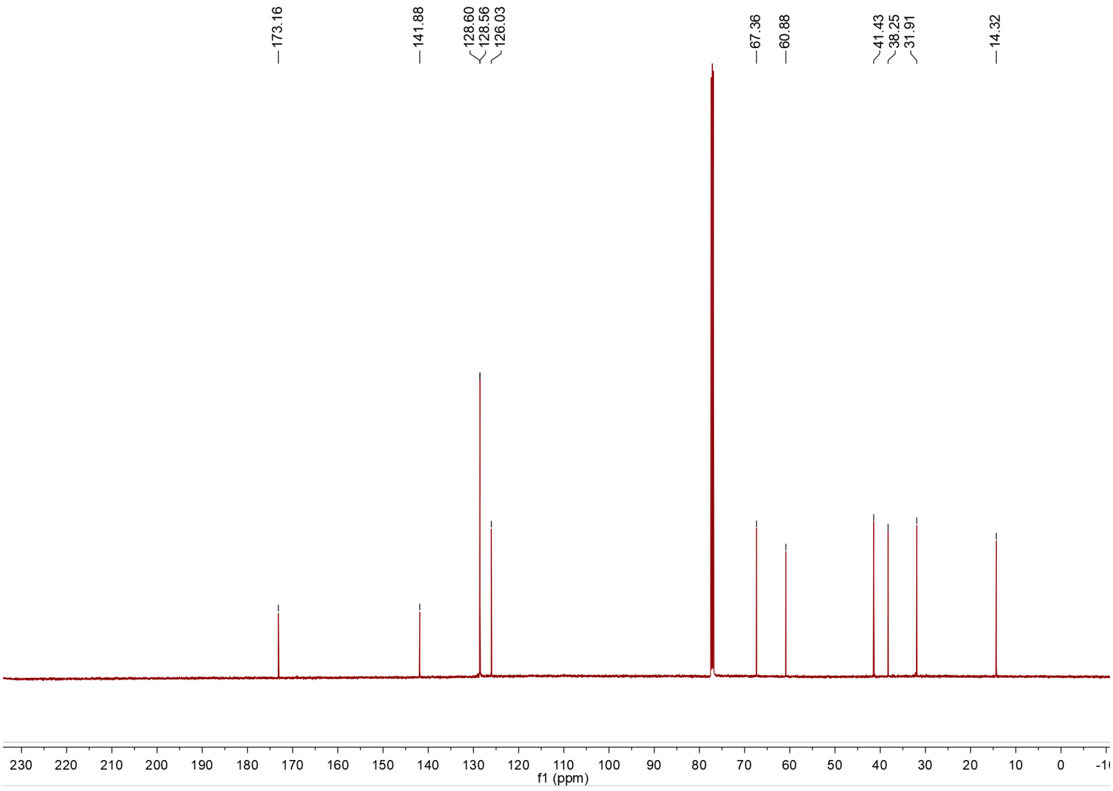
173.16
141.88
128.60 128.56 126.03
67.36
60.88
41.43 38.25 31.91
14.32
230 220 210 200 190 180 170 160 150 140 130 120 110 100 90 80 70 60 50 40 30 20 10 0
f1 (ppm)

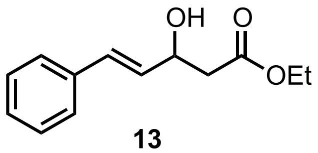

13

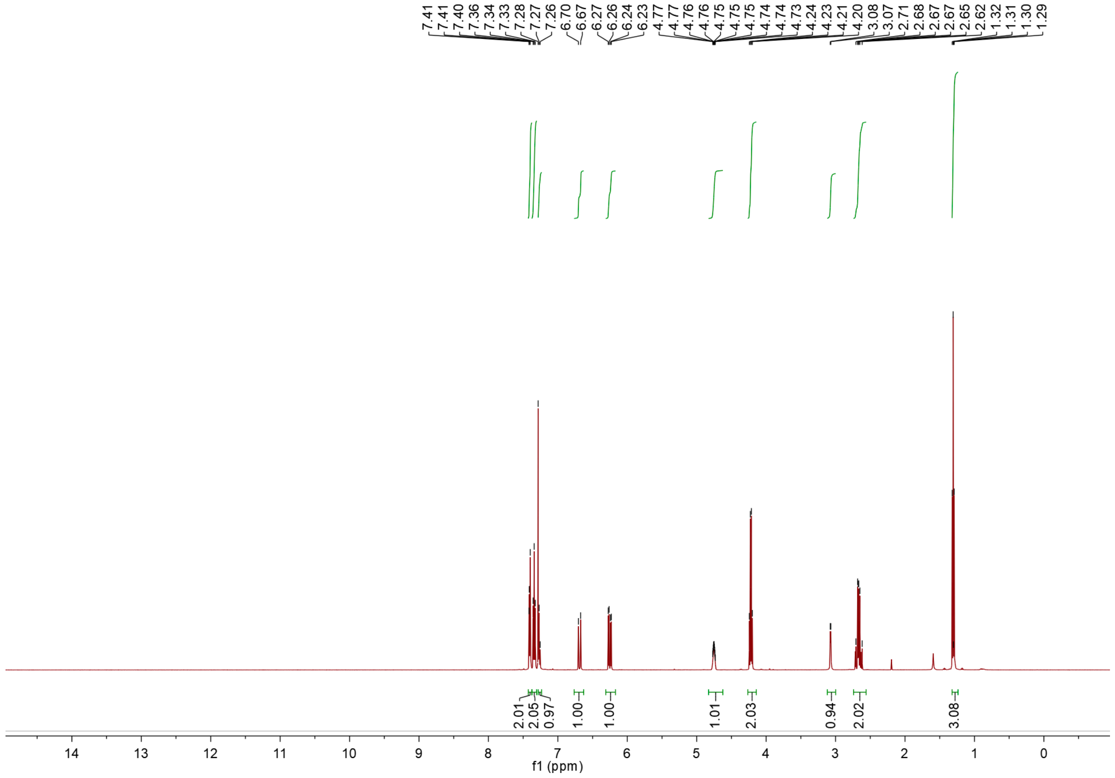

7.41
7.41
7.40
7.36
7.34
7.33
7.28
7.27
7.26
6.70
6.67
6.27
6.26
6.24
6.23
4.77
4.76
4.76
4.75
4.75
4.75
4.74
4.74
4.73
4.24
4.23
4.21
4.20
3.08
3.07
2.71
2.68
2.67
2.65
2.62
1.32
1.31
1.30
1.29
2.01
2.05
0.97
1.00
1.00
1.01
2.03
0.94
2.02
3.08
14
13
12
11
10
9
8
7
6
5
4
3
2
1
0
f1 (ppm)

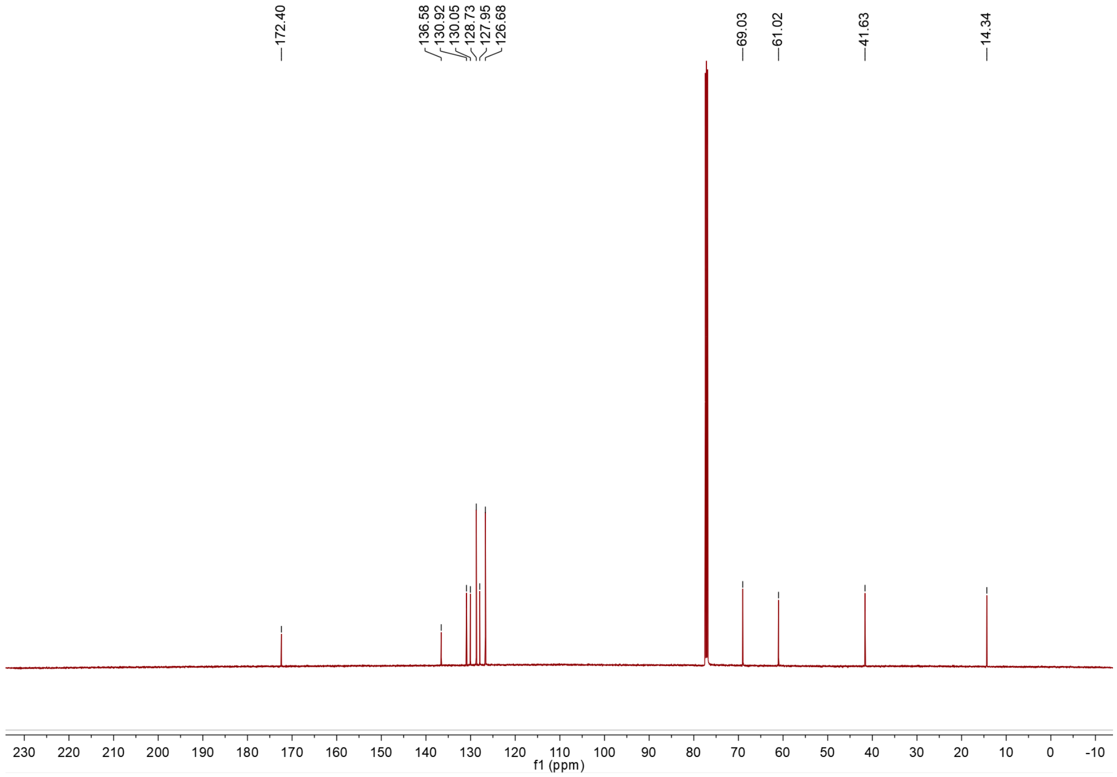

172.40
136.58
130.92
130.05
128.73
127.95
126.68
69.03
61.02
41.63
14.34
230
220
210
200
190
180
170
160
150
140
130
120
110
100
90
80
70
60
50
40
30
20
10
0
-10
f1 (ppm)

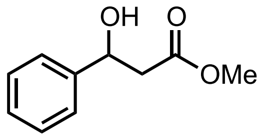

14

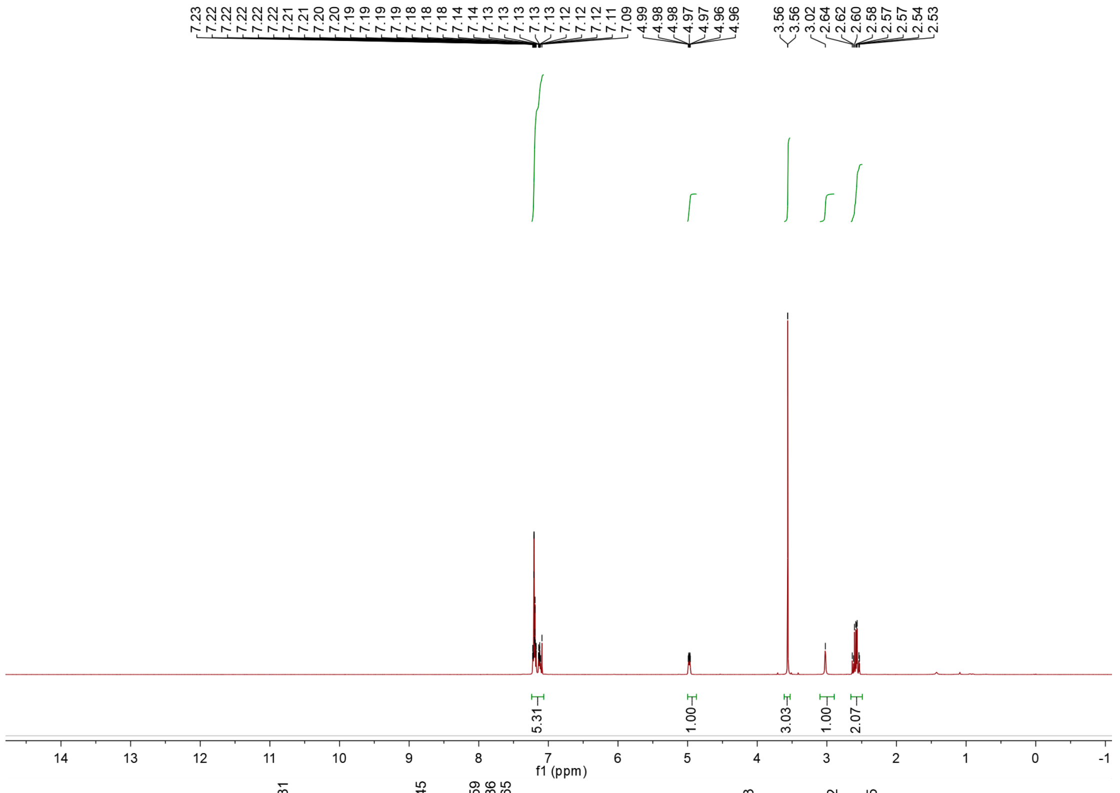
7.23
7.22
7.22
7.22
7.22
7.22
7.21
7.21
7.20
7.20
7.19
7.19
7.19
7.19
7.18
7.18
7.18
7.14
7.14
7.13
7.13
7.13
7.13
7.13
7.12
7.12
7.12
7.11
7.09
4.99
4.98
4.98
4.97
4.97
4.96
4.96
3.56
3.56
3.02
2.64
2.62
2.60
2.58
2.57
2.57
2.54
2.53
5.31
1.00
3.03
1.00
2.07
14
13
12
11
10
9
8
7
6
5
4
3
2
1
0
-1
f1 (ppm)

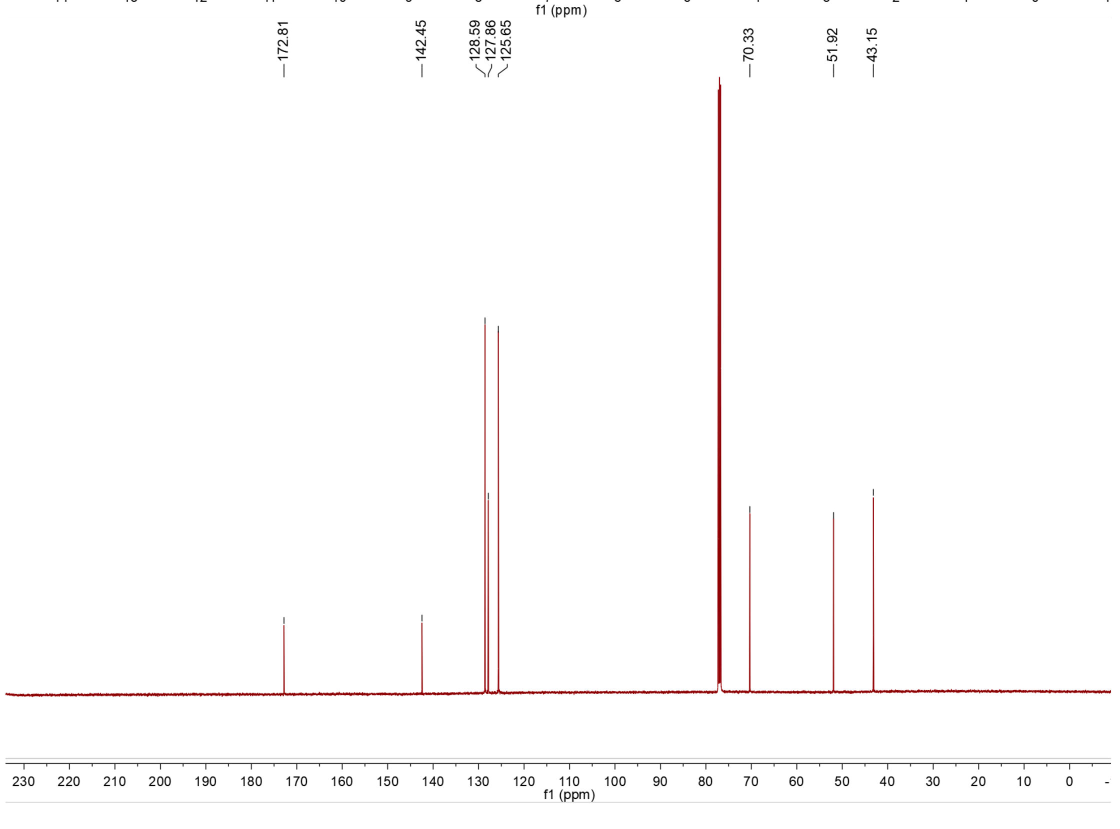
172.81
142.45
128.59
127.86
125.65
70.33
51.92
43.15
230
220
210
200
190
180
170
160
150
140
130
120
110
100
90
80
70
60
50
40
30
20
10
0
f1 (ppm)

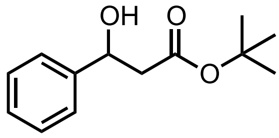
OH
O
O

15

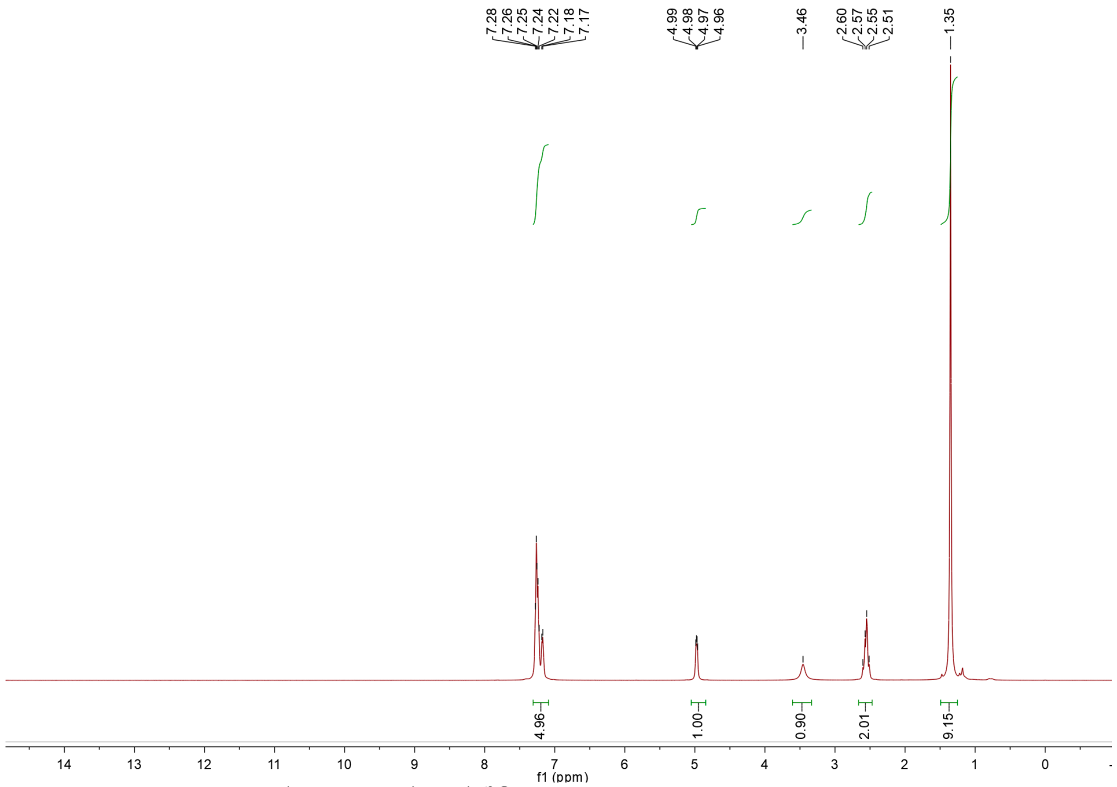
7.28
7.26
7.25
7.24
7.22
7.18
7.17
4.99
4.98
4.97
4.96
3.46
2.60
2.57
2.55
2.51
1.35
4.96
1.00
0.90
2.01
9.15
f1 (ppm)

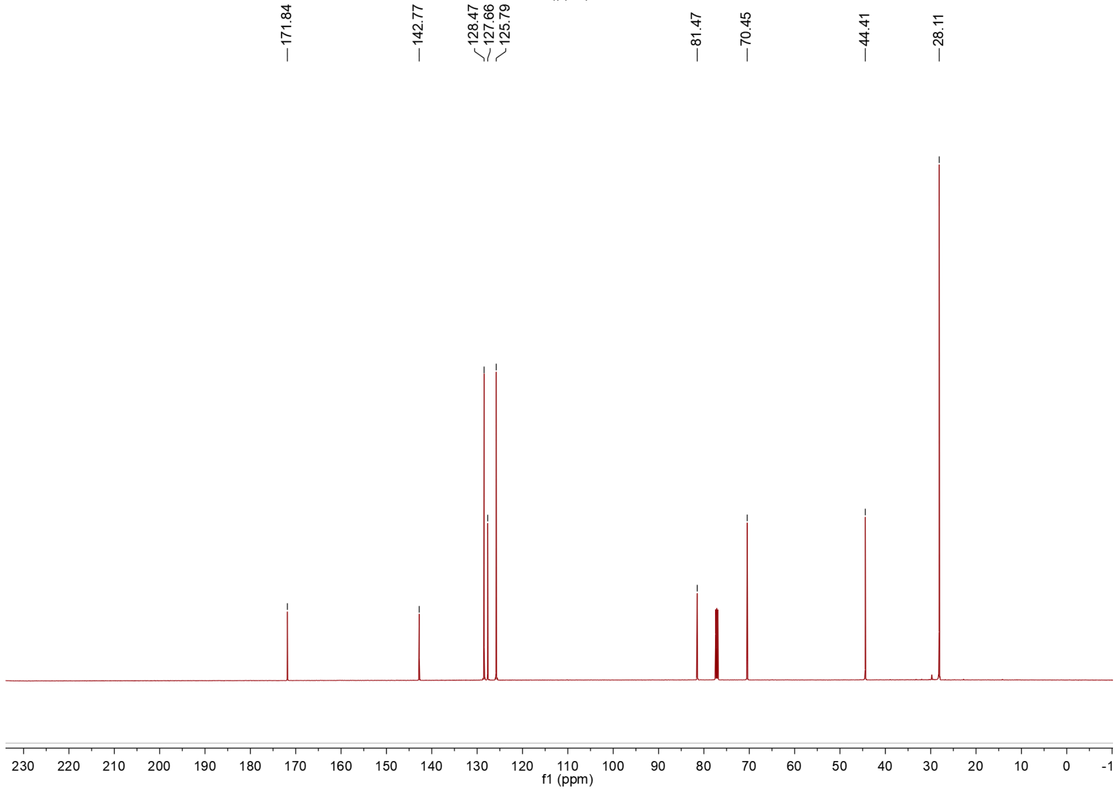
171.84
142.77
128.47
127.66
125.79
81.47
70.45
44.41
28.11
f1 (ppm)

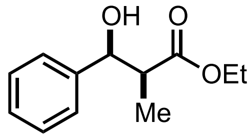
OH
O
OEt
Me

16

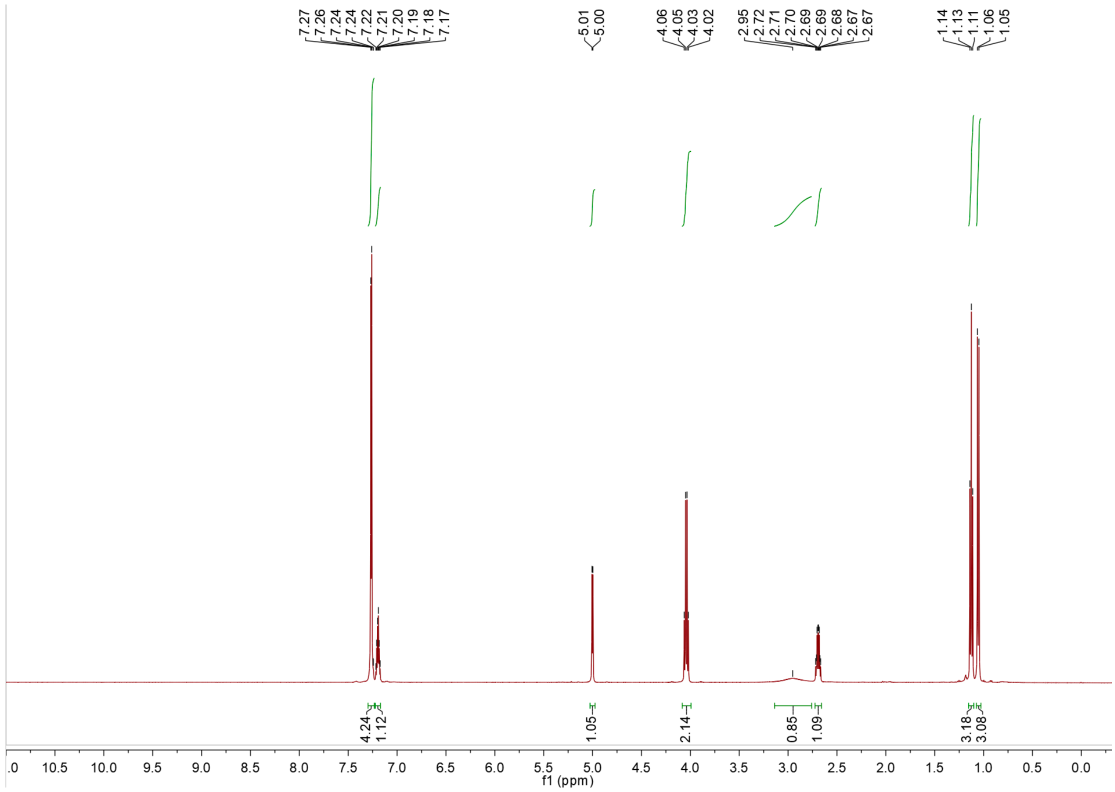
7.27
7.26
7.24
7.24
7.22
7.22
7.21
7.20
7.19
7.18
7.17
5.01
5.00
4.06
4.05
4.03
4.02
2.95
2.72
2.71
2.70
2.69
2.69
2.68
2.67
2.67
1.14
1.13
1.11
1.06
1.05
4.24
1.12
1.05
2.14
0.85
1.09
3.18
3.08
f1 (ppm)

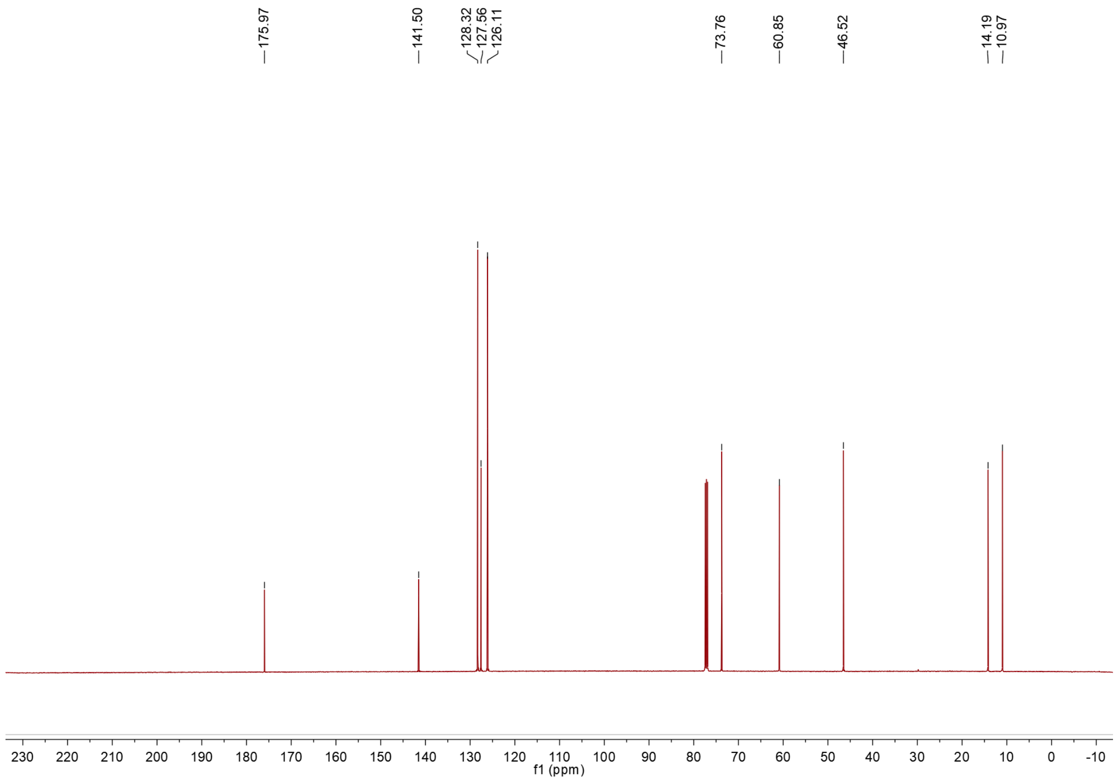
175.97
141.50
128.32
127.56
126.11
73.76
60.85
46.52
14.19
10.97
f1 (ppm)

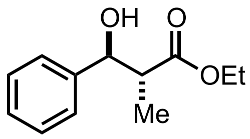
OH
O
OEt
Me

16

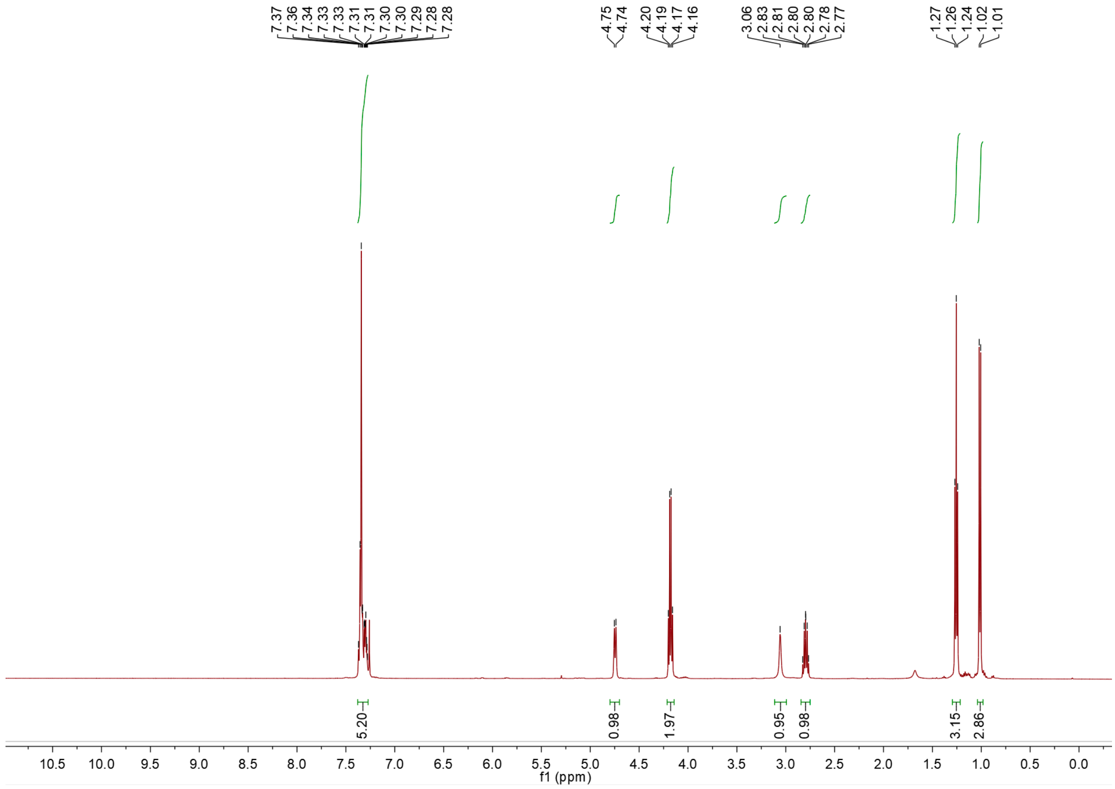
7.37
7.36
7.34
7.33
7.33
7.31
7.31
7.30
7.30
7.29
7.28
7.28
4.75
4.74
4.20
4.19
4.17
4.16
3.06
2.83
2.81
2.80
2.80
2.78
2.77
1.27
1.26
1.24
1.02
1.01
5.20
0.98
1.97
0.95
0.98
3.15
2.86
f1 (ppm)

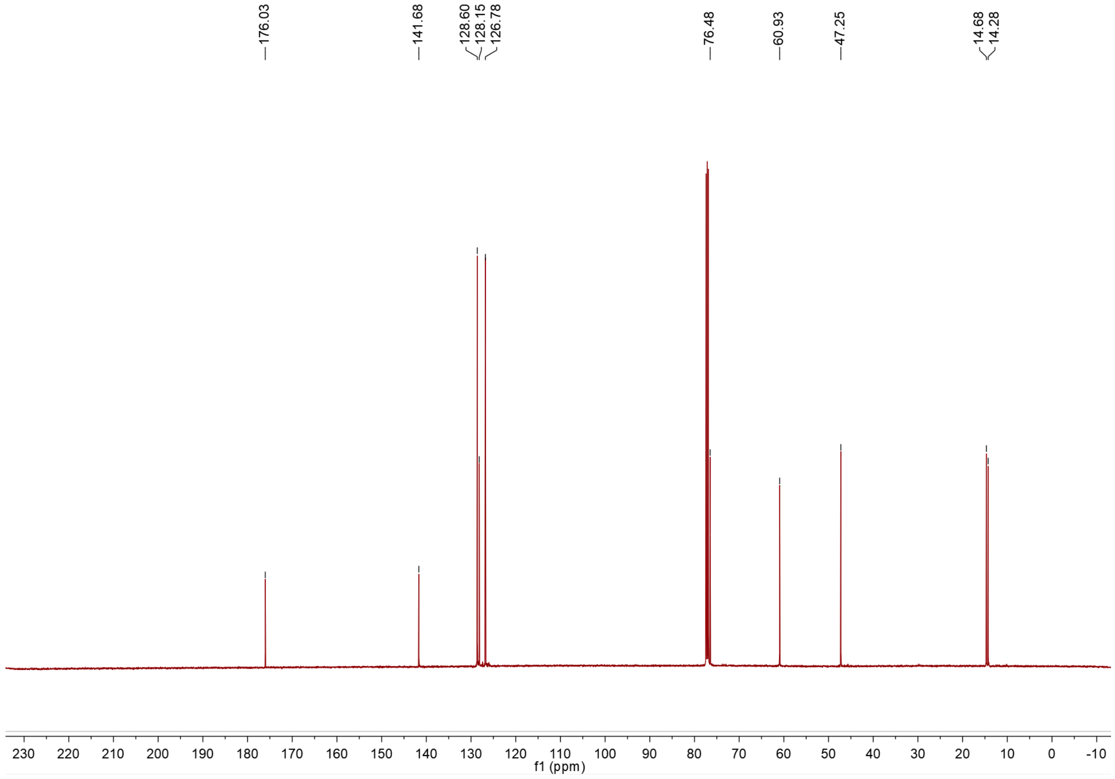
176.03
141.68
128.60
128.15
126.78
76.48
60.93
47.25
14.68
14.28
f1 (ppm)

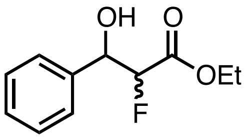
OH
O
OEt
F

17

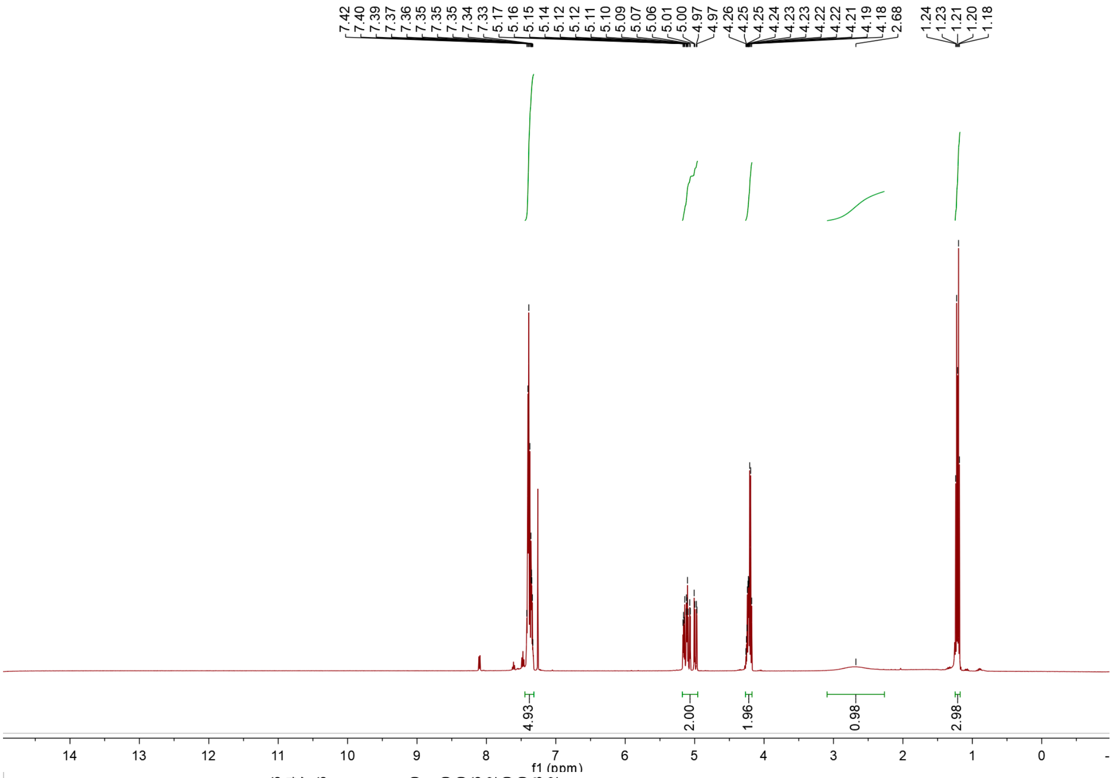
7.42
7.40
7.39
7.37
7.36
7.35
7.35
7.35
7.34
7.33
5.17
5.16
5.15
5.14
5.12
5.12
5.11
5.10
5.09
5.07
5.06
5.01
5.00
4.97
4.97
4.26
4.25
4.25
4.24
4.23
4.23
4.22
4.22
4.21
4.19
4.18
2.68
1.24
1.23
1.21
1.20
1.18
4.93
2.00
1.96
0.98
2.98
14
13
12
11
10
9
8
7
6
5
4
3
2
1
0
f1 (ppm)

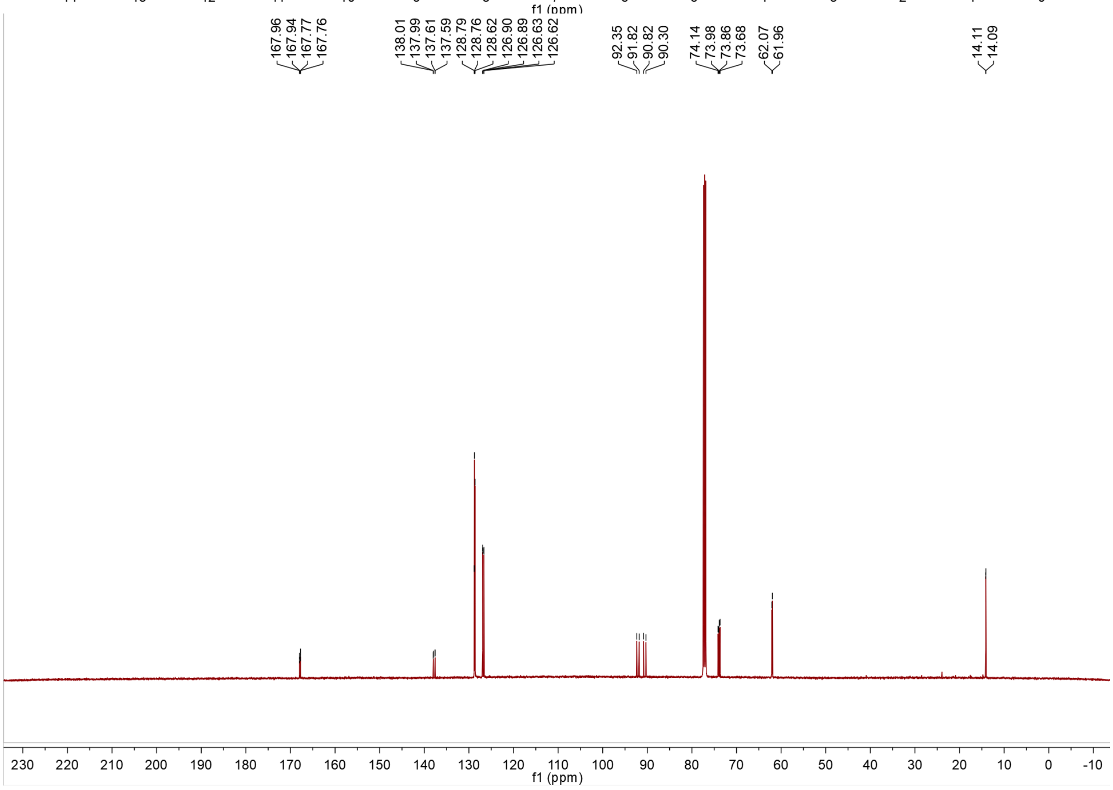
167.96
167.94
167.77
167.76
138.01
137.99
137.61
137.59
128.79
128.76
128.62
126.90
126.89
126.63
126.62
92.35
91.82
90.82
90.30
74.14
73.98
73.86
73.68
62.07
61.96
14.11
14.09
230
220
210
200
190
180
170
160
150
140
130
120
110
100
90
80
70
60
50
40
30
20
10
0
-10
f1 (ppm)

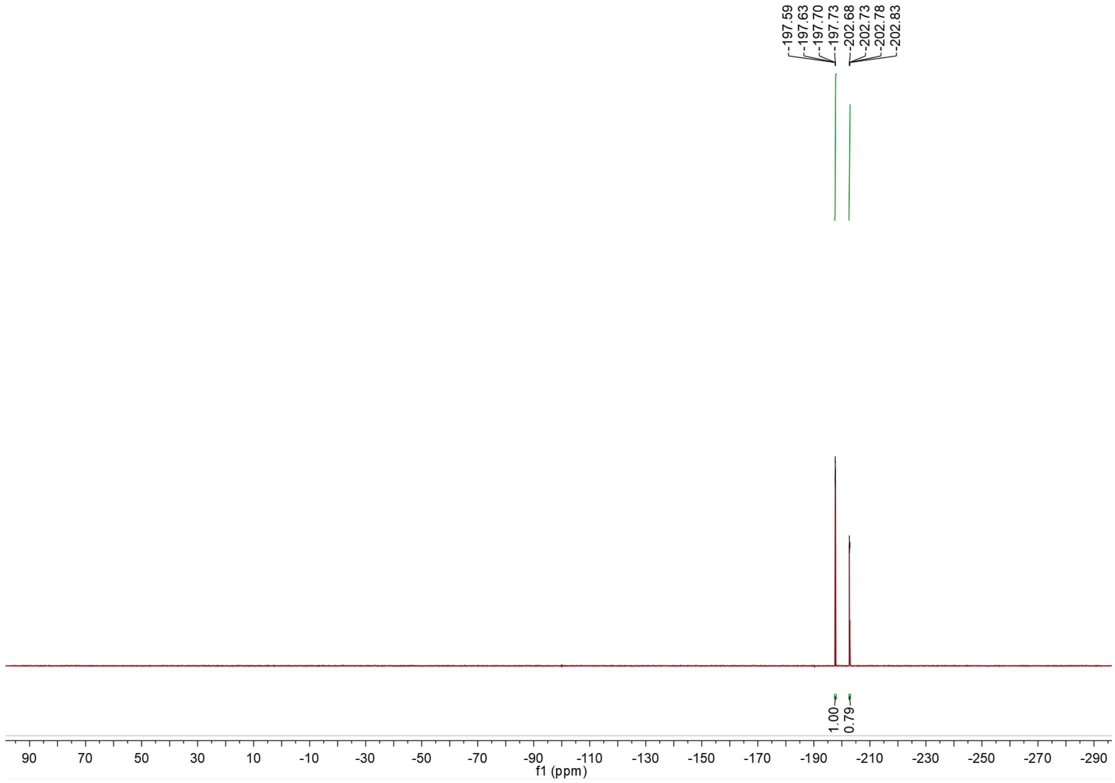

-197.59
-197.63
-197.70
-197.73
-202.68
-202.73
-202.78
-202.83
1.00
0.79
90 70 50 30 10 -10 -30 -50 -70 -90 -110 -130 -150 -170 -190 -210 -230 -250 -270 -290
f1 (ppm)

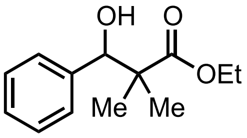
OH
O
OEt
Me
Me

18

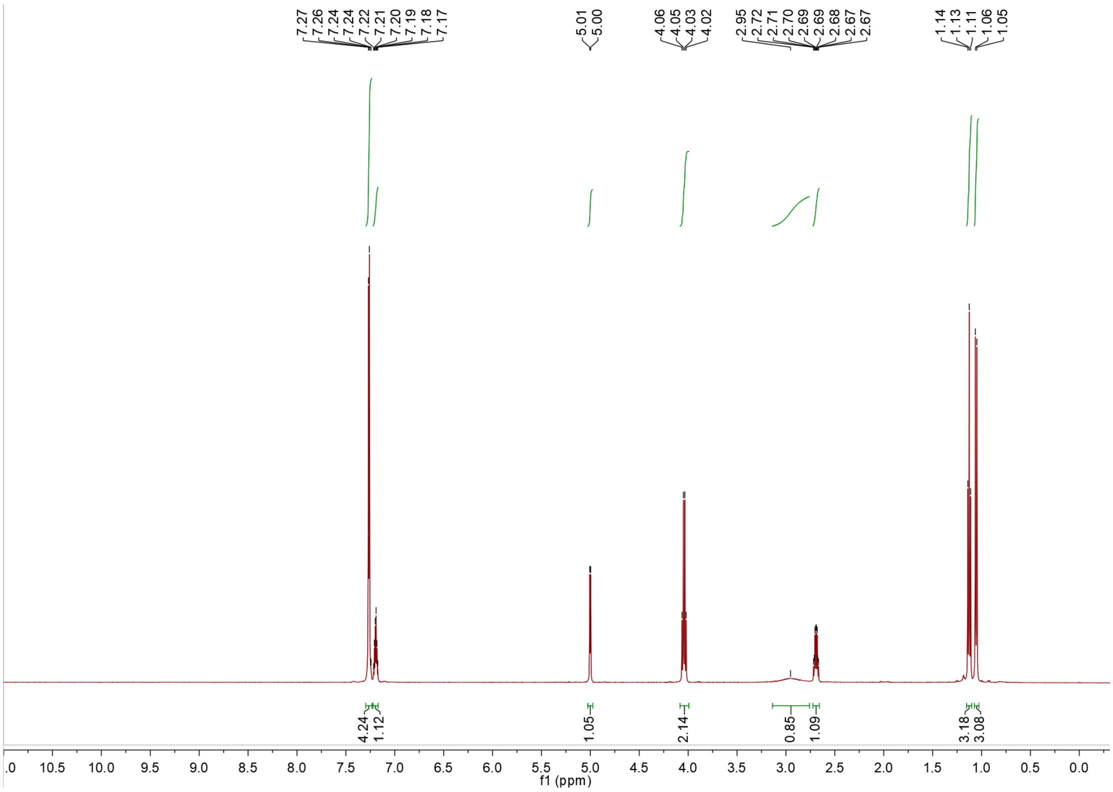
7.27
7.26
7.24
7.24
7.22
7.21
7.20
7.19
7.18
7.17
5.01
5.00
4.06
4.05
4.03
4.02
2.95
2.72
2.71
2.70
2.69
2.69
2.68
2.67
2.67
1.14
1.13
1.11
1.06
1.05
4.24
1.12
1.05
2.14
0.85
1.09
3.18
3.08
f1 (ppm)

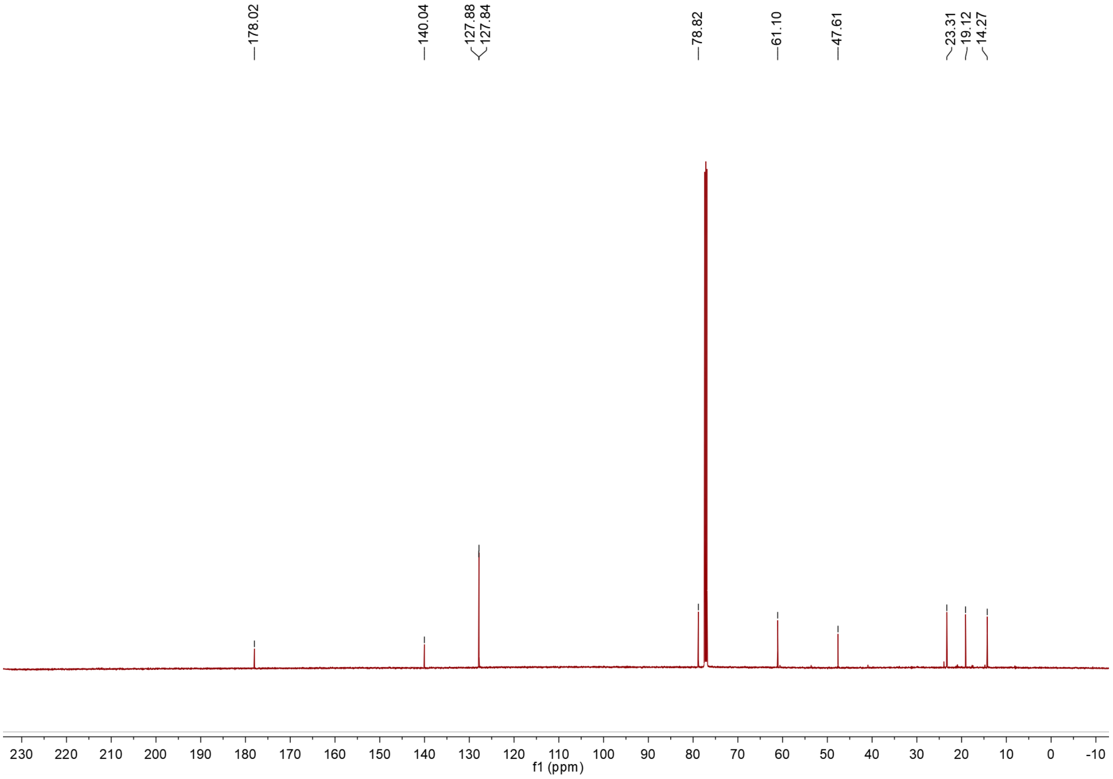
178.02
140.04
127.88
127.84
78.82
61.10
47.61
23.31
19.12
14.27
f1 (ppm)

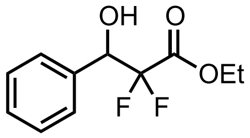
OH
O
OEt
F
F

19

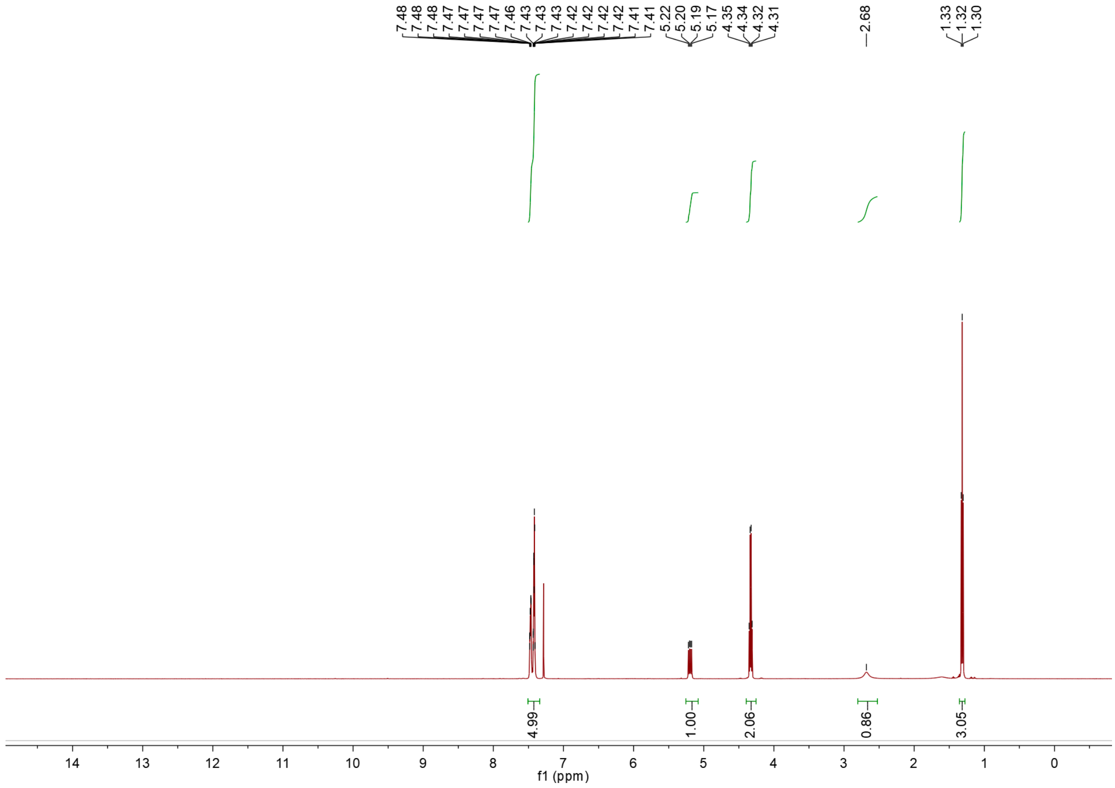
7.48
7.48
7.48
7.47
7.47
7.47
7.47
7.46
7.43
7.43
7.43
7.42
7.42
7.42
7.42
7.42
7.41
7.41
5.22
5.20
5.19
5.17
4.35
4.34
4.32
4.31
2.68
1.33
1.32
1.30
4.99
1.00
2.06
0.86
3.05
14
13
12
11
10
9
8
7
6
5
4
3
2
1
0
f1 (ppm)

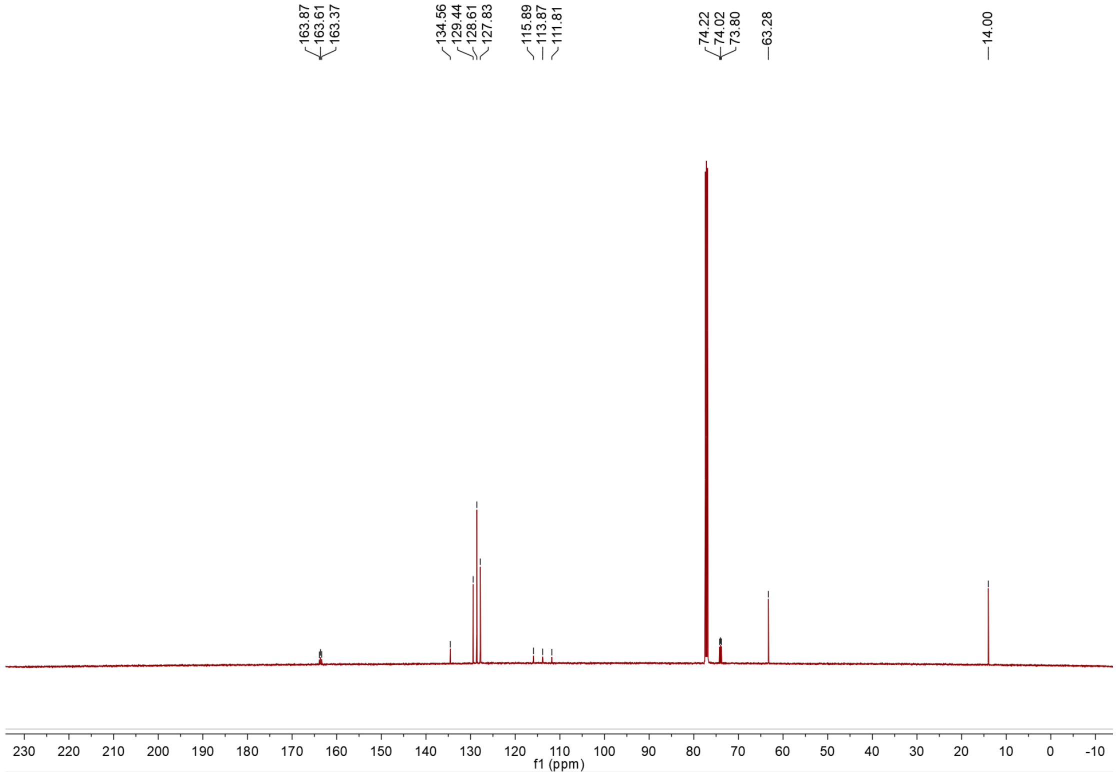
163.87
163.61
163.37
134.56
129.44
128.61
127.83
115.89
113.87
111.81
74.22
74.02
73.80
63.28
14.00
230
220
210
200
190
180
170
160
150
140
130
120
110
100
90
80
70
60
50
40
30
20
10
0
-10
f1 (ppm)

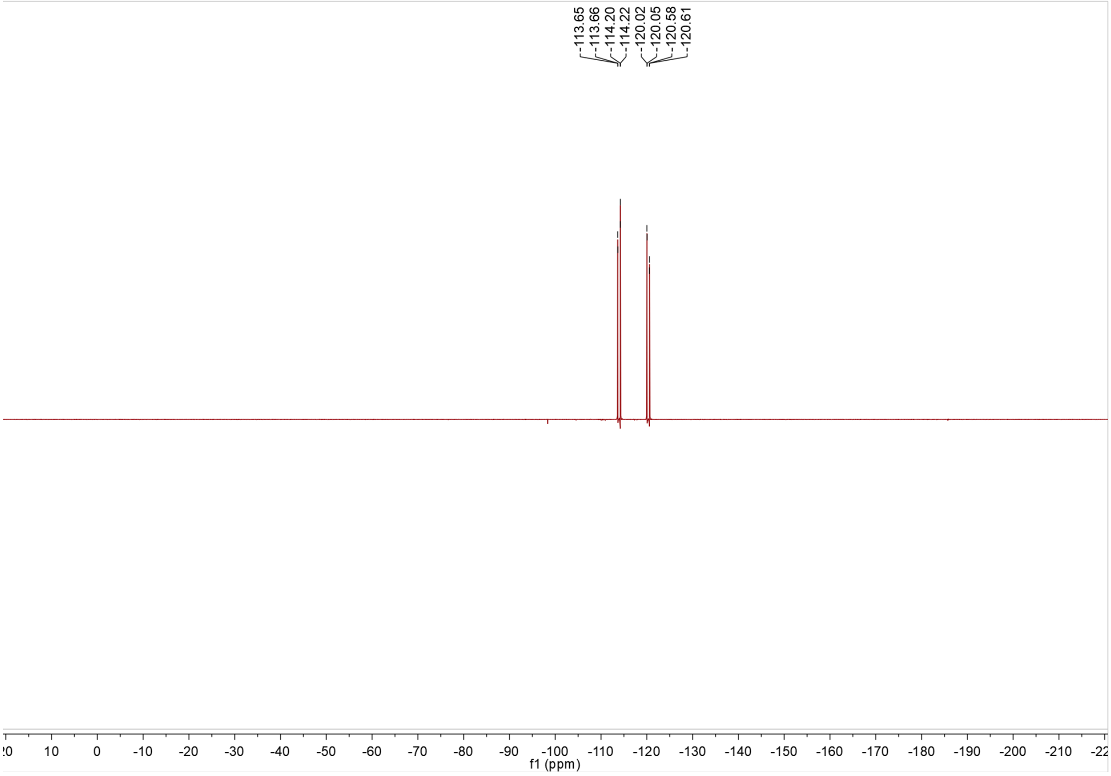
-113.65
-113.66
-114.20
-114.22
-120.02
-120.05
-120.58
-120.61
f1 (ppm)

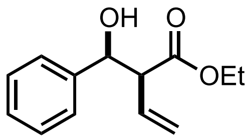
OH
O
OEt

20

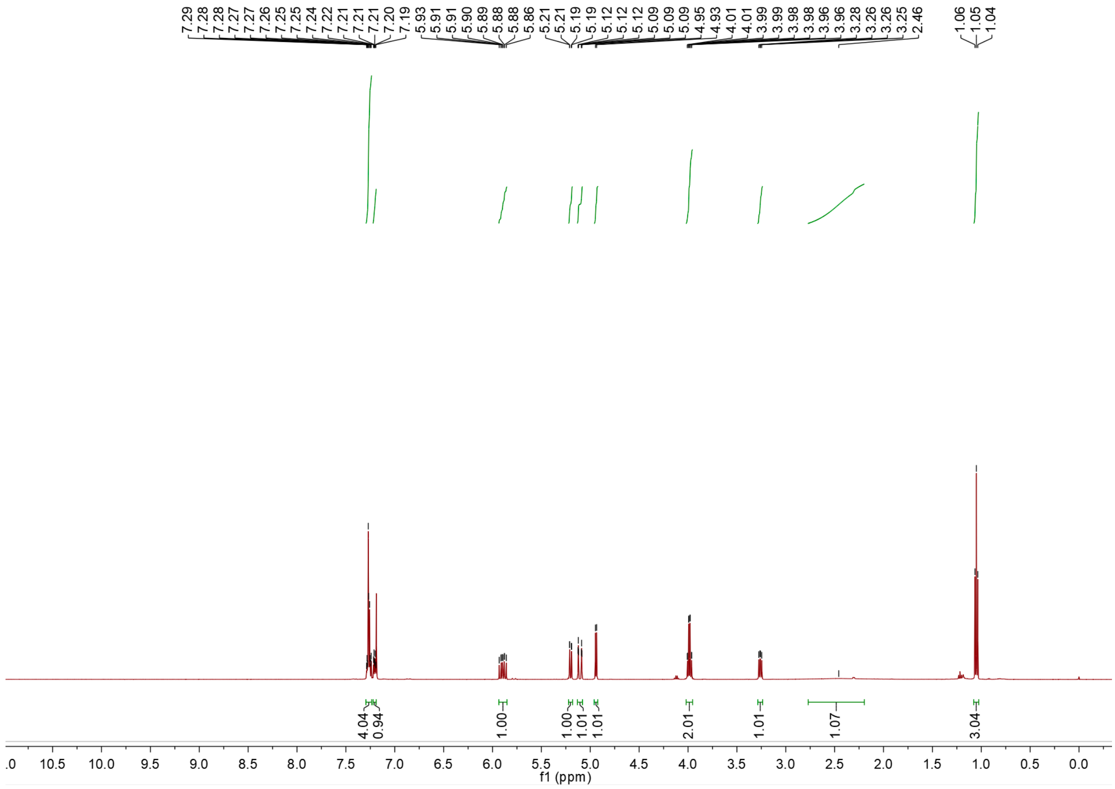
7.29
7.28
7.28
7.27
7.27
7.26
7.25
7.25
7.24
7.22
7.21
7.21
7.21
7.20
7.19
5.93
5.91
5.91
5.90
5.89
5.88
5.88
5.86
5.21
5.21
5.19
5.19
5.12
5.12
5.12
5.09
5.09
5.09
4.95
4.93
4.01
4.01
3.99
3.99
3.98
3.98
3.96
3.96
3.28
3.26
3.26
3.25
2.46
1.06
1.05
1.04
4.04
0.94
1.00
1.00
1.01
1.01
2.01
1.01
1.07
3.04
.0
10.5
10.0
9.5
9.0
8.5
8.0
7.5
7.0
6.5
6.0
5.5
5.0
4.5
4.0
3.5
3.0
2.5
2.0
1.5
1.0
0.5
0.0
f1 (ppm)

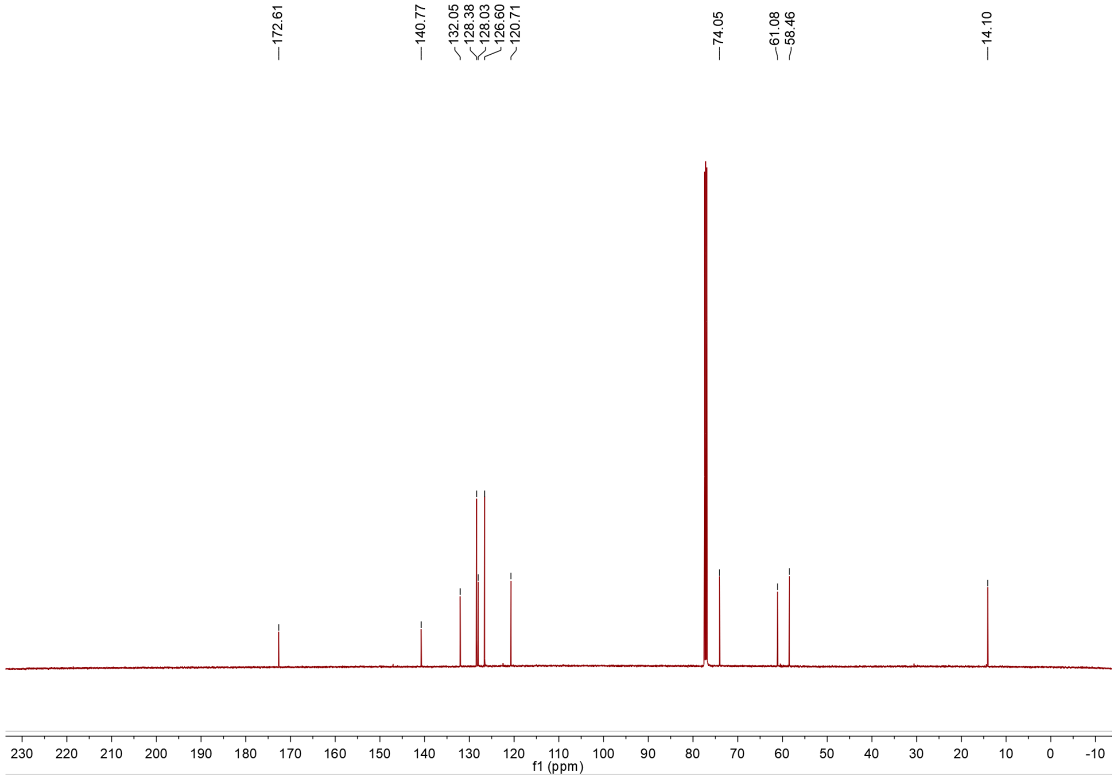
172.61
140.77
132.05
128.38
128.03
126.60
120.71
74.05
61.08
58.46
14.10
230
220
210
200
190
180
170
160
150
140
130
120
110
100
90
80
70
60
50
40
30
20
10
0
-10
f1 (ppm)

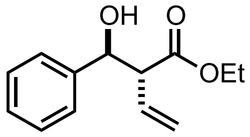

20

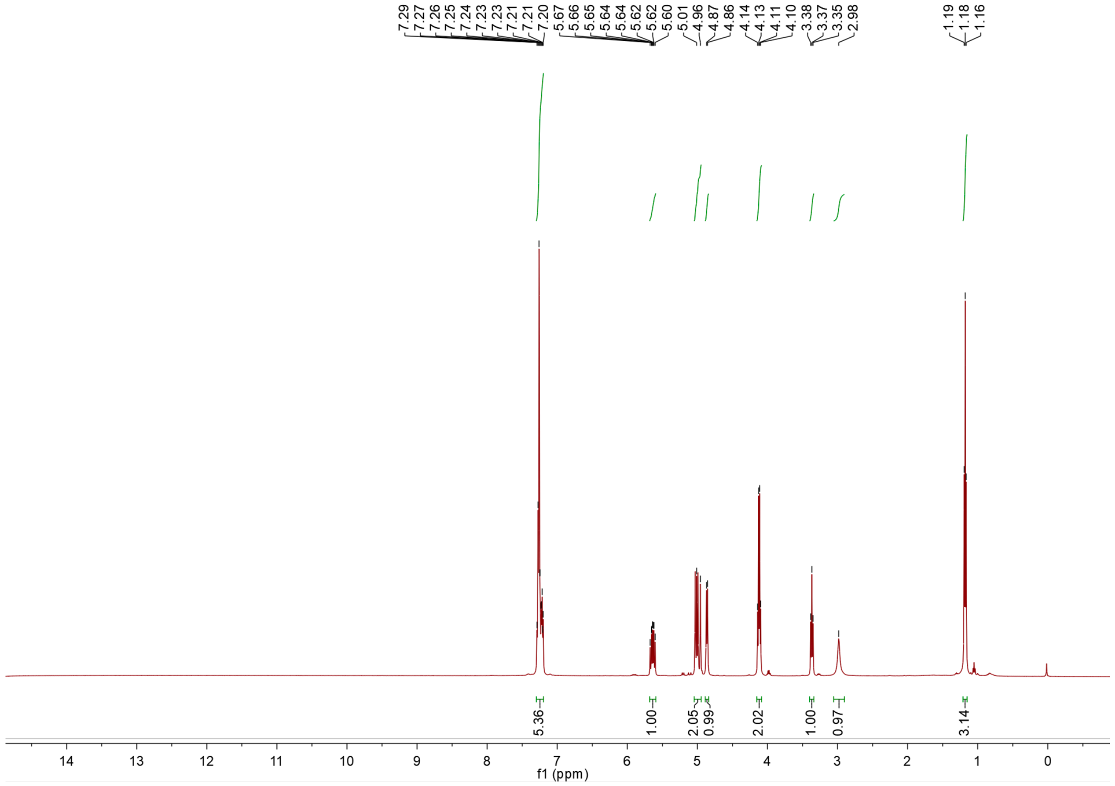

7.29
7.27
7.26
7.25
7.24
7.23
7.23
7.21
7.21
7.20
5.67
5.66
5.65
5.64
5.64
5.62
5.62
5.60
5.01
4.96
4.87
4.86
4.14
4.13
4.11
4.10
3.38
3.37
3.35
2.98
1.19
1.18
1.16
5.36
1.00
2.05
0.99
2.02
1.00
0.97
3.14
14
13
12
11
10
9
8
7
6
5
4
3
2
1
0
f1 (ppm)

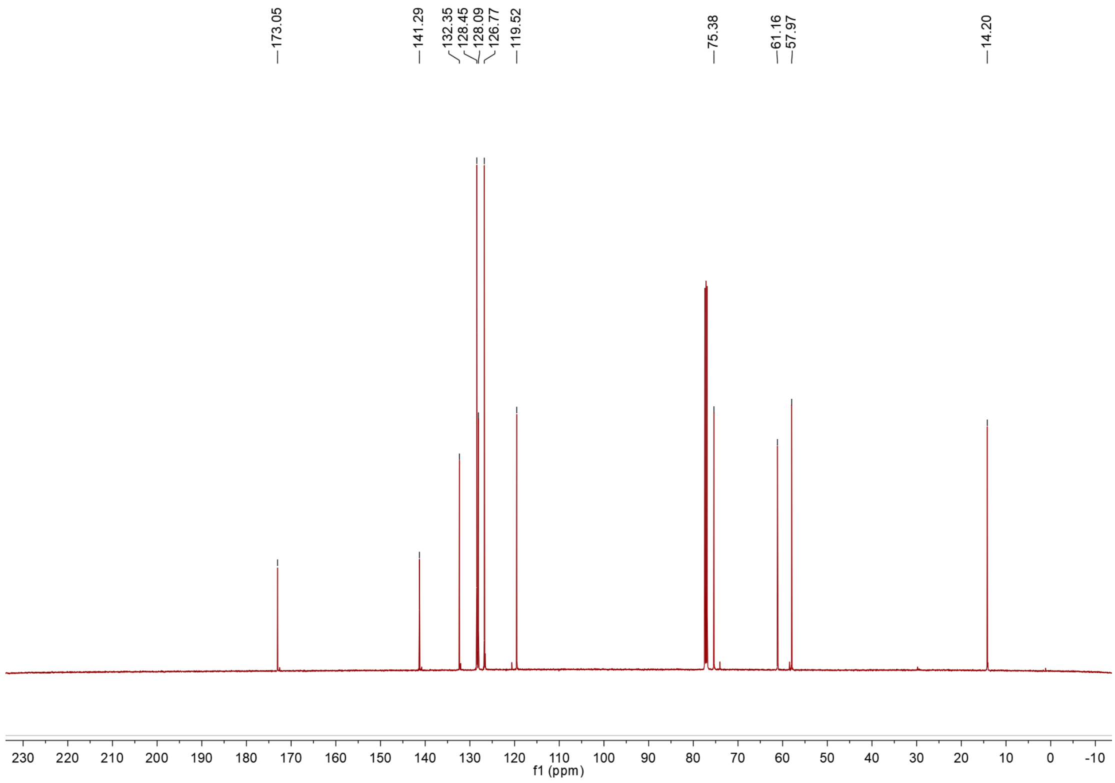

173.05
141.29
132.35
128.45
128.09
126.77
119.52
75.38
61.16
57.97
14.20
230
220
210
200
190
180
170
160
150
140
130
120
110
100
90
80
70
60
50
40
30
20
10
0
-10
f1 (ppm)

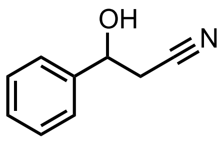
OH
N

21

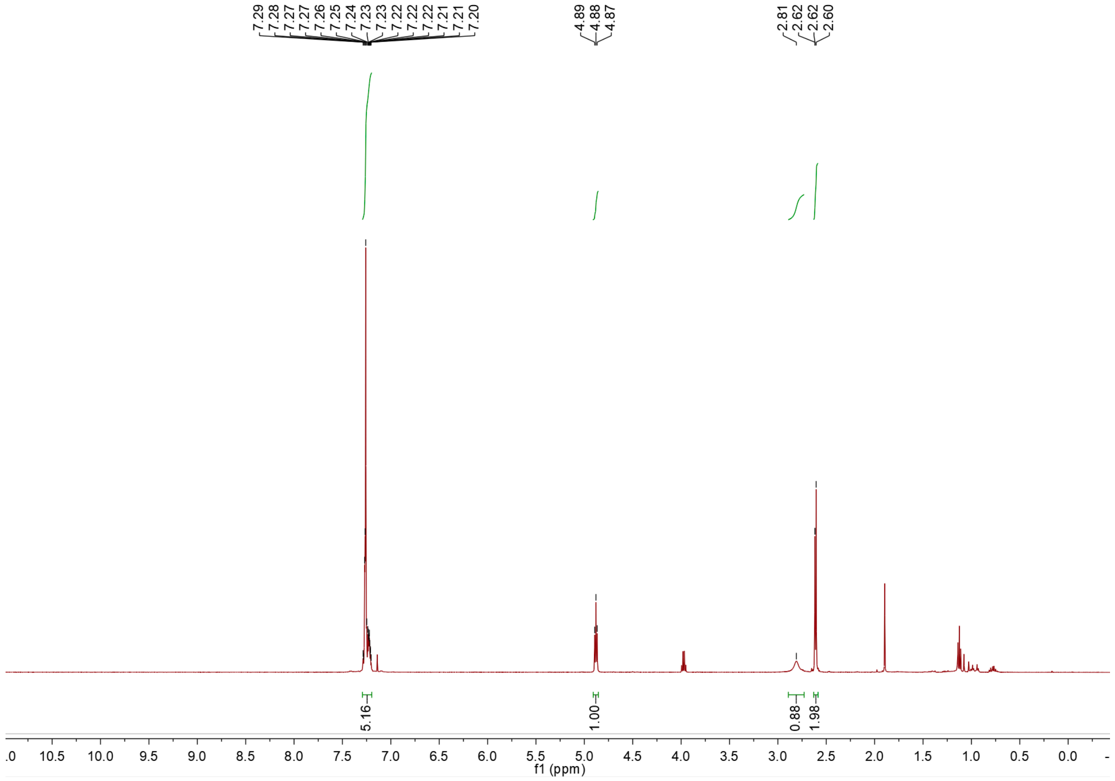
7.29 7.28 7.27 7.27 7.26 7.25 7.24 7.23 7.23 7.22 7.22 7.22 7.22 7.21 7.21 7.20
4.89 4.88 4.87
2.81 2.62 2.62 2.60
5.16
1.00
0.88
1.98
f1 (ppm)

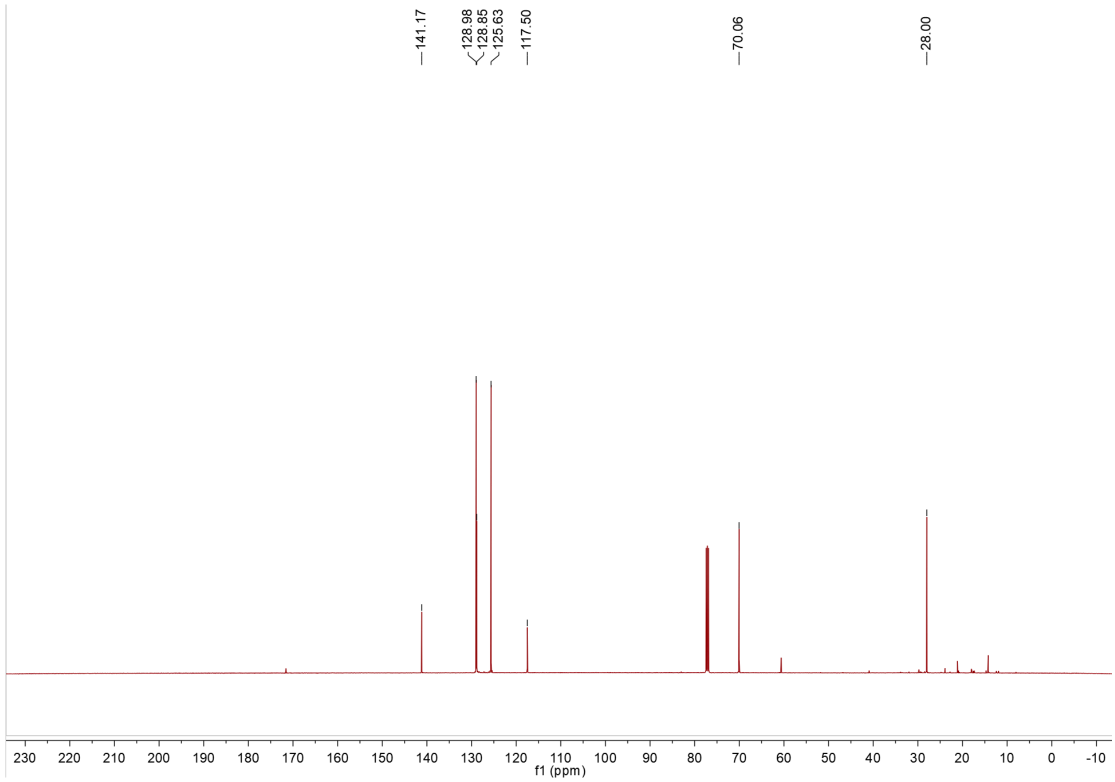
141.17
128.98 128.85 125.63
117.50
70.06
28.00
f1 (ppm)

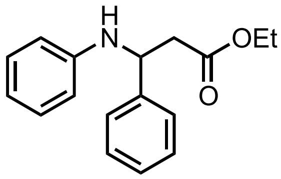

23

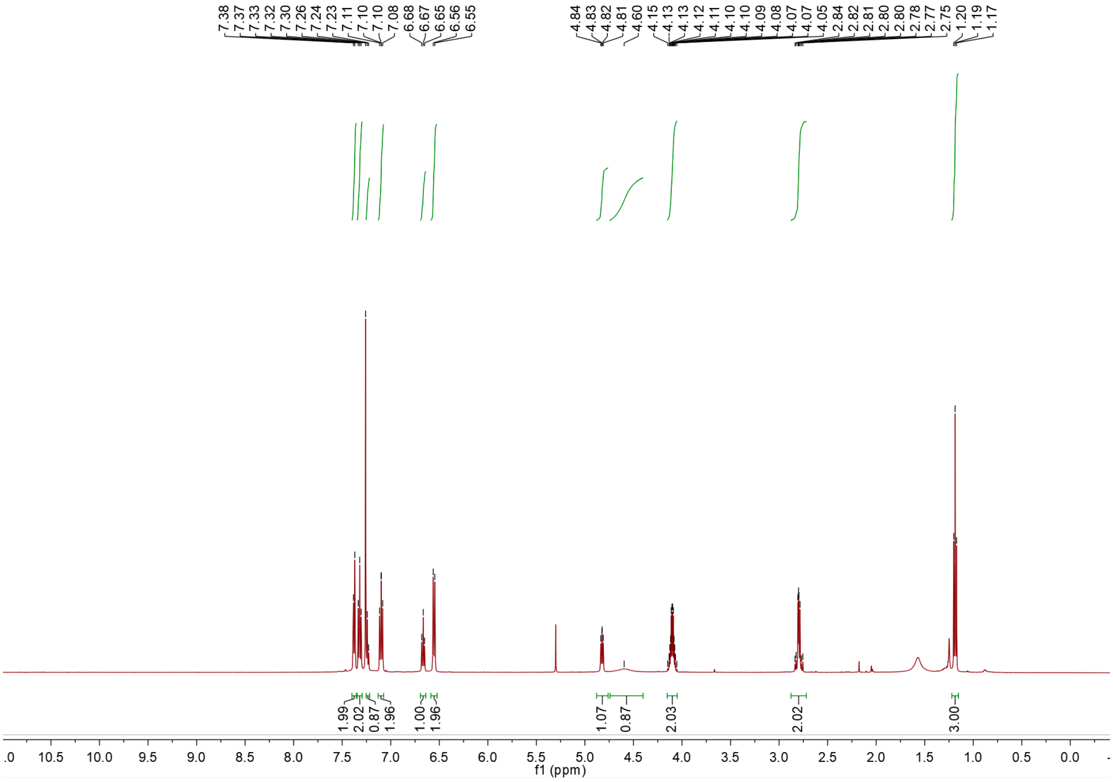

7.38 7.37 7.33 7.32 7.30 7.26 7.24 7.23 7.11 7.10 7.10 7.08 6.68 6.67 6.65 6.56 6.55
4.84 4.83 4.82 4.81 4.60 4.15 4.13 4.13 4.12 4.11 4.10 4.10 4.09 4.08 4.07 4.07 4.05 2.84 2.82 2.81 2.80 2.80 2.78 2.77 2.75 1.20 1.19 1.17
1.99 2.02 0.87 1.96 1.00 1.96 1.07 0.87 2.03 2.02 3.00
f1 (ppm)

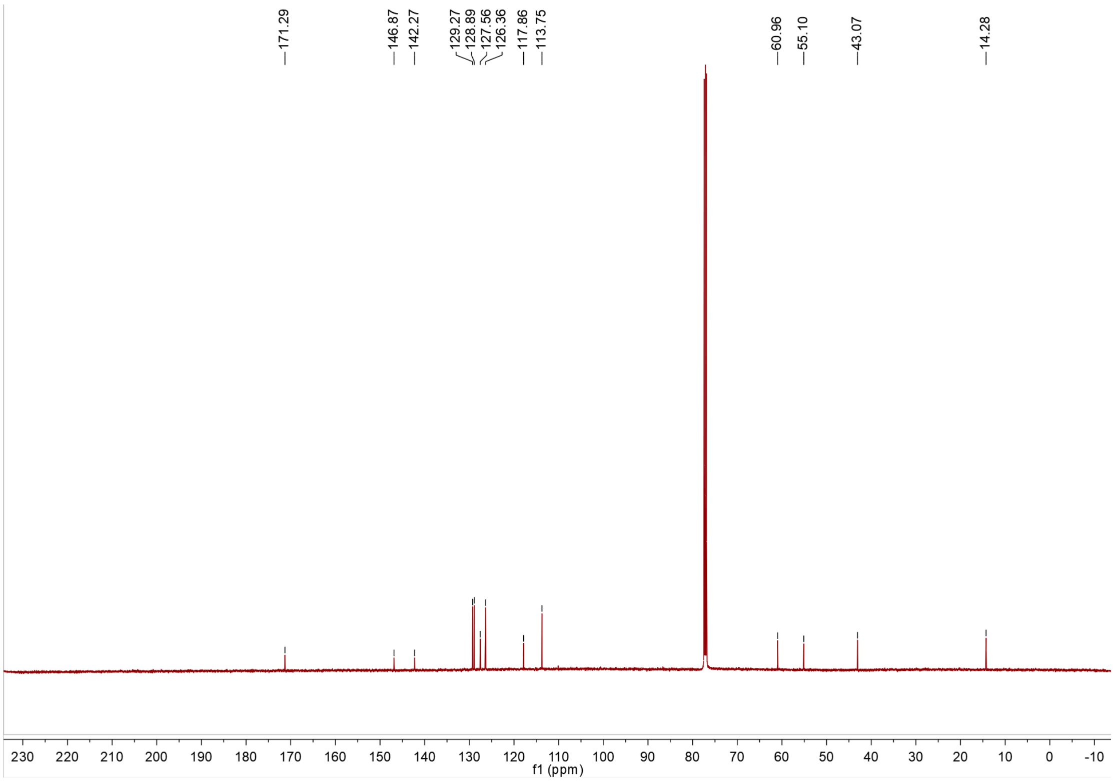
171.29
146.87
142.27
129.27
128.89
127.56
126.36
117.86
113.75
60.96
55.10
43.07
14.28
f1 (ppm)

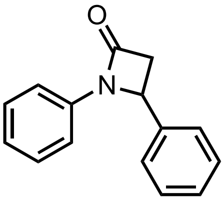
O
N

24

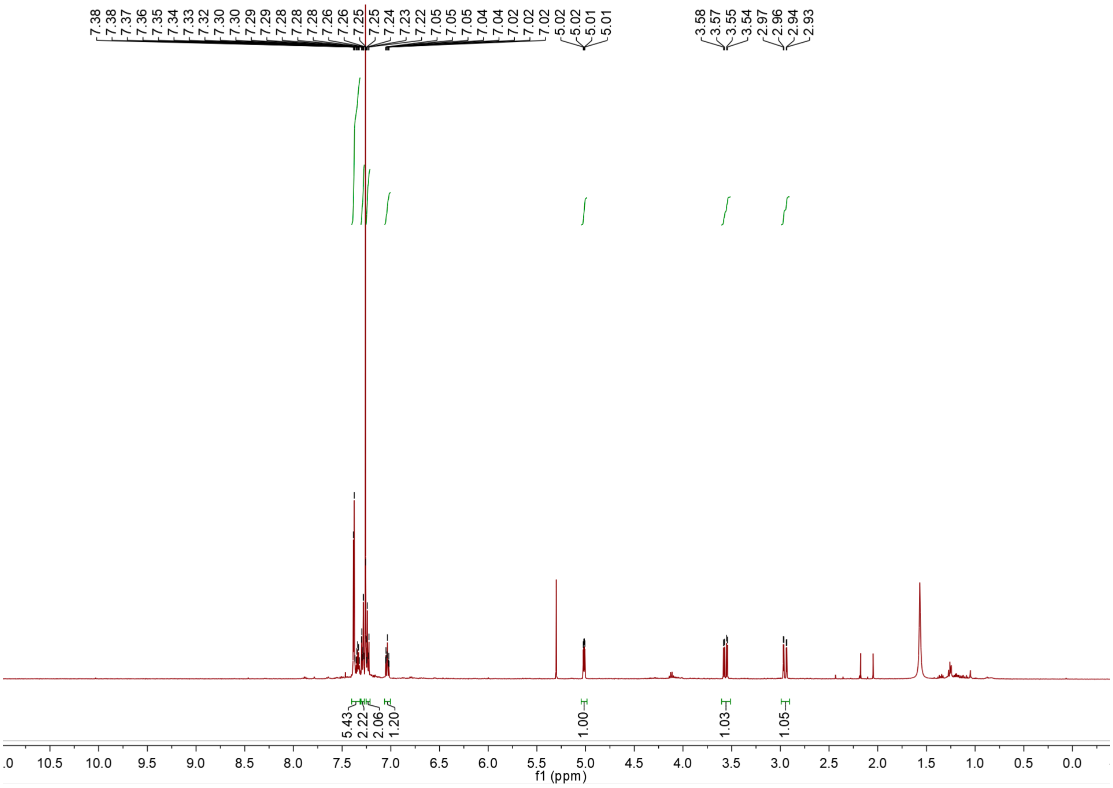
7.38
7.38
7.37
7.36
7.35
7.34
7.33
7.32
7.30
7.29
7.29
7.28
7.28
7.28
7.26
7.25
7.25
7.24
7.23
7.22
7.05
7.05
7.05
7.04
7.04
7.02
7.02
7.02
5.02
5.02
5.01
5.01
3.58
3.57
3.55
3.54
2.97
2.96
2.94
2.93
5.43
2.22
2.06
1.20
1.00
1.03
1.05
f1 (ppm)

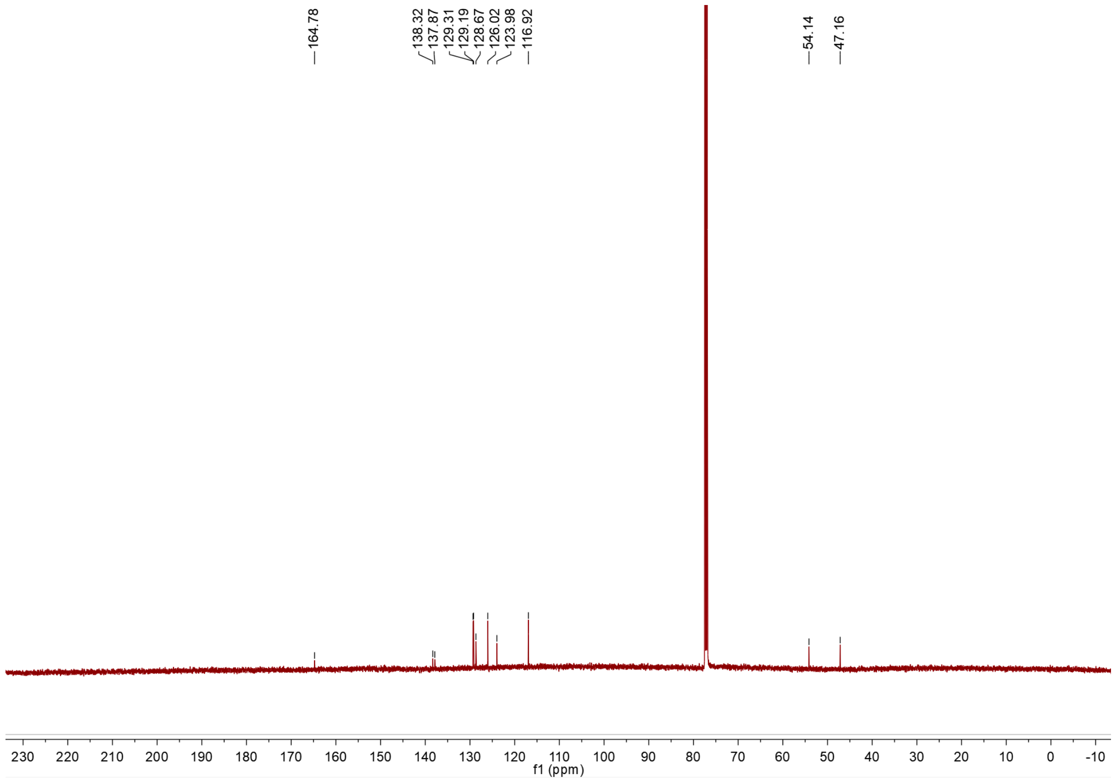

6 References

1 - X. Chen, C. Zhang, H. Wu, X. Yu, W. Su and J. Cheng, *Synthesis*, 2007, 3233–3239.

2 - M. A. Fernández-Ibáñez, B. Maciá, A. J. Minnaard and B. L. Feringa, *Angew. Chem. Int. Ed.*, 2008, **47**, 1317–1319.

3 - X.-L. Zou, G.-F. Du, W.-F. Sun, L. He, X.-W. Ma, C.-Z. Gu and B. Dai, *Tetrahedron*, 2013, **69**, 607–612.

4 - T. Taniguchi, Y. Sugiura, H. Zaimoku and H. Ishibashi, *Angew. Chem. Int. Ed.*, 2010, **49**, 10154–10157.

5 - A. Bernardi, L. Colombo Cesare Gennari and L. Prati, *Tetrahedron*, 1984, **40**, 3769–3775.

6 - J. Barluenga, F. J. Fananas and M. Yus, *J. Org. Chem.*, 1979, **44**, 4798–4801.

7 - L. Sgreccia, M. Bandini, S. Morganti, A. Quintavalla, A. Umani-Ronchi and P. G. Cozzi, *Journal of Organometallic Chemistry*, 2007, **692**, 3191–3197.

8 - P. J. Jervis and L. R. Cox, *Beilstein Journal of Organic Chemistry*, 2007, **3**, 6.

9 - (a) R. Ocampo, W. R. Dolbier, K. A. Abboud and F. Zuluaga, *J. Org. Chem.*, 2002, **67**, 72–78; (b) K. Mima, T. Ishihara, S. Kuwahata, T. Konno and H. Yamanaka, *Tetrahedron*, 2002, **58**, 2369–2376.

10 - I. Shiina, Y. Umezaki, N. Kuroda, T. Iizumi, S. Nagai and T. Katoh, *J. Org. Chem.*, 2012, **77**, 4885–4901.

11 - J. M. Andrés, M. A. Martínez, R. Pedrosa and A. Pérez-Encabo, *Synthesis*, 1996, 1070–1072.

12 - (a) J. Kister, D. H. Ess and W. R. Roush, *Org. Lett.*, 2013, **15**, 5436-5439; (b) P. V. Ramachandran, D. Nicponski and B. Kim, *Org. Lett.*, 2013, **15**, 1398-1401.

13 - K. Wadhwa and J. G. Verkade, *J. Org. Chem.*, 2009, **74**, 5683–5686.

14 - J. Tummatorn and G. B. Dudley, *Org. Lett.*, 2011, **13**, 158–160.

15 - S. Tang, J. He, Y. Sun, L. He and X. She, *J. Org. Chem.*, 2010, **75**, 1961–1966.
